# Characterizing the regulatory Fas (CD95) epitope critical for agonist antibody targeting and CAR-T bystander function in ovarian cancer

**DOI:** 10.1038/s41418-023-01229-7

**Published:** 2023-10-14

**Authors:** Tanmoy Mondal, Himanshu Gaur, Brice E. N. Wamba, Abby Grace Michalak, Camryn Stout, Matthew R. Watson, Sophia L. Aleixo, Arjun Singh, Salvatore Condello, Roland Faller, Gary Scott Leiserowitz, Sanchita Bhatnagar, Jogender Tushir-Singh

**Affiliations:** 1 Laboratory of Novel Biologics, University of California Davis, Davis, CA USA; 2 Department of Medical Microbiology and Immunology, University of California Davis, Davis, CA USA; 3 Undergraduate Research Program Volunteers, University of California Davis, Davis, CA USA; 4 Department of Obstetrics and Gynecology, Indiana University School of Medicine, Indianapolis, IN USA; 5 Department of Chemical Engineering, University of California Davis, Davis, CA USA; 6 Department of Obstetrics and Gynecology, UC Davis School of Medicine, Sacramento, CA USA; 7 UC Davis Comprehensive Cancer Center, UC Davis School of Medicine, Sacramento, CA USA; 8 Ovarian Cancer Academy Early Career Investigator at UC Davis, Davis, CA USA

**Keywords:** Tumour heterogeneity, Preclinical research, Cancer microenvironment

## Abstract

Receptor clustering is the most critical step to activate extrinsic apoptosis by death receptors belonging to the TNF superfamily. Although clinically unsuccessful, using agonist antibodies, the death receptors-5 remains extensively studied from a cancer therapeutics perspective. However, despite its regulatory role and elevated function in ovarian and other solid tumors, another tumor-enriched death receptor called Fas (CD95) remained undervalued in cancer immunotherapy until recently, when its role in off-target tumor killing by CAR-T therapies was imperative. By comprehensively analyzing structure studies in the context of the binding epitope of FasL and various preclinical Fas agonist antibodies, we characterize a highly significant patch of positively charged residue epitope (PPCR) in its cysteine-rich domain 2 of Fas. PPCR engagement is indispensable for superior Fas agonist signaling and CAR-T bystander function in ovarian tumor models. A single-point mutation in FasL or Fas that interferes with the PPCR engagement inhibited apoptotic signaling in tumor cells and T cells. Furthermore, considering that clinical and immunological features of the autoimmune lymphoproliferative syndrome (ALPS) are directly attributed to homozygous mutations in FasL, we reveal differential mechanistic details of FasL/Fas clustering at the PPCR interface compared to described ALPS mutations. As Fas-mediated bystander killing remains vital to the success of CAR-T therapies in tumors, our findings highlight the therapeutic analytical design for potentially effective Fas-targeting strategies using death agonism to improve cancer immunotherapy in ovarian and other solid tumors.

## Introduction

Death receptor-5 (DR5) and Fas receptor (Fas or FasR or CD95) belong to the tumor necrosis alpha (TNF) receptor superfamily and contain three external cysteine-rich domains (CRD1-3) and activate extrinsic apoptotic signaling [1]. Ligands for DR5 and Fas (Apo2L and FasL, respectively) and corresponding agonist antibodies orchestrate apoptotic signaling via assembling an activated death-inducing signaling complex (DISC) in a sophisticated process that requires higher-ordered clustering inside and outside the membrane [2]. However, the clustering mechanisms for both DR5 and Fas remained elusive until recently. Using clinical bivalent or multivalent monospecific antibodies, bispecific antibodies, receptor mutagenesis studies, functional assays, and NMR structure analysis of DR5, a series of papers have discovered and characterized receptor clustering conformation autoinhibitory motifs in the DR5 extracellular domain (ECD) [3, 4]. The key described motif comprises a cysteine-stabilized patch of positively charged (arginine, lysine) amino acid residues in DR5 CRD3. More importantly, monospecific and bispecific antibody-based strategies capable of tempering PPCR functions were most effective in DR5 clustering, signaling, and antitumor efficacy studies in ovarian and TNBC models [3–5].

Similar to DR5, levels of Fas are highly elevated in ovarian tumors; however, unlike DR5, Fas signaling has been an extensive focus of tumorigenic signaling [6] instead of an extrinsic apoptotic function, especially in ovarian and other solid tumors [7]. The latter has been attributed to the activation of reverse Fas signaling in contributing toward cell migration [8], cancer metastasis [9], cancer stem cell function [10], and cell survival pathways [11] in general. Significantly, unlike DR5, Fas signaling is critical in maintaining activation-induced cell death (AICD) of T lymphocytes for healthy immune homeostasis [12]. Considering the clinical response of immunotherapy is highly dependent on increased infiltration of immune effector T lymphocytes in solid tumors [13, 14], and the CAR-T efficacy being highly reliant on bystander FasL: Fas (CAR-T cell: Tumor cell) signaling and killing [15–17], it is highly imperative to uncover the most critical and regulatory targeting epitope of Fas receptor for the next generation of immunotherapeutic capable of selectively targeting tumors. In the present study, using ovarian cancer cellular and tumor model systems, we sought to investigate whether, similar to DR5, Fas exploits the negative regulatory PPCR motif for its optimal receptor clustering, signaling, and death activation by agonist antibodies and bystander CAR-T function. Furthermore, as the homogenous mutation in FasL has been attributed to autoimmune lymphoproliferative syndrome (ALPS) [18], we also asked if inherited nonlethal mutations in FasL would produce inhibition of Fas signaling, activation, and clustering similar to PPCR mutations in tumors and T cells.

## Results

### Conserved PPCR motif in Fas

**Fig. 1:**
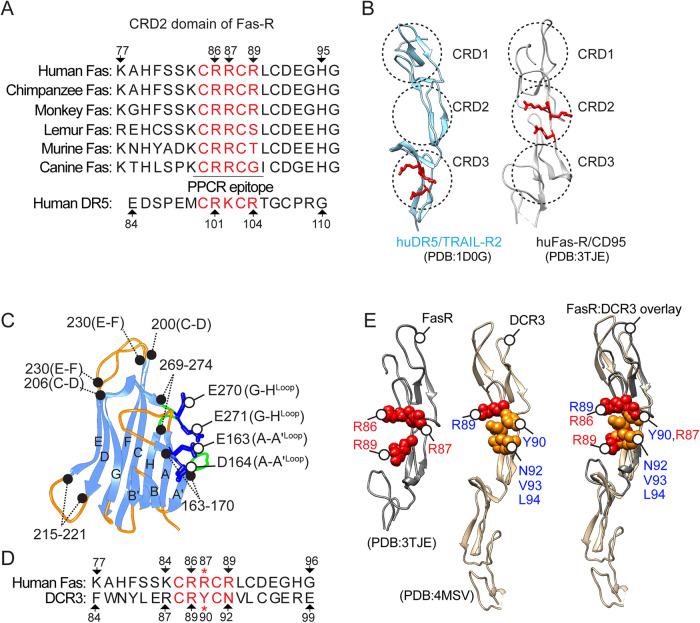
PPCR remains conserved in Fas and DR5. **A** Alignment of Fas PPCR region sequences from dog to humans. **B** Ribbon depiction of PPCR in DR5 and Fas. The three CRD′s are marked with dotted circles. **C** Jelly-like structure of FasL colored blue (beta-sheets) and orange (connecting loops). **D** Alignment of Fas PPCR region with the corresponding sequence in DCR3. **E** Overlay of DCR3 (gold, PDB:4MSV) and Fas (dark gray, PDB:3TJE). Positively charged residues are shown in red spheres. The critical R87 is replaced with Y90 in DCR3.

Using clinical DR5 agonist antibody lexatumumab and via mutational and functional assays, we have previously described the importance of salt bridges between negatively charged residues in lexatumumab heavy chain (VH) with the DR5 PPCR to bring receptor-activating conformational change [3]. A similar loss of apoptotic function was observed when PPCR interface residues of apomab and Apo2L were mutated (Supplementary Fig. S1). Upon DR5 and Fas ECD sequence alignment, a highly conserved cysteine-stabilized arginine-rich patch (similar to DR5 PPCR) [3], is evident in human, monkey, mouse, and dog receptors (Fig. 1A). Unlike DR5 PPCR, Fas PPCR is present in the middle of CRD2 instead of CRD3 (Fig. 1B). Unlike the characterization of DR5’s highly variable CRD3, the superimposition of Fas from just two published structures studies [19] (using weak Fas agonist antibodies sharing a common binding interface), it is difficult to conclude if Fas PPCR harboring CRD2 would also maintain conformation variability similar to DR5 [3]. The latter is because multiple research groups have independently studied DR5 structures with various clinical agonist antibodies targeting its distinct epitopes [5, 20–23]. However, no Fas antibody has been made to clinical trials. Hence, Fas has yet to be the focus of extensive structure studies in the context of clinically applicable agonist antibodies.

**Fig. 2:**
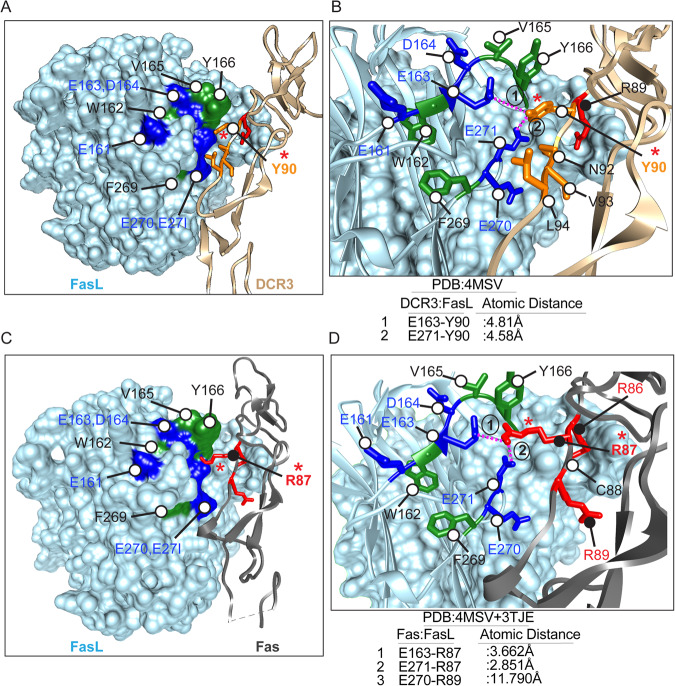
FasL forms salt bridges with Fas R87 residue. **A** A space-filling representation of FasL surface (sky blue) at the interface of PPCR corresponding region of DCR3 (gold color shown as ribbon structure). Blue and green surfaces in FasL highlight the negatively charged and hydrophobic residues, respectively. Fas PPCR corresponding residues of DCR3 are shown in red and orange sticks. **B** Zoomed interface of FasL and DCR3 at PPCR from (**A**). Y90 (orange stick near red asterisk) of DCR3 acts like a “key” to insert itself into hydrophobically stabilized “lock” of negatively charged residues (E163, E271). Additional hydrophobic residues of DCR3 at its Fas PPCR corresponding region are shown in orange. **C** Same as (**A**), except Fas (Gray ribbon) is overlayed instead of DCR3. Fas PPCR residues are shown in red sticks. **D** Zoomed interface of FasL and Fas at PPCR. Instead of tyrosine (Y90) in DCR3, R87 (red stick near red asterisk) of Fas form significantly close salt-bridges (3.66 and 2.85 Å) with FasL negatively charged residues E163 and E271. The guanidine side chains of R86 and R89 are oriented away from the FasL PPCR interface motif.

FasL binding interface with decoy receptor DCR3 has been published [24], and similar to other low-affinity TNF family ligands, FasL adopts a jelly-roll type (Similar to an Ig-fold) structure composed of antiparallel beta-strands connected via variable loops (Fig. 1C) [24]. The key binding interface of FasL and DCR3 involves AA′ (residues 163-170), DE (215–221), and GH (269–274) loops from FasL (Fig. 1C and Supplementary Fig. S2) and residues P75-R87, R89-Y90, N92, and L94 from CRD2 domain of DCR3 (Supplementary Fig. S2). We generated FasL: Fas complex by superimposing Fas over DCR3 from PDB entry 3TJE [19]. Next, we focused on the FasL interface near Fas PPCR (Fig. 1D, E and Supplementary Fig. S2). Similar to DCR3 positively charged arginine 89 [R89^(+)^] the corresponding R86^(+)^ of Fas forms hydrogen bonds with the Q220 and D221 of FasL (Figs. 1E and  2A–D and Supplementary Fig. S2G–I). However, strikingly R87^(+)^ of Fas forms salt bridges with negatively charged E271^(-)^ (GH loop) and E163^(-)^ (AA′ loop), while Y90 of DCR3 (at the exactly similar corresponding position and side-chain orientation) forms hydrophobic interactions with FasL Y166 (Fig. 2B vs. D, denoted with red asterisk). Notably, the Y90-Y166 interface sterically blocks the accessibility of FasL negatively charged residues E271^(-)^ and E163^(-)^ to engage Fas^R87(+)^. Similar salt bridges key for Apo2L (Apo2L: DR5, D203:R101) [21] and DR5 CRD3 region-targeting apomab antibody (Ab: DR5, D30/D31:K102) [5] are present (Supplementary Fig. S1A, B, D, E). Although tested using random alanine scanning of Apo2L earlier [25], we confirmed the total loss of DR5 activation function by apomab and lexatumumab via mutating any of two negatively charged residues (Supplementary Fig. S1C, F). If the salt-bridges between FasL: Fas (E271/E163:R87) with Fas PPCR are highly critical (next sections) DISC assembly and death agonism, the latter explains the FasL saturating function of DCR3 to inhibit ligand’s ability to engage Fas PPCR to activate extrinsic apoptotic signaling.

### Single-point mutations at PPCR interface of FasL render it ineffective

**Fig. 3:**
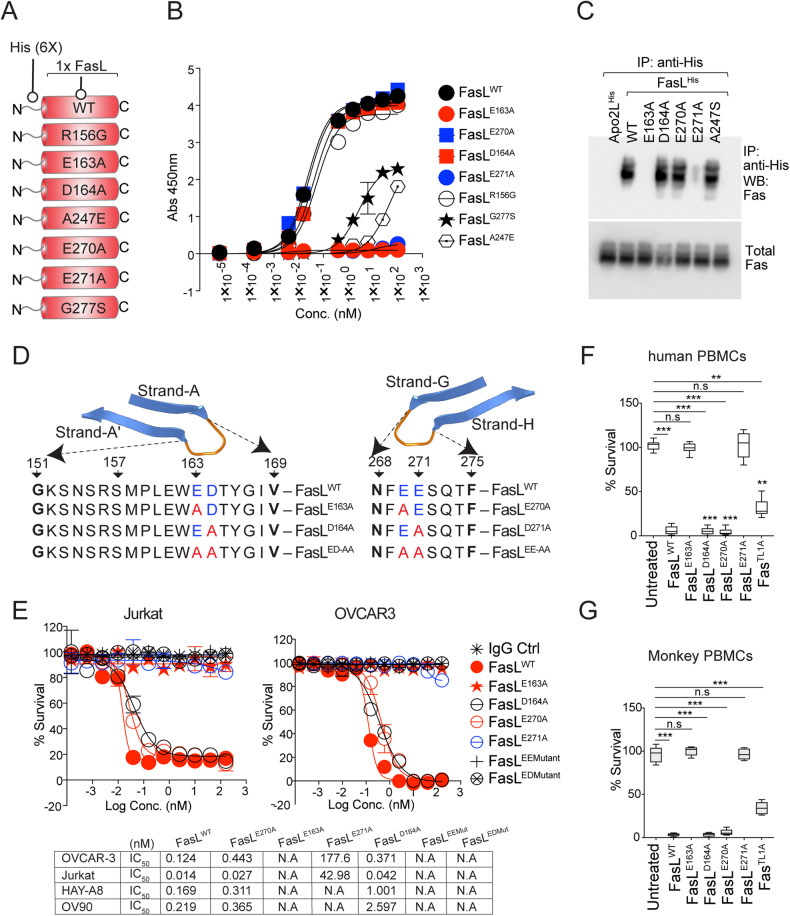
A single-point mutation in PPCR Interacting FasL Residues abolishes its function. **A** Schematic of various His-tagged FasL mutants described in the figure. **B** Binding analysis of His-tagged FasL to 96-well-immobilized Fc-tagged Fas by ELISA. **C** OVCAR3 cells were treated with indicated His-tagged FasL, followed by immunoprecipitation using anti-His antibody and immunoblotting using anti-Fas antibody. Total Fas levels were equal in all samples. **D** Schematic of FasL A-A′ and G-H beta loops with corresponding residues either WT or mutated (Red) in the loops are shown. Negatively charged residues are blue. **E** Cell-killing assay of OVCAR3 and Jurkat cells 36 h after treatment of indicated FasL mutants. IC_50_ values are shown at the bottom. **F**, **G** Cell survival assay of human and murine PBMC-derived and CD3-positive T cells 48 h after treatment of indicated FasL mutants. Error bars in (**B**), (**F**) and (**G**) represent SEM (*n* = 3+).

If adding a His tag could negatively interfere with FasL function, first, we compared N-terminal His-tagged FasL activity alongside commercial N-terminal Flag-tagged FasL. Both maintained similar apoptotic activity in Jurkat and tumor cell assays (Supplementary Fig. S3A). Next, considering the high yield and the ease of purification, to investigate further the importance of salt bridges formed by FasL E271^(-)^ and E163^(-)^ with Fas R87^(+)^ in PPCR (Fig. 2), we generated N-terminal His-tagged and C-terminal Fc-tagged FasL with E163A, D164A, E270A, E271A, ED163-164AA, EE270-271AA mutations (Fig. 3A, Supplementary Fig. S3B, and Table 1). An additional His-tagged FasL mutant having a cognate region of TL1A was also generated by replacing the c-terminal segment (DTYGIV-HELGLA called TL1A mutant) of AA’ loop as described previously (S3c) [24].

**Table 1:** Sequences of various His-tagged human and monkey FasL and Fas proteins used.

Recombinant FasL and Fas mutants	Sequences
His_FasL^WT^	HHHHHHHGGSPSPPPEKKELRKVAHLTGKSNSRSMPLEWEDTYGIVLLSGVKYKKGGLVINETGLYFVYSKVYFRGQSCNNLPLSHKVYMRNSKYPQDLVMMEGKMMSYCTTGQMWARSSYLGAVFNLTSADHLYVNVSELSLVNFEESQTFFGLYKL
His_FasL^TL1A^	HHHHHHHGGSPSPPPEKKELRKVAHLTGKSNSRSMPLEWEHELGLALLSGVKYKKGGLVINETGLYFVYSKVYFRGQSCNNLPLSHKVYMRNSKYPQDLVMMEGKMMSYCTTGQMWARSSYLGAVFNLTSADHLYVNVSELSLVNFEESQTFFGLYKL
His_FasL^E163A^	HHHHHHHGGSPSPPPEKKELRKVAHLTGKSNSRSMPLEW**A**DTYGIVLLSGVKYKKGGLVINETGLYFVYSKVYFRGQSCNNLPLSHKVYMRNSKYPQDLVMMEGKMMSYCTTGQMWARSSYLGAVFNLTSADHLYVNVSELSLVNFEESQTFFGLYKL
His_FasL^D163A^	HHHHHHHGGSPSPPPEKKELRKVAHLTGKSNSRSMPLEWE**A**TYGIVLLSGVKYKKGGLVINETGLYFVYSKVYFRGQSCNNLPLSHKVYMRNSKYPQDLVMMEGKMMSYCTTGQMWARSSYLGAVFNLTSADHLYVNVSELSLVNFEESQTFFGLYKL
His_FasL^E270A^	HHHHHHHGGSPSPPPEKKELRKVAHLTGKSNSRSMPLEWEDTYGIVLLSGVKYKKGGLVINETGLYFVYSKVYFRGQSCNNLPLSHKVYMRNSKYPQDLVMMEGKMMSYCTTGQMWARSSYLGAVFNLTSADHLYVNVSELSLVNF**A**ESQTFFGLYKL
His_FasL^E271A^	HHHHHHHGGSPSPPPEKKELRKVAHLTGKSNSRSMPLEWEDTYGIVLLSGVKYKKGGLVINETGLYFVYSKVYFRGQSCNNLPLSHKVYMRNSKYPQDLVMMEGKMMSYCTTGQMWARSSYLGAVFNLTSADHLYVNVSELSLVNFE**A**SQTFFGLYKL
His_FasL^R156G^	HHHHHHHGGSPSPPPEKKELRKVAHLTGKSNS**G**SMPLEWEDTYGIVLLSGVKYKKGGLVINETGLYFVYSKVYFRGQSCNNLPLSHKVYMRNSKYPQDLVMMEGKMMSYCTTGQMWARSSYLGAVFNLTSADHLYVNVSELSLVNFEESQTFFGLYKL
His_FasL^A247E^	HHHHHHHGGSPSPPPEKKELRKVAHLTGKSNSRSMPLEWEDTYGIVLLSGVKYKKGGLVINETGLYFVYSKVYFRGQSCNNLPLSHKVYMRNSKYPQDLVMMEGKMMSYCTTGQMWARSSYLG**E**VFNLTSADHLYVNVSELSLVNFEESQTFFGLYKL
His_FasL^G277S^	HHHHHHHGGSPSPPPEKKELRKVAHLTGKSNSRSMPLEWEDTYGIVLLSGVKYKKGGLVINETGLYFVYSKVYFRGQSCNNLPLSHKVYMRNSKYPQDLVMMEGKMMSYCTTGQMWARSSYLGAVFNLTSADHLYVNVSELSLVNFEESQTFF**S**LYKL
His_FasL^WT-WT^	HHHHHHHGGSPSPPPEKKELRKVAHLTGKSNSRSMPLEWEDTYGIVLLSGVKYKKGGLVINETGLYFVYSKVYFRGQSCNNLPLSHKVYMRNSKYPQDLVMMEGKMMSYCTTGQMWARSSYLGAVFNLTSADHLYVNVSELSLVNFEESQTFFGLYKLGGGSGGGSGGGSPSPPPEKKELRKVAHLTGKSNSRSMPLEWEDTYGIVLLSGVKYKKGGLVINETGLYFVYSKVYFRGQSCNNLPLSHKVYMRNSKYPQDLVMMEGKMMSYCTTGQMWARSSYLGAVFNLTSADHLYVNVSELSLVNFEESQTFFGLYKL
His_FasL^WT-R156G^	HHHHHHHGGSPSPPPEKKELRKVAHLTGKSNSRSMPLEWEDTYGIVLLSGVKYKKGGLVINETGLYFVYSKVYFRGQSCNNLPLSHKVYMRNSKYPQDLVMMEGKMMSYCTTGQMWARSSYLGAVFNLTSADHLYVNVSELSLVNFEESQTFFGLYKLGGGSGGGSGGGSPSPPPEKKELRKVAHLTGKSNS**G**SMPLEWEDTYGIVLLSGVKYKKGGLVINETGLYFVYSKVYFRGQSCNNLPLSHKVYMRNSKYPQDLVMMEGKMMSYCTTGQMWARSSYLGAVFNLTSADHLYVNVSELSLVNFEESQTFFGLYKL
His_FasL^WT-A247E^	HHHHHHHGGSPSPPPEKKELRKVAHLTGKSNSRSMPLEWEDTYGIVLLSGVKYKKGGLVINETGLYFVYSKVYFRGQSCNNLPLSHKVYMRNSKYPQDLVMMEGKMMSYCTTGQMWARSSYLGAVFNLTSADHLYVNVSELSLVNFEESQTFFGLYKLGGGSGGGSGGGSPSPPPEKKELRKVAHLTGKSNSRSMPLEWEDTYGIVLLSGVKYKKGGLVINETGLYFVYSKVYFRGQSCNNLPLSHKVYMRNSKYPQDLVMMEGKMMSYCTTGQMWARSSYLG**E**VFNLTSADHLYVNVSELSLVNFEESQTFFGLYKL
His_FasL^WT-G277S^	HHHHHHHGGSPSPPPEKKELRKVAHLTGKSNSRSMPLEWEDTYGIVLLSGVKYKKGGLVINETGLYFVYSKVYFRGQSCNNLPLSHKVYMRNSKYPQDLVMMEGKMMSYCTTGQMWARSSYLGAVFNLTSADHLYVNVSELSLVNFEESQTFFGLYKLGGGSGGGSGGGSPSPPPEKKELRKVAHLTGKSNSRSMPLEWEDTYGIVLLSGVKYKKGGLVINETGLYFVYSKVYFRGQSCNNLPLSHKVYMRNSKYPQDLVMMEGKMMSYCTTGQMWARSSYLGAVFNLTSADHLYVNVSELSLVNFEESQTFF**S**LYKL
His_FasL^WT-E163A^	HHHHHHHGGSPSPPPEKKELRKVAHLTGKSNSRSMPLEWEDTYGIVLLSGVKYKKGGLVINETGLYFVYSKVYFRGQSCNNLPLSHKVYMRNSKYPQDLVMMEGKMMSYCTTGQMWARSSYLGAVFNLTSADHLYVNVSELSLVNFEESQTFFGLYKLGGGSGGGSGGGSPSPPPEKKELRKVAHLTGKSNSRSMPLEW**A**DTYGIVLLSGVKYKKGGLVINETGLYFVYSKVYFRGQSCNNLPLSHKVYMRNSKYPQDLVMMEGKMMSYCTTGQMWARSSYLGAVFNLTSADHLYVNVSELSLVNFEESQTFFGLYKL
His_FasL^WT-D164A^	HHHHHHHGGSPSPPPEKKELRKVAHLTGKSNSRSMPLEWEDTYGIVLLSGVKYKKGGLVINETGLYFVYSKVYFRGQSCNNLPLSHKVYMRNSKYPQDLVMMEGKMMSYCTTGQMWARSSYLGAVFNLTSADHLYVNVSELSLVNFEESQTFFGLYKLGGGSGGGSGGGSPSPPPEKKELRKVAHLTGKSNSRSMPLEWE**A**TYGIVLLSGVKYKKGGLVINETGLYFVYSKVYFRGQSCNNLPLSHKVYMRNSKYPQDLVMMEGKMMSYCTTGQMWARSSYLGAVFNLTSADHLYVNVSELSLVNFEESQTFFGLYKL
His_FasL^WT-E270A^	HHHHHHHGGSPSPPPEKKELRKVAHLTGKSNSRSMPLEWEDTYGIVLLSGVKYKKGGLVINETGLYFVYSKVYFRGQSCNNLPLSHKVYMRNSKYPQDLVMMEGKMMSYCTTGQMWARSSYLGAVFNLTSADHLYVNVSELSLVNFEESQTFFGLYKLGGGSGGGSGGGSPSPPPEKKELRKVAHLTGKSNSRSMPLEWEDTYGIVLLSGVKYKKGGLVINETGLYFVYSKVYFRGQSCNNLPLSHKVYMRNSKYPQDLVMMEGKMMSYCTTGQMWARSSYLGAVFNLTSADHLYVNVSELSLVNF**A**ESQTFFGLYKL
His_FasL^WT-E271A^	HHHHHHHGGSPSPPPEKKELRKVAHLTGKSNSRSMPLEWEDTYGIVLLSGVKYKKGGLVINETGLYFVYSKVYFRGQSCNNLPLSHKVYMRNSKYPQDLVMMEGKMMSYCTTGQMWARSSYLGAVFNLTSADHLYVNVSELSLVNFEESQTFFGLYKLGGGSGGGSGGGSPSPPPEKKELRKVAHLTGKSNSRSMPLEWEDTYGIVLLSGVKYKKGGLVINETGLYFVYSKVYFRGQSCNNLPLSHKVYMRNSKYPQDLVMMEGKMMSYCTTGQMWARSSYLGAVFNLTSADHLYVNVSELSLVNFE**A**SQTFFGLYKL
Monkey His-FasL	HHHHHHHGGSQLFHLQKELAELRESTSQKHTASSLEKQIGHPSPPPEKKEQRKVAHLTGKPNSRSMPLEWEDTYGIVLLSGVKYKKGGLVINETGLYFVYSKVYFRGQSCTNLPLSHKVYMRNSKYPQDLVMMEGKMMSYCTTGQMWAHSSYLGAVFNLTSADHLYVNVSELSLVNFEESQTFFGLYKL
Monkey His-Fas	QVTDISSKGFELRKIVTTIETQNLEGLHHEGQFCRNPCPPGERKARDCTVNEDEPDCVPCQEGKEYTDKGHFSSKCRRCRLCDEGHGLEVEINCTRTQNTKCRCKPNFFCNSAVCEHCDPCTKCEHGIIEECTLTSNTKCKEEDSRSDSGGGSHHHHHHH
His-Fas	RLSSKSVNAQVTDINSKGLELRKTVTTVETQNLEGLHHDGQFCHKPCPPGERKARDCTVNGDEPDCVPCQEGKEYTDKAHFSSKCRRCRLCDEGHGLEVEINCTRTQNTKCRCKPNFFCNSTVCEHCDPCTKCEHGIIKECTLTSNTKCKEEGSRSNESGGGSGGSHHHHHHHHH
His-Fas^R87A^	RLSSKSVNAQVTDINSKGLELRKTVTTVETQNLEGLHHDGQFCHKPCPPGERKARDCTVNGDEPDCVPCQEGKEYTDKAHFSSKCR**A**CRLCDEGHGLEVEINCTRTQNTKCRCKPNFFCNSTVCEHCDPCTKCEHGIIKECTLTSNTKCKEEGSRSNESGGGSGGSHHHHHHHHH

When tested FasL^D164A^, FasL^E270A^ bound to Fas similar to FasL^WT^ in ELISA (Fig. 3B) and immunoprecipitation assays (Fig. 3C and Supplementary Fig. S3D) and showed cytotoxic activity against tumor cells, Jurkat cells and T cells similar to FasL^WT^ (Fig. 3D–G and Supplementary Fig. S3E). On the other hand, FasL^E163A^, FasL^E271A^, FasL^ED163-164AA^, and FasL^EE270-271AA^ mutants lost both binding (S3b,c), apoptotic cytotoxicity, and Fas clustering activity against tumor and T cells (Fig. 3D–G and Supplementary Fig. S3D–F). Notably, the TL1A mutant lost minimal binding (Supplementary Fig. S3C) and maintained >70% cytotoxic activity (Fig. 3F, G). We also confirmed the importance of negatively charged residues E163 and E271 via engineering C-terminal Fc-tagged FasL mutants (Supplementary Fig. S4A–C). It must be noted that N-terminal FasL conjugated with IgG1 Fc was not expressed; hence, we engineered and expressed FasL similar to scFv by genetically ligating it with the CH3 domain of idarucizumab IgG (antibody also named dabigatran or DB that targets anticoagulant medication Pradaxa) as described earlier by our group [26] (Supplementary Fig. S4A). Similar to His-tagged FasL, idarucizumab (DB) Fc-conjugated FasL (DB-IgG1-Fc-FasL or DB-FasL in short) with E163A and E271A mutations were completely unfunctional (Supplementary Fig. S4C). Since only a single E163 residue in A-A′ loop right next to the TL1A region (D164-V169) directly engages R87 of PPCR (see Fig. 2D), and its point mutation (but not of the whole TL1A region) renders FasL completely ineffective, it confirms the importance of PPCR engagement for Fas activation. To additionally reverse confirm the significance of PPCR engagement for FasL function, we transiently transfected Fas-KO HeyA8 cells (kindly provided by Dr Marcus Peter) with Fas^WT^, Fas^S83A^, Fas^K84A^, Fas^R86A^, Fas^R87A^, Fas^R89A^ mutant constructs under CMV promoter, followed by FasL treatment (Supplementary Fig. S4D). As evident by the caspase-8 activity assay, only Fas^R87A^ mutation rendered Fas utterly insensitive to FasL, while most other mutations maintained ~25–80% caspase-8 activation in comparison to wildtype FasL (Supplementary Fig. S4E).

**Fig. 4:**
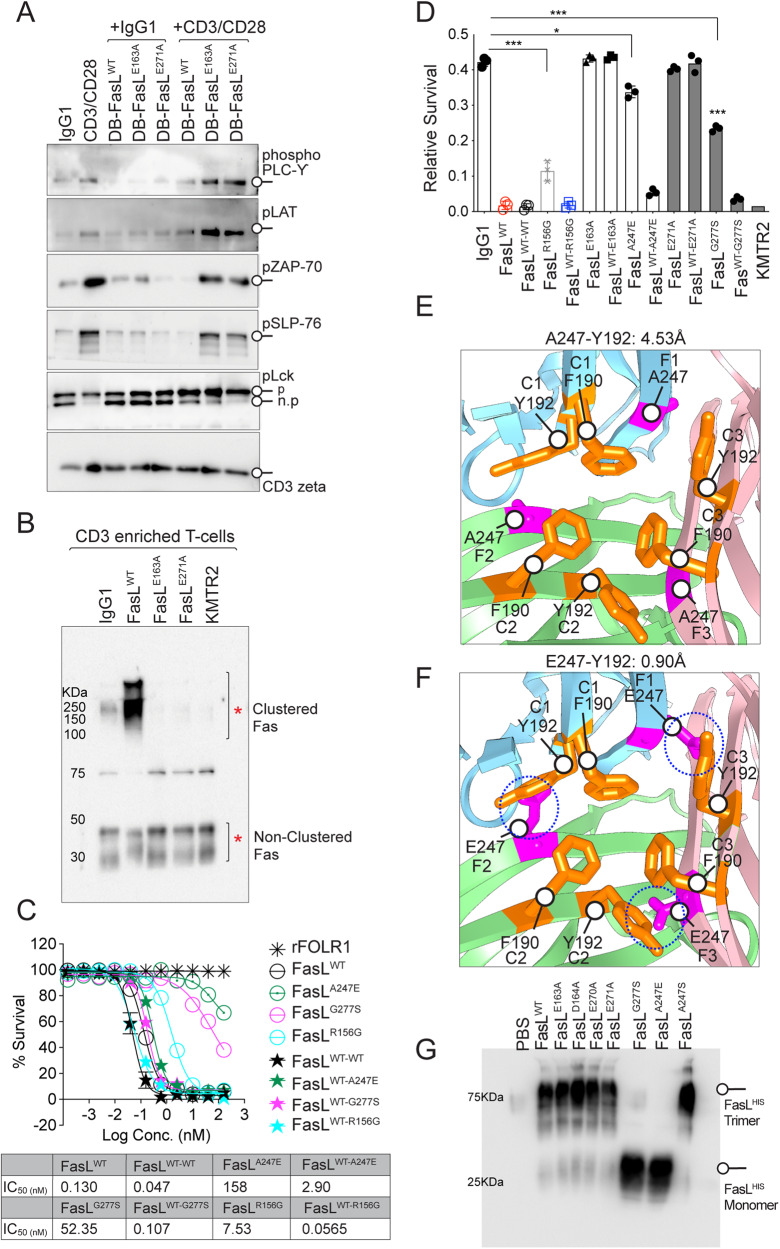
Differential Fas trimerization and activation profile by ALPS and PPCR mutations. **A** CD3-enriched human PBMC-derived T cells were treated with either IgG1 alone or CD3/CD28 agonist alone or indicated Fas-ligand mutants (DB-FasL) alone or together for 60 min (as indicated), followed by immunoblotting of indicated T-cell scaffold proteins and kinases. **B** CD3-enriched human PBMC-derived T cells were treated as indicated on top (DB-FasL mutants) for 1 h, followed by BS3 crosslinker treatment to capture the membrane-clustered Fas. Lysates were run in a non-reducing and partially denaturing gel and immunoblotted for Fas. **C** Jurkat cell killing assay of indicated FasL APLS mutants. rFOLR1 is a negative control. IC50 values are shown. **D** Same as (**C**), except human PBMC-derived CD3-enriched pan-T cells were used after activation with CD3/CD28 antibodies. **E** Ribbon diagram showing zoomed FasL trimer interface in sky blue (1), green (2), and pink (3) colors (PDB: 4MSV). Hydrophobic residues Y192 and F190 (colored orange and represented with sticks) on the C beta-strand of three FasL monomers (C1, C2, and C3) are shown. A247, with a short sidechain, is on the F beta-strands of three FasL monomers (F1, F2, and F3) and is colored pink and represented with sticks. **F** Same as (**E**), except the ALPS mutant E247 (substituted with A247 of the F beta-strands of three FasL monomers) with sidechain potentially sterically clashing with Y192 (on the C beta-strands of other FasL monomers) is shown in pink sticks. **G** Indicated FasL mutants on top were added to Jurkat cells in culture wells. After 30 min, soup and total lysates were pelleted together, followed by SDS-PAGE on non-reducing and partially denaturing gel and immunoblotting with anti-His antibody to detect recombinant FasL trimer formation on cells. Error bars in (**D**) represent SEM (*n* = 3+).

Due to its well-established role in AICD, T-cell homeostasis, and toxicity concerns against T cells, unlike DR5, Fas agonist antibodies have not been made to human clinical trials. One key event during ACID initiation involves the downregulation of T-cell proliferation [27]. As a result, when triggered either by membrane-bound FasL-expressing antigen-presenting cells or by recombinant soluble FasL, it inhibits the activation of various scaffolds and kinases downstream of the TCR-CD3 signaling complex [27]. Does FasL PPCR interface binding mutation also render it ineffective against ACID function? To this end, human PBMC-derived pan-T cells were stimulated with anti-CD3/CD28 agonist antibodies in the presence of either DB-IgG1-Fc-FasL^WT^ or harboring mutations against the PPCR interface (Supplementary Fig. S4A). Triggering of T-cell Fas with FasL^WT^ during CD3/CD28 agonists (OKT3/TGN1412) mediated priming interfered with TCR signaling by inhibiting tyrosine phosphorylation of ζ-chain–associated protein of 70 kD (ZAP70), phospholipase-γ (PLC-γ), and protein kinase C, etc. (Fig. 4A). On the contrary addition of FasL^E163A^, FasL^E271A^ mutants did not inhibit TCR activation (Fig. 4A). At the same time, non-PPCR-engaging FasL mutations (D163A, E270A, TL1A mutants) right next to E163A, E271A effectively blocked the TCR activation cascade and Fas clustering of T cells (Fig. 4A, B). Exactly similar results were obtained when cross-species specific anti-CD3 antibody (F12Q) and CD28 agonists (TGN1412) were used to stimulate monkey PBMC, followed by treatment with WT and various FasL mutants (Supplementary Fig. S4F).

### Differential Fas activation profile of ALPS and PPCR mutations

Patients with the ALPS carry either homozygous or heterozygous missense mutations that negatively affect or abolish FasL protein activity to eliminate lymphocytes [28]. A key marker of ALPS is a high proportion of CD4^−^CD8^−^ TCRαβ+ T cells, called double-negative T cells, and increased plasma concentrations of soluble Fas-ligand with limited cytotoxicity potential to eliminate T cells. Several homozygous missense mutations [18, 29] in FasL, such as G277S [30], A247E [31], R156G [32], etc., have been attributed to APLS. When tested, unlike PPCR interface FasL mutants (PPCR IFMs), such as E163A, E271A, the described FasL mutations in ALPS patients [such as G277S, A247E, R156G] partially activated cell death of both tumor and Jurkat cells (Fig. 4C, D).

FasL trimer formation is guided by hydrophobic interactions and interlocking of protruding Y190 and F192 into the non-hydrophobic pocket of 3 monomers (Supplementary Fig. S5A). Based on FasL: DCR3 structures study (PDB:4MSV), it is evident that A247E mutation directly interferes with the noncovalent trimer assembly (Fig. 4E). Substitution of a short side-chain alanine (A) at 274 by glutamine (E) brings its longer guanidine side-chain less than 0.9 Å closer to Y192 (Fig. 4E vs. F). Indeed, unlike WT FasL, when CHO cell purified His-tagged A274E FasL was analyzed in the cell culture medium of tested tumor cells, the latter was highly deficient in ligand trimer assembly on non-reducing and partly denaturing gels (Fig. 4G and Supplementary Fig. S5F). Similarly, the substitution of no side-chain amino acid G277 via short side-chain S277 next to highly hydrophobic residues (FFGLY- FFSLY) has the potential to cause steric hindrance in the beta-strand and to disrupt the trimer stability (Supplementary Fig. S5B vs. C), which was also evident in non-reducing gels (Fig. 4G and Supplementary Fig. S5F). Furthermore, when tested via ELISA, both His-tagged E247 and S277 FasL lost finding to Fas-IgG1 Fc (Fig. 3B), while R156G (a mutation in AA′ loop) maintained binding (Fig. 3B and Supplementary Fig. S5D vs. E). Strikingly opposite, despite a total loss of binding to Fas-IgG1 Fc (Fig. 3B), PPCR interacting FasL mutants (PPCR IFMs) E163A and E271A FasL trimerized similarly to WT FasL (Fig. 4G and Supplementary Fig. S5F). Regarding active receptor oligomerization, all mutants (E247, S277, A163, A271) that lost binding were defective in Fas clustering assays (Supplementary Fig. S3E).

**Fig. 5:**
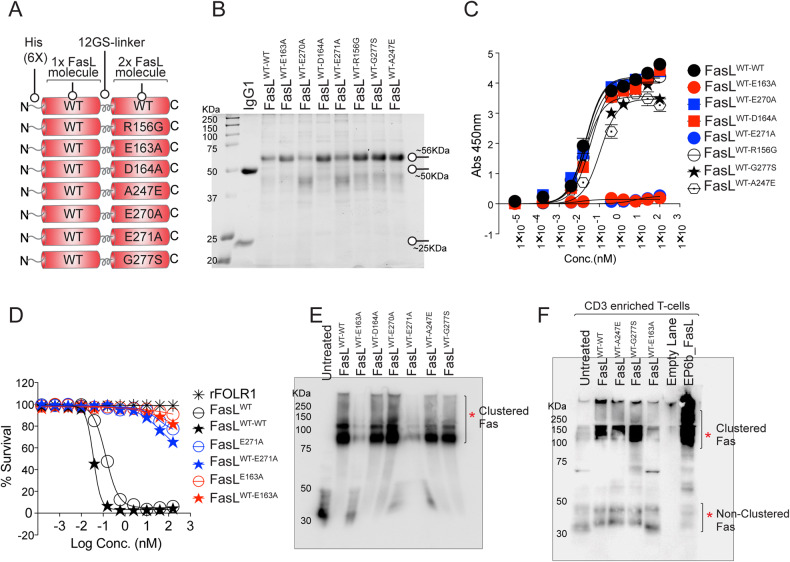
Haploinsufficiency of key ALPS mutants does not entirely block the FasL^WT^ function. **A** Schematic of genetic construction of various N-terminal His-tagged FasL mutants generated in tandem right next to FasL^WT^. **B** CHO cells expressing various tandem FasL mutants (as in **A**) were purified using HisTrap Ni Sepharose and were run on reducing gel along with an IgG1 for size confirmation. **C** Binding of indicated His-tagged tandem FasL mutants (as indicated on the right) to 96-well-immobilized Fc-tagged Fas by ELISA. **D** Cell survival assay of Jurkat cells after treatment with indicated His-tagged tandem FasL mutants. **E** Jurkat cells were treated with indicated His-tagged tandem FasL mutants on top for 1 hr, followed by low-dose BS3 treatment on ice (15 min) to capture the membrane-clustered Fas. Lysates were run in a non-reducing and partially denaturing gel, followed by Fas immunoblotting. **F** CD3-enriched human PBMC-derived T cells were treated with indicated His-tagged tandem FasL mutants followed by Fas clustering as described in (**E**).

A recent study has shown that, unlike homozygous mutations, patients with described heterozygous ALPS mutations maintained intact FasL cytotoxicity and were resistant to haploinsufficiency [29]. Considering that the FasL^E247^ and FasL^S277^ mutants did not effectively interact with Fas (Fig. 3B) and did not trimerize with self (Fig. 4G), we next asked if the latter would negatively impact Fas activation homologously but not heterologously. We also asked if Fas PPCR IFMs with the ability to trimerize normally will negatively affect the cytotoxic function of FasL^WT^ heterologously. As described, 1:1 DNA co-transfection conditions of WT and mutant FasL could generate technical variations [29]; we genetically ligated His-tagged WT-FasL with mutants in tandem via G4S linkers (two ligand molecules next to each other as two separate units) and expressed using a CHO expression system (Fig. 5A, B). To confirm that the haploinsufficiency of described ALPS mutant does not interfere with FasL^WT^ function, we tested tandemly purified FasL^WT-A247E^ and FasL^WT-G277S^ next to FasL^WT-WT^ (Fig. 5A). Despite significantly losing binding to Fas due to single FasL^E247^ and FasL^S277^ mutations (Fig. 3B), tandemly purified proteins harboring one unit of FasL^WT^ ligand molecule and other of FasL^E247^ and FasL^S277^ with mutations-maintained bindings similar to FasL^WT^ (Fig. 5C). Notably, similar to previously described DNA co-transfection studies [29], both tandem molecules (FasL^WT-A247E^ and ^FasLWT-G277S^) were cytotoxic very similar to FasL^WT-WT^ in Jurkat cells and tumor cells (Fig. 4C, D). These results confirmed that the single unit of neither FasL^A247E^ nor FasL^G277S^ trimerized or interfered with the other unit of FasL^WT^ despite being directly next to each other. When similar cytotoxicity assays were performed with tandemly expressed FasL^WT-E163A^ and FasL^WT-E271^ proteins, complete inhibition of Fas binding, clustering, and activity was observed (Figs. 4D and 5D–F). Mutation in residues next to PPCR interacting FasL mutants (PPCR IFMs), such as FasL^WT-D164A^ and FasL^WT-E270A^, also behaved like FasL^WT-A247E^ and ^FasLWT-G277S^ ALPS mutants (Figs. 4C, D and 5E). These results strongly indicate the dominant negative Fas inhibitory function of PPCR interacting FasL mutants. In Fas clustering assays, FasL^WT-G277S^ and FasL^WT-A247E^ showed complete Fas clustering, while FasL^WT-E163A^ and FasL^WT-E271^ proteins mutants showed no Fas clustering similar to single PPCR mutants (Fig. 5E, F).

### Limited activity of preclinical Fas antibodies is due to their inability to engage Fas PPCR

**Fig. 6:**
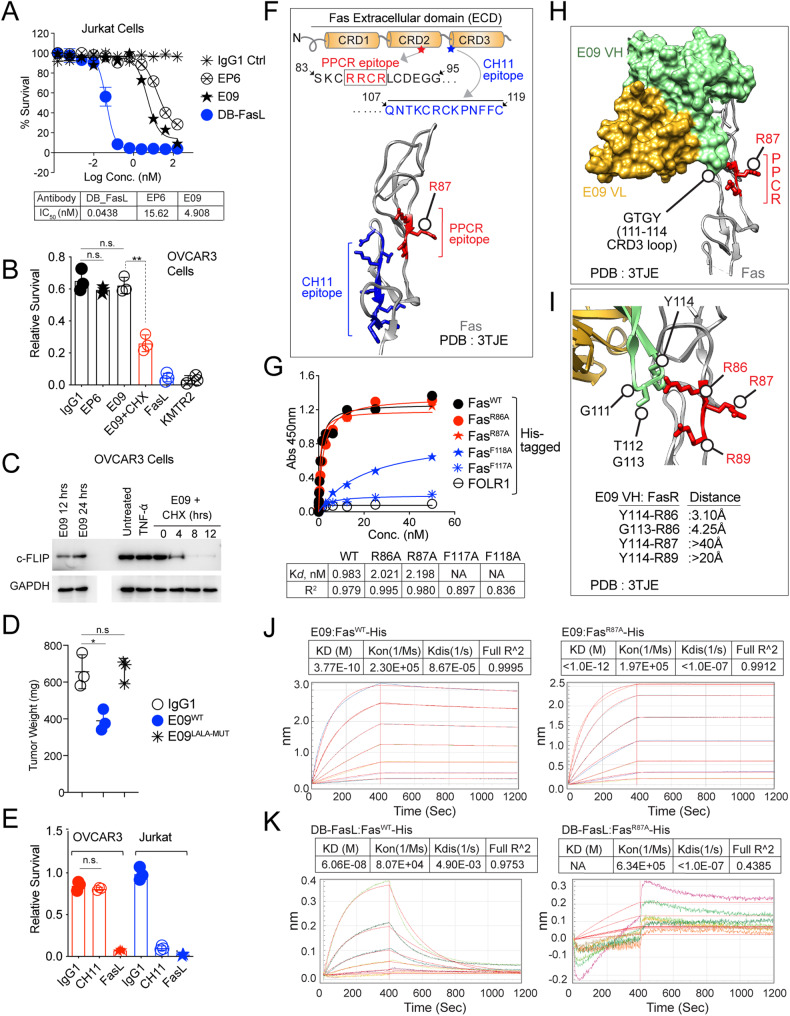
Preclinical Fas agonist antibodies are ineffective in tumor cells and do not engage Fas PPCR. **A** Jurkat cell killing assay of indicated Fas antibodies and dabigatran Fc-conjugated FasL, DB-FasL (see Supplementary Fig. S4A and Fig. 7 for DB-FasL design) (representative of 3). **B** Cell survival assay of OVCAR3 cells treated with indicated Fas agonist ± cFLIP inhibitor. DR5 agonist KMTR2 and FasL are positive control (*n* = 3). **C** Immunoblotting analysis of total cFLIP in the presence of cycloheximide ± E09 IgG1 and other controls. **D** 1 × 10^6^–2 × 10^6^ OVCAR3 tumor cells were injected subcutaneously in Athymic Nude animals with Matrigel in PBS. When tumors appeared on animals (3–4 weeks), animals were i.p. injected with indicated Fas agonists (4–6 doses, 50 mg), followed by tumor extraction and weight measurements. The same E09 IgG1 antibody was tested ± ADCC effector function (L234, L235 CH2 or L234A, L235A CH2). **E** Cell Survival assay of OVCAR3 and Jurkat cells treated CH11 IgM antibody, and FasL (*n* = 3). As a negative control, instead of IgM, IgG1 available in our lab was used. **F** Top: schematic of Fas ECD showing CH11 antibody epitope location in the context of PPCR. Bottom: ribbon depiction of CH11 antibody epitope (blue sticks) and PPCR (red sticks). **G** Binding of increasing concentrations of CH11 IgG1 against His-tagged indicated Fas PPCR mutants and F117A and F118A (CH11 epitope) mutants in a 96-well-immobilized ELISA assay. **H** A surface model interface of E09-Fas antibody against Fas PPCR (PDB:3TJE). VH (green) and VL (gold) are shown as space-filling models, while Fas (Gray) is in a ribbon structure. PPCR residues are shown as red sticks. **I** Same as (**H**) except for the E09 VH CDR3 loop with GTGY residues (green) and PPCR (red sticks) is focused with shown atomic distances. **J** The binding kinetics of immobilized His-tagged Fas^WT^ and Fas^R87A^ against the E09 antibody was measured using biolayer interferometry (BLI). **K** The binding kinetics of immobilized His-tagged Fas^WT^ and Fas^R87A^ against DB-FasL (see Supplementary Fig. S4A and Fig. 7 for DB-FasL design) were measured using BLI. Error bars in (**A**), (**B**), (**D**), and (**E**) represent SEM (*n* = 3).

Next, we tested the activity of previously described preclinical Fas agonist antibodies [19] E09 and EP6-1B (labeled EP6 hereafter) in the context of Fas PPCR. In concert with published literature, both antibodies showed cytotoxicity to Jurkat cells, however significantly lower than FasL (Fig. 6A). Importantly, these antibodies remained untested against tumor cells till today. Surprisingly, both antibodies were ineffective against various tumor cell lines in vitro (Fig. 6B, C) and in mouse xenograft studies (Fig. 6D). E09 did not inhibit the growth of grafted tumors unless engineered with ADCC effector function in its Fc (Fig. 6D), confirming the limited direct Fas agonist signaling activation function of E09 antibody against Fas in tumor cell membrane compared to Jurkat cell membrane.

To understand the differential cytotoxicity outcome, we tested the overall expression of DISC components in Jurkat vs. OVCAR3 cells. Despite similar Fas and caspase-8 expression, we observed little higher general expression of cFLIP and lower expression of RIP1 in tumor cells compared to Jurkat cells (Supplementary Fig. S6A). As cytotoxic agents are known to inhibit cFLIP expression and its lipid raft localization [33] (the critical site shown for effective Fas DISC assembly in T cells), we treated the tumor cells with cycloheximide. Indeed, the forced downregulation of cFLIP by cycloheximide decreased cFLIP levels and partly sensitized tumor cells to E09 (Fig. 6B, C). Considering that FasL is highly effective against tumor cells despite high cFLIP levels, these results suggested differential engagement of the Fas receptor (resulting in the differential apoptosis threshold) by FasL vs. E09. Next, we tested another Fas agonist CH11 antibody. Similar to E09 (IgG1), CH11 (IgM, Sigma) was cytotoxic to Jurkat cells but not to OVCAR3 tumor cells (Fig. 6E). Notably, the CH11 antibody epitope (^107^QNTKCRCKPNFFC^119^) does not engage the PPCR region of Fas [34], which we also confirmed (Fig. 6F, G). Hence, we hypothesize that E09 and CH11 are ineffective against tumor cells (and limitedly effective against Jurkat cells, Fig. 6A) because they are unable to engage the critical PPCR epitope. Although published structure reports of E09 have indicated potential interfacial contact between VH CDR3 of E09 above or near PPCR (Fig. 6H); however, the latter has not been tested experimentally. Indeed, when the published structure was closely analyzed, it was evident that the guanidinium side-chain interface of crucial PPCR residue (R87) is in opposite orientation to the CRD3 of E09 VH as well as of CH11 epitope (Figs. 2D vs. 6F, I). Although R86 of Fas forms interface contacts of <5 Å with glycine (G113) and tyrosine (Y114) of CRD3 VH (Fig. 6I), our analysis (Fig. 2D) showed that R86 is not the most critical to PPCR function and FasL activation. Indeed, when R86A, R87A, and R89A mutants were tested for binding, strikingly, none of the mutations interfered with the lead Fas agonist E09 binding (Fig. 6J, K and Supplementary Fig. S6B, C). To further confirm the latter, we mutated the E09 VH CDR3 loop interface near R86 from GTGY (111–114) to GGGG, TTTT, AAAA, and SSSS (Supplementary Fig. S6D). All four mutations did not affect the expression and binding of E09 to Fas (data not shown). Notably, E09 with GGGG, TTTT, AAAA, and SSSS mutations in CDR3 was as effective as WT E09 in killing Jurkat cells but ineffective against tumor cells indicating CDR3 loop is not significant in function (Supplementary Fig. S6E).

**Fig. 7:**
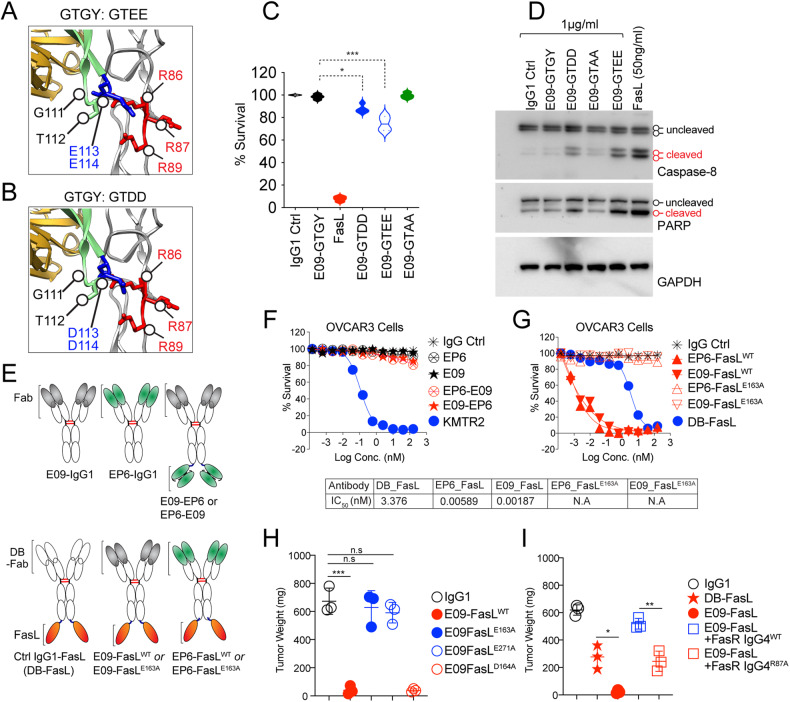
Negative charge substitution in CRD3 of E09 VH partly restores its Fas agonist activity in tumor cells. **A**, **B** The zoomed-in structure of Fas ECD region in complex with E09 IgG1. The interface of the E09 Fab against Fas PPCR is highlighted (PDB:3TJE). VH (green), VL (gold), and Fas (gray) are as depicted as ribbons. WT E09 contains GTGY (green sticks) in the CDR3 loop (see Supplementary Fig. 6I). GTGY residues of the CRD3 loop were mutated to GTEE or GTDD at 111–114 positions for the experiments described in (**C**) and (**D**). **C** 0.5 µg/ml WT E09 IgG1 (GTGY) or E09 IgG1 (GTEE or GTDD or GTAA) at 111–114 positions of E09 VH CRD3 along with other controls were added onto OVCAR3 in cell survival analysis post 36 h (*n* = 3+). **D** Same as (**C**), except indicated molecules (FasL at 20-fold lower concentration) were added for 10 hrs, followed by immunoblotting for caspase-8 and PARP. **E** Schematic showing genetic construction of generation of Fas agonist (E09, EP6) bispecific antibodies and antibody ligand bispecific combinations. **F** A 96-well plate OVCAR3 cell killing assay of indicated E09, EP6 Fas agonist monospecific antibodies, and E09-EP6 combination bispecific antibodies. KMTR2 was used as a positive control of killing. **G** Same as (**F**), except E09-FasL and EP6-FasL bispecific combinations were used with FasL^WT^ or FasL^E163A^. IC_50_ values are shown (*n* = 3). **H** 1 × 10^6^–1.5 × 10^6^ OVCAR3 tumor cells were injected subcutaneously in *NOD*.*Cg*-*Prkdc*^*scid*^Il2rg^tm1Wjl^/*SzJ* animals with Matrigel in PBS. When tumors appeared on animals (3–4 weeks), animals were i.p. injected with indicated E09-FasL bispecific combinations either with FasL^WT^ or FasL^E163A^ or FasL^D164A^ or FasL^E271A^ (*n* = 3+). **I** Same as (**H**), except 4-fold recombinant Fas-IgG4^WT^ and Fas-IgG4^R87A^ proteins were co-injected in indicated cases along with E09-FasL (*n* = 3+). Error bars in (**H**) and (**I**) represent SEM (*n* = 3+).

Based on structure analysis, Fas R87 engagement by FasL remains the most critical for cytotoxic activation in tumor cells (Figs. 2, and 3). However, considering its guanidinium side chain, which serves in proteins to interact with negatively charged groups, is in opposite orientation in antibody-receptor complex (E09-Fas, >40Å, Fig. 6I), we next asked if grafting of salt-bridge forming negatively charged residues against R86 (having guanidinium side-chain interface toward CRD3 of VH), could potentially reflect on PPCR engagement for optimal agonism by next generation of Fas antibodies. To this end, ^113^glycine and ^114^tyrosine (in GTGY) were substitutes with negatively charged (D and E) residues (Fig. 7A, B). Although the charged substitution resulted in significant loss of antibody expression (Supplementary Fig. S7A, B), we consistently observed partial gain in cytotoxicity in terms of cell death and activation of caspase-8 signaling by charged substitution, especially GTEE version of antibody (Fig. 7C, D). Considering that charge substitution without affinity maturation in GTEE could interfere with overall antibody structure, to further confirm the critical importance of PPCR engagement (especially R87) for effective cytotoxicity, we engineered E09 and EP6 with FasL as scFv (EP6-FasL and E09-FasL, see Table 2) (Fig. 7E and Supplementary Fig. S7A–C). As a measure of random Fas clustering without PPCR engagement, we also generated E09 and EP6 as bispecific antibodies in both orientations (Fig. 7E and Supplementary Fig. S7A–C). However, once again, although effective in the Jurkat cell, E09-EP6 bispecific antibodies were ineffective against OVCAR3 and other ovarian cancer cells (Fig. 7F). On the contrary, both EP6-FasL and E09-FasL were supremely effective (>200-fold decrease in IC_50_ values vs. DB-FasL) in OVCAR3 and Hey-A8 and many other tumor cells (Fig. 7G). Furthermore, E09-FasL with CDR3 VH residues GTGY substituted with GGGG, TTTT, AAAA, and SSSS was as effective as WT E09-FasL (Supplementary Fig. S7D). Strikingly, a single-point mutation in FasL R163A (or E271A) with the inability to form salt-bridges with R87 of Fas rendered both E09-Fasl and EP6-FasL ineffective in tumor cells and tumor xenografts (Fig. 7G–I). These results strongly support the critical need for preferentially engaging Fas^R87^ in PPCR to activate the effective cytotoxicity of tumor cells.

**Table 2:** Sequences of recombinant Fas agonist antibodies: monospecific IgG1, bispecific IgG1, and antibody-FasL IgG1 bispecific combinations.

Recombinant antibodies monospecific, bispecific and Fc-conjugated FasL	Sequences
E09 VH	QLQLQESGPGLVKPSETLSLTCTVSGASISANSYYGVWVRQSPGKGLEWVGSIAYRGNSNSGSTYYNPSLKSRATVSVDTSKNQVSLRLTSVTAADTALYYCARRQLLDDGTGYQWAAFDVWGQGTMVTVSSASTKGPSVFPLAPSSKSTSGGTAALGCLVKDYFPEPVTVSWNSGALTSGHTFPAVLQSSGLYSLSSVVTVPSSSLGTQTYICNVNHKPSNTKVDKKVEPKSCDKTHTCPPCPAPELLGGPSVFLFPPKPKDTLMISRTPEVTCVVVDVEHEDPEVKFNWYVDGVEVHNAKTKPREEQYNSTYRVVSVLTVLHQDWLNGKEYKCKVSNKALPAPIEKTISKAKGQPREPQVYTLPPSREEMTKNQVSLTCLVKGFYPSDIAVEWESNGQPENNYKTTPPVLDSDGSFFLYSKLTVDKSRWQQGNVFSCSVMHEALHNHYTQKSLSLSLGK
E09 VL	QSVLTQPPSVSEAPRQTVTISCSGNSFNIGRYPVNWYQQLPGKAPKLLIYYNNLRFSGVSDRFSGSKSGTSASLAIRDLLSEDEADYYCSTWDDTLKGWVFGGGTKVTVLGQPKAAPSVTLFPPSSEELQANKATLVCLISDFYPGAVTVAWKADSSPVKAGVETTTPSKQSNNKYAASSYLSLTPEQWKSHRSYSCQVTHEGSTVEKTVAPTECS
EP6B VH	QLQLQESGPGLVKPSETLSLTCTVSGASISANSYYGVWVRQSPGKGLEWVGSIAYRGNSNSGSTYYNPSLKSRATVSVDSSKNQVSLRLTSVTAADTALYYCARRQLLDDGTGYQWAAFDVWGQGTMVTVSSASTKGPSVFPLAPSSKSTSGGTAALGCLVKDYFPEPVTVSWNSGALTSGVHTFPAVLQSSGLYSLSSVVTVPSSSLGTQTYICNVNHKPSNTKVDKKVEPKSCDKTHTCPPCPAPELLGGPSVFLFPPKPKDTLMISRTPEVTCVVVDVEHEDPEVKFNWYVDGVEVHNAKTKPREEQYNSTYRVVSVLTVLHQDWLNGKEYKCKVSNKALPAPIEKTISKAKGQPREPQVYTLPPSREEMTKNQVSLTCLVKGFYPSDIAVEWESNGQPENNYKTTPPVLDSDGSFFLYSKLTVDKSRWQQGNVFSCSVMHEALHNHYTQKSLSLSLGK
EP6B VL	QSVLTQPPSVSEAPRQTVTISCSGNSSNIGRYPVNWYQQLPGKAPKLLIYSDNLRFSGVPDRFSGSKSGTTASLAIRDLLSEDEADYYCSTWDDTLEGWVFGGGTKVTVLGQPKAAPSVTLFPPSSEELQANKATLVCLISDFYPGAVTVAWKADSSPVKAGVETTTPSKQSNNKYAASSYLSLTPEQWKSHRSYSCQVTHEGSTVEKTVAPTECS
E09 VH_CDR3 GTGT_GGGG	QLQLQESGPGLVKPSETLSLTCTVSGASISANSYYGVWVRQSPGKGLEWVGSIAYRGNSNSGSTYYNPSLKSRATVSVDTSKNQVSLRLTSVTAADTALYYCARRQLLDD**GGGG**QWAAFDVWGQGTMVTVSSASTKGPSVFPLAPSSKSTSGGTAALGCLVKDYFPEPVTVSWNSGALTSGVHTFPAVLQSSGLYSLSSVVTVPSSSLGTQTYICNVNHKPSNTKVDKKVEPKSCDKTHTCPPCPAPELLGGPSVFLFPPKPKDTLMISRTPEVTCVVVDVEHEDPEVKFNWYVDGVEVHNAKTKPREEQYNSTYRVVSVLTVLHQDWLNGKEYKCKVSNKALPAPIEKTISKAKGQPREPQVYTLPPSREEMTKNQVSLTCLVKGFYPSDIAVEWESNGQPENNYKTTPPVLDSDGSFFLYSKLTVDKSRWQQGNVFSCSVMHEALHNHYTQKSLSLSLGK
E09 VH_CDR3 GTGT_SSSS	QLQLQESGPGLVKPSETLSLTCTVSGASISANSYYGVWVRQSPGKGLEWVGSIAYRGNSNSGSTYYNPSLKSRATVSVDTSKNQVSLRLTSVTAADTALYYCARRQLLDD**SSSS**QWAAFDVWGQGTMVTVSSASTKGPSVFPLAPSSKSTSGGTAALGCLVKDYFPEPVTVSWNSGALTSGVHTFPAVLQSSGLYSLSSVVTVPSSSLGTQTYICNVNHKPSNTKVDKKVEPKSCDKTHTCPPCPAPELLGGPSVFLFPPKPKDTLMISRTPEVTCVVVDVEHEDPEVKFNWYVDGVEVHNAKTKPREEQYNSTYRVVSVLTVLHQDWLNGKEYKCKVSNKALPAPIEKTISKAKGQPREPQVYTLPPSREEMTKNQVSLTCLVKGFYPSDIAVEWESNGQPENNYKTTPPVLDSDGSFFLYSKLTVDKSRWQQGNVFSCSVMHEALHNHYTQKSLSLSLGK
E09 VH_CDR3 GTGT_AAAA	QLQLQESGPGLVKPSETLSLTCTVSGASISANSYYGVWVRQSPGKGLEWVGSIAYRGNSNSGSTYYNPSLKSRATVSVDTSKNQVSLRLTSVTAADTALYYCARRQLLDD**AAAA**QWAAFDVWGQGTMVTVSSASTKGPSVFPLAPSSKSTSGGTAALGCLVKDYFPEPVTVSWNSGALTSGVHTFPAVLQSSGLYSLSSVVTVPSSSLGTQTYICNVNHKPSNTKVDKKVEPKSCDKTHTCPPCPAPELLGGPSVFLFPPKPKDTLMISRTPEVTCVVVDVEHEDPEVKFNWYVDGVEVHNAKTKPREEQYNSTYRVVSVLTVLHQDWLNGKEYKCKVSNKALPAPIEKTISKAKGQPREPQVYTLPPSREEMTKNQVSLTCLVKGFYPSDIAVEWESNGQPENNYKTTPPVLDSDGSFFLYSKLTVDKSRWQQGNVFSCSVMHEALHNHYTQKSLSLSLGK
E09-FasL^WT^	QLQLQESGPGLVKPSETLSLTCTVSGASISANSYYGVWVRQSPGKGLEWVGSIAYRGNSNSGSTYYNPSLKSRATVSVDTSKNQVSLRLTSVTAADTALYYCARRQLLDDGTGYQWAAFDVWGQGTMVTVSSASTKGPSVFPLAPSSKSTSGGTAALGCLVKDYFPEPVTVSWNSGALTSGVHTFPAVLQSSGLYSLSSVVTVPSSSLGTQTYICNVNHKPSNTKVDKKVEPKSCDKTHTCPPCPAPELLGGPSVFLFPPKPKDTLMISRTPEVTCVVVDVEHEDPEVKFNWYVDGVEVHNAKTKPREEQYNSTYRVVSVLTVLHQDWLNGKEYKCKVSNKALPAPIEKTISKAKGQPREPQVYTLPPSREEMTKNQVSLTCLVKGFYPSDIAVEWESNGQPENNYKTTPPVLDSDGSFFLYSKLTVDKSRWQQGNVFSCSVMHEALHNHYTQKSLSLSLGKGGSGGSPSPPPEKKELRKVAHLTGKSNSRSMPLEWEDTYGIVLLSGVKYKKGGLVINETGLYFVYSKVYFRGQSCNNLPLSHKVYMRNSKYPQDLVMMEGKMMSYCTTGQMWARSSYLGAVFNLTSADHLYVNVSELSLVNFEESQTFFGLYKL
E09-FasL^E163A^	QLQLQESGPGLVKPSETLSLTCTVSGASISANSYYGVWVRQSPGKGLEWVGSIAYRGNSNSGSTYYNPSLKSRATVSVDTSKNQVSLRLTSVTAADTALYYCARRQLLDDGTGYQWAAFDVWGQGTMVTVSSASTKGPSVFPLAPSSKSTSGGTAALGCLVKDYFPEPVTVSWNSGALTSGVHTFPAVLQSSGLYSLSSVVTVPSSSLGTQTYICNVNHKPSNTKVDKKVEPKSCDKTHTCPPCPAPELLGGPSVFLFPPKPKDTLMISRTPEVTCVVVDVEHEDPEVKFNWYVDGVEVHNAKTKPREEQYNSTYRVVSVLTVLHQDWLNGKEYKCKVSNKALPAPIEKTISKAKGQPREPQVYTLPPSREEMTKNQVSLTCLVKGFYPSDIAVEWESNGQPENNYKTTPPVLDSDGSFFLYSKLTVDKSRWQQGNVFSCSVMHEALHNHYTQKSLSLSLGKGGSGGSPSPPPEKKELRKVAHLTGKSNSRSMPLEW**A**DTYGIVLLSGVKYKKGGLVINETGLYFVYSKVYFRGQSCNNLPLSHKVYMRNSKYPQDLVMMEGKMMSYCTTGQMWARSSYLGAVFNLTSADHLYVNVSELSLVNFEESQTFFGLYKL
E09-FasL^E163A^	QLQLQESGPGLVKPSETLSLTCTVSGASISANSYYGVWVRQSPGKGLEWVGSIAYRGNSNSGSTYYNPSLKSRATVSVDTSKNQVSLRLTSVTAADTALYYCARRQLLDDGTGYQWAAFDVWGQGTMVTVSSASTKGPSVFPLAPSSKSTSGGTAALGCLVKDYFPEPVTVSWNSGALTSGVHTFPAVLQSSGLYSLSSVVTVPSSSLGTQTYICNVNHKPSNTKVDKKVEPKSCDKTHTCPPCPAPELLGGPSVFLFPPKPKDTLMISRTPEVTCVVVDVEHEDPEVKFNWYVDGVEVHNAKTKPREEQYNSTYRVVSVLTVLHQDWLNGKEYKCKVSNKALPAPIEKTISKAKGQPREPQVYTLPPSREEMTKNQVSLTCLVKGFYPSDIAVEWESNGQPENNYKTTPPVLDSDGSFFLYSKLTVDKSRWQQGNVFSCSVMHEALHNHYTQKSLSLSLGKGGSGGSPSPPPEKKELRKVAHLTGKSNSRSMPLEWE**A**TYGIVLLSGVKYKKGGLVINETGLYFVYSKVYFRGQSCNNLPLSHKVYMRNSKYPQDLVMMEGKMMSYCTTGQMWARSSYLGAVFNLTSADHLYVNVSELSLVNFEESQTFFGLYKL
E09-FasL^E270A^	QLQLQESGPGLVKPSETLSLTCTVSGASISANSYYGVWVRQSPGKGLEWVGSIAYRGNSNSGSTYYNPSLKSRATVSVDTSKNQVSLRLTSVTAADTALYYCARRQLLDDGTGYQWAAFDVWGQGTMVTVSSASTKGPSVFPLAPSSKSTSGGTAALGCLVKDYFPEPVTVSWNSGALTSGVHTFPAVLQSSGLYSLSSVVTVPSSSLGTQTYICNVNHKPSNTKVDKKVEPKSCDKTHTCPPCPAPELLGGPSVFLFPPKPKDTLMISRTPEVTCVVVDVEHEDPEVKFNWYVDGVEVHNAKTKPREEQYNSTYRVVSVLTVLHQDWLNGKEYKCKVSNKALPAPIEKTISKAKGQPREPQVYTLPPSREEMTKNQVSLTCLVKGFYPSDIAVEWESNGQPENNYKTTPPVLDSDGSFFLYSKLTVDKSRWQQGNVFSCSVMHEALHNHYTQKSLSLSLGKGGSGGSPSPPPEKKELRKVAHLTGKSNSRSMPLEWEDTYGIVLLSGVKYKKGGLVINETGLYFVYSKVYFRGQSCNNLPLSHKVYMRNSKYPQDLVMMEGKMMSYCTTGQMWARSSYLGAVFNLTSADHLYVNVSELSLVNF**A**ESQTFFGLYKL
E09-FasL^E271A^	QLQLQESGPGLVKPSETLSLTCTVSGASISANSYYGVWVRQSPGKGLEWVGSIAYRGNSNSGSTYYNPSLKSRATVSVDTSKNQVSLRLTSVTAADTALYYCARRQLLDDGTGYQWAAFDVWGQGTMVTVSSASTKGPSVFPLAPSSKSTSGGTAALGCLVKDYFPEPVTVSWNSGALTSGVHTFPAVLQSSGLYSLSSVVTVPSSSLGTQTYICNVNHKPSNTKVDKKVEPKSCDKTHTCPPCPAPELLGGPSVFLFPPKPKDLMISRTPEVTCVVVDVEHEDPEVKFNWYVDGVEVHNAKTKPREEQYNSTYRVVSVLTVLHQDWLNGKEYKCKVSNKALPAPIEKTISKAKGQPREPQVYTLPPSREEMTKNQVSLTCLVKGFYPSDIAVEWESNGQPENNYKTTPPVLDSDGSFFLYSKLTVDKSRWQQGNVFSCSVMHEALHNHYTQKSLSLSLGKGGSGGSPSPPPEKKELRKVAHLTGKSNSRSMPLEWEDTYGIVLLSGVKYKKGGLVINETGLYFVYSKVYFRGQSCNNLPLSHKVYMRNSKYPQDLVMMEGKMMSYCTTGQMWARSSYLGAVFNLTSADHLYVNVSELSLVNFE**A**SQTFFGLYKL
E09-EP6B bispecific	QLQLQESGPGLVKPSETLSLTCTVSGASISANSYYGVWVRQSPGKGLEWVGSIAYRGNSNSGSTYYNPSLKSRATVSVDTSKNQVSLRLTSVTAADTALYYCARRQLLDDGTGYQWAAFDVWGQGTMVTVSSASTKGPSVFPLAPSSKSTSGGTAALGCLVKDYFPEPVTVSWNSGALTSGVHTFPAVLQSSGLYSLSSVVTVPSSSLGTQTYICNVNHKPSNTKVDKKVEPKSCDKTHTCPPCPAPELLGGPSVFLFPPKPKDTLMISRTPEVTCVVVDVEHEDPEVKFNWYVDGVEVHNAKTKPREEQYNSTYRVVSVLTVLHQDWLNGKEYKCKVSNKALPAPIEKTISKAKGQPREPQVYTLPPSREEMTKNQVSLTCLVKGFYPSDIAVEWESNGQPENNYKTTPPVLDSDGSFFLYSKLTVDKSRWQQGNVFSCSVMHEALHNHYTQKSLSLSLGKGGSGGSQSVLTQPPSVSEAPRQTVTISCSGNSSNIGRYPVNWYQQLPGKAPKLLIYSDNLRFSGVPDRFSGSKSGTTASLAIRDLLSEDEADYYCSTWDDTLEGWVFGGGTKVTVLGGGGSGGGDSGGGGSGGGGSQLQLQESGPGLVKPSETLSLTCTVSGASISANSYYGVWVRQSPGKGLEWVGSIAYRGNSNSGSTYYNPSLKSRATVSVDSSKNQVSLRLTSVTAADTALYYCARRQLLDDGTGYQWAAFDVWGQGTMVTVSSGGSGGSQSVLTQPPSVSEAPRQTVTISCSGNSSNIGRYPVNWYQQLPGKAPKLLIYSDNLRFSGVPDRFSGSKSGTTASLAIRDLLSEDEADYYCSTWDDTLEGWVFGGGTKVTVLGGGGSGGGDSGGGGSGGGGSQLQLQESGPGLVKPSETLSLTCTVSGASISANSYYGVWVRQSPGKGLEWVGSIAYRGNSNSGSTYYNPSLKSRATVSVDSSKNQVSLRLTSVTAADTALYYCARRQLLDDGTGYQWAAFDVWGQGTMVTVSS
E09 VH_CDR3 GTGT_GTDD	QLQLQESGPGLVKPSETLSLTCTVSGASISANSYYGVWVRQSPGKGLEWVGSIAYRGNSNSGSTYYNPSLKSRATVSVDTSKNQVSLRLTSVTAADTALYYCARRQLLDD**GTDD**QWAAFDVWGQGTMVTVSSASTKGPSVFPLAPSSKSTSGGTAALGCLVKDYFPEPVTVSWNSGALTSGVHTFPAVLQSSGLYSLSSVVTVPSSSLGTQTYICNVNHKPSNTKVDKKVEPKSCDKTHTCPPCPAPELLGGPSVFLFPPKPKDTLMISRTPEVTCVVVDVEHEDPEVKFNWYVDGVEVHNAKTKPREEQYNSTYRVVSVLTVLHQDWLNGKEYKCKVSNKALPAPIEKTISKAKGQPREPQVYTLPPSREEMTKNQVSLTCLVKGFYPSDIAVEWESNGQPENNYKTTPPVLDSDGSFFLYSKLTVDKSRWQQGNVFSCSVMHEALHNHYTQKSLSLSLGK
E09 VH_CDR3 GTGT_GTEE	QLQLQESGPGLVKPSETLSLTCTVSGASISANSYYGVWVRQSPGKGLEWVGSIAYRGNSNSGSTYYNPSLKSRATVSVDTSKNQVSLRLTSVTAADTALYYCARRQLLDD**GTEE**QWAAFDVWGQGTMVTVSSASTKGPSVFPLAPSSKSTSGGTAALGCLVKDYFPEPVTVSWNSGALTSGVHTFPAVLQSSGLYSLSSVVTVPSSSLGTQTYICNVNHKPSNTKVDKKVEPKSCDKTHTCPPCPAPELLGGPSVFLFPPKPKDTLMISRTPEVTCVVVDVEHEDPEVKFNWYVDGVEVHNAKTKPREEQYNSTYRVVSVLTVLHQDWLNGKEYKCKVSNKALPAPIEKTISKAKGQPREPQVYTLPPSREEMTKNQVSLTCLVKGFYPSDIAVEWESNGQPENNYKTTPPVLDSDGSFFLYSKLTVDKSRWQQGNVFSCSVMHEALHNHYTQKSLSLSLGK

### A single-point mutation in Fas PPCR R87 also abolishes bystander function of CAR-T

**Fig. 8:**
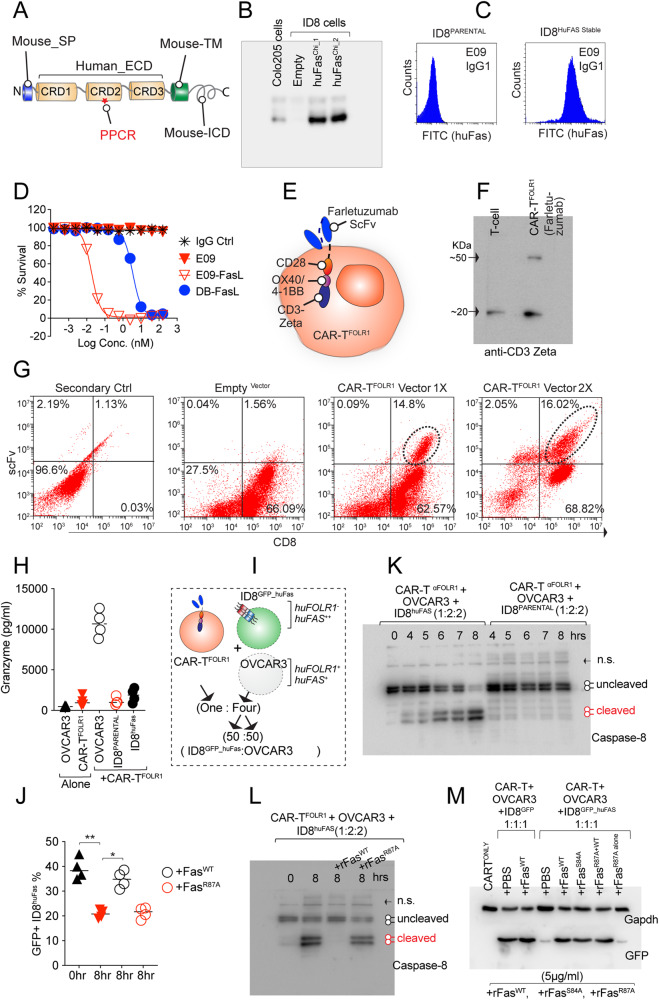
Fas PPCR R87 is critical for CAR-T bystander function. **A** Cartoon schematic of chimeric Fas with human ectodomain and murine TM, ICD, and signal peptide. **B** Immunoblotting confirmation of murine ID8 cell clones expressing huFas chimeric clones 1 and 2. **C** E09 IgG1 Flow cytometry confirmation of murine ID8 cells expressing huFas ectodomain. **D** Confirmation of cytotoxic sensitivity of chimeric huFas stable murine ID8 cells against Fas agonist reagents. **E** Cartoon schematic of anti-FOLR1 (Farletuzumab) expressing CAR-T (CAR-T^FOLR1^). **F** Immunoblotting confirmation of CAR-T^FOLR1^ construct expression in T cells using CD3 zeta antibody. **G** Flow cytometry using goat anti-human Fab specific antibody after two rounds of CAR-T construction lentiviral infections (represents *n* = 3). **H** CAR-T^FOLR1^ cells were co-cultured either alone or with OVCAR3 cells or ID8^Parental^ cells, or ID8^huFas^ stable cells at an effector/target (E/T) ratio 5:1 for 4 h followed by granzyme B measurement using ELISA (*n* = 4). **I** Schematic of CAR-T^FOLR1^ cells co-cultured with the 50:50 mix of GFP^+^ ID8^huFas^ stable cells and OVCAR3. In additional sets, cultured plates were precoated either with recombinant rFas^WT^ or rFas^R87A^ (5 μg/ml). **J** %GFP flow cytometry signal as an indicator of FOLR1 antigen negative but GFP positive ID8^huFas^ stable cell lysis from the experiment described in I (*n* = 3). **K** Same as (**J**) and (**I**), except in one additional set, a 50:50 mix of ID8^Parental^ (huFas^-^) cells and OVCAR3 were used for indicated times. Immunoblotting analyzed the total cell lysates for cleaved caspase-8 levels. **L** Same as (**K**), except additional controls were included via precoating the culture plates with rFas^WT^ or rFas^R87A^ (5 μg/ml) (*n* = 3). **M** Same as (**K**) and (**L**), except GFP levels (as an indicator of ID8^huFas^ stable cell lysis) were analyzed using immunoblotting. Error bars in (**J**) represent SEM (*n* = 3+).

Recently, it has been shown that scenarios where antigen loss or antigen mutation in highly heterogenous tumors interferes with the binding of scFv of a designated CAR-T, FasL-Fas-mediated off-target (bystander) killing of antigen-negative tumor cells is critical in human clinical trials as well as in murine studies [16]. To confirm if similar to Fas agonist antibodies (Figs. 6 and 7), intact PPCR function (especially R87 engagement in Fas) is key for CAR-T FasL-mediated bystander killing, we generated human Fas-expressing GFP stable heterogenous murine ID8 cells. As huFasL and Fas agonist E09/EP6 antibodies do not bind murine Fas, we engineered a chimeric Fas construct, with the ECD of human Fas fused with mouse Fas transmembrane and intracellular domain as described earlier for DR5 [35] (Fig. 8A). The chimeric^HuFas^ expressing cell lines bound human Fas agonists and were highly sensitive to human FasL and E09-FasL proteins (Fig. 8B–D). Next, we generated anti-FOLR1 scFv (farletuzumab) expressing human CAR-T cells (Fig. 8E). After two rounds of lentiviral infection, ~16% of CD8+ cells were found positively expressing anti-FOLR1 scFv (Fig. 8F, G). When tested in various ratios with high and medium FOLR1+ OVCAR3 and colo205 cells, CAR-T cells were highly effective in complete cell lysis even in 8:1 (OVCAR3:CAR-T) ratios (Supplementary Fig. S8A–D). Importantly the cell death was guided by antigen-targeting induced CAR-T activation as evident by granzyme B activity (~10,000 pg/ml) (Fig. 8H). At highly saturating blot exposure, a minimal FasL-mediated caspase-8 cleavage was evident in the coculture of CAR-T with OVCAR3 + ID8^PARETNAL^ cells (Supplementary Fig. S8E, F), suggesting a potential indication of Fas activation in an antigen-independent manner. As published earlier by us, farletuzumab scFv does not bind murine FOLR1 [26]; hence, the farletuzumab^CAR-T^ cells were totally ineffective in killing murine ID8 cells (data not shown). On the contrary, when a coculture of OVCAR3 cells with GFP stable chimeric^HuFas^ expressing murine ID8^HuFas^ cells was incubated with farletuzumab^CAR-T^ cells (2:2:1 ratio), significant cell death of ID8^HuFas+^ cells was observed (evident with a decrease in GFP signal) (Fig. 8I, J and Supplementary Fig. S9A, B). Since farletuzumab^CAR-T^ cells have no direct capability to engage murine FOLR1 in GFP stable ID8^HuFas^ cells, the decrease in GFP signal is contributed by huFas activation and bystander killing in antigen-negative (FOLR1^-^) murine ID8 cells. When measured by ELISA, the ID8^HuFas^: CAR-T (6:1) co-cultures showed only <500 pg/ml levels of granzyme B in media. In contrast, OVCAR3:CAR-T (6:1) showed around >10,000 pg/ml levels of granzyme B (Supplementary Fig. S9C), confirming the antigen-independent and Fas-dependent activation of ID8^HuFas^ cells by CAR-T. Notably, a highly cleaved caspase-8 was evident only in ID8^HuFas^ coculture but not in ID8^PARENTAL^ coculture (Fig. 8K). Furthermore, precoating and saturating the culture plates with recombinant Fas-IgG1-Fc (rFas^WT^) inhibited caspase-8 cleavage (Fig. 8L). On the contrary, a single R87A PPCR mutation (rFas^R87A^) rendered it ineffective (Fig. 8L). In line with caspase-8 activation-mediated cytotoxicity, loss of GFP was reversed by Fas^WT^ and Fas^S84A^ but not Fas^R87A^ (Fig. 8M).

**Fig. 9:**
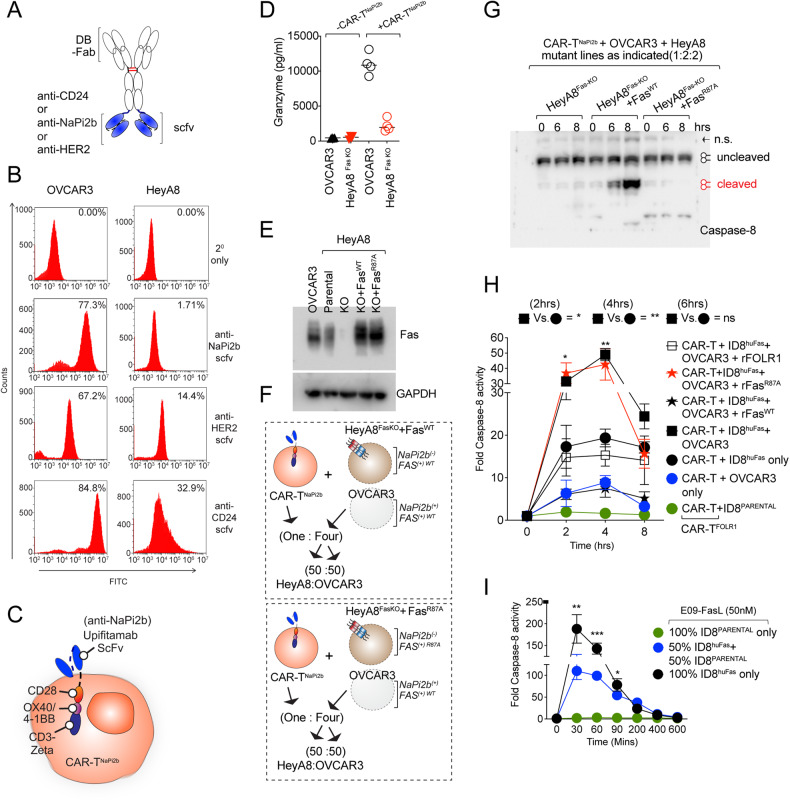
CAR-T-targeting antigen-positive cells next to antigen-negative cells in heterogenous cultures are crucial for optimal caspase-8 activation. **A** Schematic of anti-CD24, anti-NaPi2b, and anti-HER2 scFvs (in blue) conjugated to idarucizumab-Fc (DB-Fc) used for flow cytometry in (**B**). **B** Flow cytometry analysis of OVCAR3 and Hey-A8 cells using a list of scFv described in (**A**). **C** Cartoon schematic of anti-NaPi2b (Upifitamab) expressing CAR-T (CAR-T^NaPi2b^). **D** OVCAR3 cells or Hey-A8 FasKO cells were co-cultured either alone or with CAR-T^NaPi2b^ cells at 5:1 ratio for 4 h followed by granzyme B measurement using ELISA (*n* = 3+). **E** Immunoblotting confirmation Fas expression in Hey-A8^PARENTAL^, Hey-A8^FasKO^, and Hey-A8^FasKO^ transfected with Fas^WT^ and Fas^R87A^. OVCAR3 control lysates were used side-by-side. **F** Schematic of CAR-T^NaPi2b^ T cells co-cultured with the 50:50 mix of Hey-A8^FasKO^ overexpressing exogenous Fas^WT^ (top) or overexpressing exogenous Fas^R87A^ (bottom) and OVCAR3 cells. **G** Same as (**F**), except total lysates of indicated combinations were immunoblotted for caspase-8 cleavage. **H** In one set, CAR-T^FOLR1^ cells were co-cultured either with huFOLR1 antigen^+^ OVCAR3 alone (blue circle) or huFOLR1 antigen^-^ ID8^PARENTAL^ alone (green circle) or huFOLR1 antigen^-^ ID8^huFas^ stable (black circle) alone. In the second set, CAR-T^FOLR1^ cells were co-cultured with a 50:50 mix of huFOLR1 antigen^+^ OVCAR3+ huFOLR1 antigen^-^ ID8^huFas^ stable in the presence of indicated precoated recombinant proteins [rFas^WT^ or rFas^R87A^ or rFOLR1 (5 μg/ml)]. After 2, 4, and 8 hr, all sets were analyzed for caspase-8 assays. **I** E09-FasL bispecific antibody was added for indicated times either to the 50:50 mix of GFP+ ID8huFas stable and ID8 regular cells or 100% GFP+ ID8huFas stable alone cells followed by caspase-8 activity analysis. Error bars in (**H**) and (**I**) represent SD (*n* = 3+).

From a human therapeutic perspective, to reconfirm these findings of Fas R87 requirement in bystander killing by CAR-T cells, we tested multiple ovarian tumor cell lines to find a clinically important receptor antigen-negative line. Interestingly, when a clinical upifitamab [36] scFv (Fig. 9A) against a type-2 sodium-dependent phosphate transporter named *SLC34A2* (NaPi2b), known to be highly elevated in ovarian tumors [37] was tested, almost undetectable surface expression of NaPi2b on HeyA8 cells was observed as compared to OVCAR3 (Fig. 9B). In support, almost 90% reduced expression of NaPi2b was evident in Hey-A8 cells (compared to OVCAR3) when tested with commercial non-functional immunoblotting antibody (Supplementary Fig. S9D). Most other receptors, such as FOLR1, CD24, and HER2, etc., were expressed in both cell lines (Fig. 9B and Supplementary Fig. S9D). when tested, upifitamab scFv (anti-NaPi2b) expressing CAR-T cells were ineffective in killing and significantly reduced granzyme production of Hey-A8 cells vs. OVCAR3 cells (Fig. 9C, D, and data now shown) reaffirming inability to effectively engage Hey-A8 cells by upifitamab. Next, we made use of Fas knocked out Hey-A8 cells (kindly provided by Dr Marcus E. Peters, Northwestern University) to transiently overexpress either WT or R87A Fas under CMV promoter (Fig. 9E). When a coculture of OVCAR3 and Hey-A8^FasKO^ expressing either Fas^WT^ or Fas^R87A^ (Fig. 9F) were incubated with upifitamab^CAR-T^ cells (2:2:1 ratio), clear caspase-8 activation was evident in Hey-A8^FasKO^ expressing Fas^WT^ (Fig. 9G and Supplementary Fig. S9E). On the contrary, undetectable caspase-8 cleavage was evident in co-cultures expressing Fas^R87A^ (Fig. 9G and Supplementary Fig. S9E). These results strongly support the importance of PPCR epitope (especially R87) engagement for effective antigen-independent bystander Fas activity of CAR-T-cells expressing scFv against clinically important targets [38, 39]. Notably, the Fas signaling and caspase-8 activation in antigen-negative (huFOLR1 Ag^-^, huFas^+^: ID8^HuFas^) cells were significantly enhanced in the presence of target antigen-expressing (huFOLR1 Ag^+^, huFas^+^: OVCAR3) cells (Fig. 9H), potentially due to high-affinity CAR-T binding to target antigen enhancing the avidity of CAR-T membrane FasL binding to huFas-expressing ID8 (ID8^HuFas^) cells (Fig. 9H). Unlike targeting antigen-directed CAR-T bystander function of Fas activation, E09-FasL-directed Fas activation (in terms of caspase-8 activation) was directly proportional to Fas expression in tumor cells (Fig. 9I and Supplementary Fig. S9F, G). Collectively, the data strongly indicate the importance of PPCR engagement for effective Fas agonist signaling by antibodies and CAR-T cells.

## Discussion

Due to its regulatory role in T-cell homeostasis, loss of function in ALPS, and pro-oncogenic signaling, FasL/Fas signaling remains a key area of investigation [6, 7, 18]. More importantly, the bystander function of Fas signaling in CAR-T efficacy further underscores its significance in reviving the field of cancer immunotherapy and the success of chimeric antigen receptor-based cell therapies [16, 17]. By making use of various recombinant FasL proteins alone or in tandem, Fas agonist monospecific antibodies, and dual-specificity strategies, we present here extensive biochemical evidence of a highly undervalued Fas-directed tumor cytotoxicity mechanism with a great potential to enhance Ag-mediated cytotoxicity of CAR-T-based cell therapies and general immunotherapies. Even more important is the characterization of crucial PPCR residue (R87) in Fas CRD2, engaging of which being highly critical for the efficacy of Fas agonist strategies and CAR-T cell-based immunotherapies. Mutational substitution of the essential PPCR residue R87 abolished the cytotoxic function of FasL alone and in a bispecific combination with Fas agonist antibodies.

Numerous studies in APLS patients carrying germline mutations (A247E, G277S, R156G, C202S, Y166C, Q130E, S155L, L181P, etc.) in the *FASLG* gene have been described [29, 31]. However, none of the ALPS patient cases has shown to have germline homozygous or heterozygous mutations in the E163 and E271 of *FASLG*. Based on our His-tagged FasL and tandem FasL studies, it is evident that both E163 and E271 are highly dominant mutations that totally abolish the Fas cytotoxic signaling. Considering that tested A247E, G277S, and R156G APLS mutations in our assays would still be able to have some degree of cytotoxicity at the higher dose (ranging from 10–50%), likely, total abolishment of Fas signaling due to genetic mutations in E163 and E271 could be lethal in early development. The latter potentially has rendered the discovery of E163 and E271 mutations impervious in ALPS patients.

Our tandem WT-E163A and WT-E271A mutations suggest that heteromerization of these PPCR interface FasL mutants (PPCR IFMs) with wildtype FasL abolishes FasL function completely. On the contrary, tandem molecules generated with WT-A247E, WT-G277S mutations, maintained activity similarly to wildtype ligands. The latter indicates potentially a differential FasL conformation with E163A and E271A mutations, leading to a completely inactive molecule. Thus, mutations in crucial PPCR-engaging FasL residues maintain the mutant ligands’ capability to bind and saturate the wildtype FasL, rendering it unable to access the Fas receptor. On the other hand, potentially, FasL is conformed differently with A247E and G277S ALPS mutations. In support of previously published studies [29] and our ligand trimerization data suggest that A247E (and G277S, etc.) lacks trimerization ability with self and wildtype FasL. Hence, neither A247E nor G277S saturates the wildtype FasL function. Altogether, in agreement with previously published comprehensive analysis, haploinsufficiency in described mutations (G277S, A274E tested by us and others [29]) is insufficient to block Fas function and causes ALPS disease ultimately.

Despite activating a common extrinsic apoptotic signaling pathway, Fas shares uncommon interacting partners at the cell surface of T cells compared to cancer cells [6]. For example, the TCR-CD3 complex is a direct regulatory partner of Fas in T cells [40]. Considering that cancer cells neither express TCR complex nor CD3, differentially expressed tumor-enriched surface receptors in highly dynamic, detergent resistant, and cholesterol plus glycosphingolipids enriched membrane domains regulate Fas function differently. Indeed, compared to healthy cells and T cells, transformed solid cancer cells are known to have an increased distribution of negatively charged phospholipids such as phosphatidylserine and phosphoproteins on the extracellular side of the membrane [41, 42] due to cancer hallmark loss of cell polarity. In the latter case, likely, higher electrostatic interaction of positively charged PPCR region of Fas (centered around R87) with the differentially charged cancer cell membrane (but not in T cells or potentially Jurkat cells) phospholipids or unknown proteins are involved in differentially regulating DISC signaling [11]. In support of the latter, previous studies have described highly differential FasL-independent distribution of Fas in non-raft compartments of Jurkat cells and T cells compared to tumor cells [43]. Considering that we consistently observed a clear difference in cytotoxic activation by Fas agonists (E09, EP6, CH11, etc.) in tumor cells vs. Jurkat, it is conceivable to hypothesize differential influence of lipid rafts and associated proteins in tumor cells (vs. T or Jurkat cells) contributing toward more substantial autoinhibitory structural constraints to Fas activation in the membrane. In support of the latter, the findings of FasL salt bridges with PPCR agree with previously published studies with another death receptor, DR5 [3, 4, 44], where an autoinhibited and most constrained inactive form of DR5 closely associates and lays flat on the membrane. The PPCR engagement removes these autoinhibited structure constraints of receptors most effectively. Indeed, antibody strategies capable of effectively engaging DR5 PPCR were most efficacious in highly resistant tumor efficacy studies of ovarian and TNBC tumors [3, 4]. Similar to Apo2L binding of DR5, upon FasL binding, the cysteine-stabilized surface-exposed Fas PPCR residues are potentially involved in conformational Fas clustering, as described previously [3]. Unfortunately, unlike DR5, no direct Fas PPCR-engaging antibody has been described. However, as shown in Fig. 7, crude grafting of negatively charged residues in E09 antibody capable of partly engaging Fas PPCR region to a certain degree revived death agonism in tumor cells. Further investigations are needed to directly engage R87 with a new generation of Fas agonist antibodies. A mechanism similar to Fas PPCR not only operates in DR5, but potentially, such conformational looping mechanism supporting the role of positively charged patches also exists for EGFR activation under the negatively charged membrane in reliving electrostatic forces EGR-dependent activation [45]. Furthermore, not only overall expression but also differential distribution of DISC function regulatory proteins such as caspase-8, cFLIP, RIP, FAP-1, etc. in charged membrane rafts additionally contributes toward supplemental barrier to apoptotic threshold in cancer cells vs. Jurkat or T cells [46, 47].

One key and novel observation of our CAR-T Fas bystander assay with two different clinically relevant targets (FOLR1, NaPi2b) was that optimal off-target caspase-8 activation was highly dependent on antigen-positive cells. In contrast, the caspase-8 activity and death agonism in the case of the bispecific antibody approach (such as E09-FasL) was directly proportional to Fas expression (Fig. 9H vs. I). Considering the overall binding of death-activating ligands (Apo2L, CD40L, and FasL, etc.) against death receptors depends on multiple evenly distributed low-affinity interactions in the ectodomain receptors [21, 24, 25, 48], a high level of avidity toward Fas-positive (antigen-negative) cells mediated by neighboring antigen-positive cells remains crucial to engage Fas receptor effectively. As shown in Figs. 8 and 9, caspase-8 was activated significantly higher when CAR-T-specific antigen-expressing cells were co-cultured along with antigen-negative and huFas-expressing murine ID8 cells or exogenous Fas overexpressing Hey-A8 (endogenous Fas knocked out) cells. These findings of the requirement of T-cell avidity for optimal bystander caspase-8 activation not only support published studies [16, 17] but also conceptually advance the bystander CAR-T Fas-killing design via taking into account Ag-directed avidity. Suppose bispecific antigen-targeting CAR-T has a higher potential to activate bystander Fas/caspase-8 in avidity-mediated killing in heterogenous tumor beds. In that case, the latter will further amplify the CAR-T applicability toward hard-to-penetrate solid tumors. Nonetheless, considering that clinical testing of NaPi2b targeting antibody-drug conjugate strategies has been halted due to grade ≥3 adverse events in ovarian cancer [49], our preliminary findings lay the foundation for NaPi2b targeting CAR-T strategies for antigen-specific superior cytotoxicity and bystander killing of antigen-negative ovarian tumor cells.

In tumor-immune interactions, the antitumor outcome is guided by various paired stimulatory and inhibitory receptor families (including PD: PD-L1, CD155: TIGIT, CD80: CTLA4, etc.). Most biochemical studies have indicated higher affinity and binding priority for the inhibitory receptors over stimulatory receptors [50]. Similarly, compared to death-activating, death-inhibiting decoy receptors maintain a higher binding affinity against the FasL and Apo2L [24]. Hence, tumor overexpression of decoy death receptors remains the fundamental barrier to bystander CAR-T efficacy. If similar to armored CAR-T cells [51], which overcome the barrier of adverse tumor microenvironment by constitutively releasing inflammatory cytokines such as IL-12 (or armed to secrete other cytokines), engineered CAR-T cell capable of blocking the function of amplified decoy Fas receptors [52] (such as DCR3, etc.) in the heterogeneous tumor microenvironment to maintain superior bystander function is also an exciting area of investigation.

Despite numerous DR5 antibodies in clinical trials, not one Fas antibody has been made to phase-1 trials. The differential and effective cytotoxic activation of Jurkat cells by preclinical E09 and CH11 antibodies underscores the need for the next generation of Fas agonists with reverse tumor activation profiles with optimal T-cell safety. If the differentially regulated PPCR region of Fas (tumor vs. T cell) has the potential to lead us in this direction, it demands new antibody campaigns against this epitope. How in Fas-TCR interactions [40], TCR structurally interferes with Fas PPCR accessibility and downstream DISC signaling (and vice versa) in T cells is an exciting area of investigation [27]. Even more importantly, a potential design for the next generation of Fas agonist antibodies, which are sterically inhibited against Fas binding function due to its closeness to TCR, could be the path forward for Fas agonists for safer and successful clinical trials for solid tumors [53]. Notably, Fas is also oncogenic [11], metastatic, and has cancer stem cell regulating function [10] in tumors; such targeting represents a double-edged sword for solid cancer immunotherapy. Nonetheless, like DR5, additional Fas antibodies capable of targeting epitopes distributed throughout the Fas ectodomain could provide further clues for its differential activation in tumors and T cells.

## Experimental material (models) and methods details

### Mice strains

Six- to eight-week-old (age), 20–25-g (weight), both male and female (sex) mice were used for tumor xenografts generation and in vivo efficacy studies as described in the text (see Table 3). Athymic Nude (Envigo) *Foxn1*^*nu*^*/Foxn1*^*+*^ or NOD.Cg Prkdc^scid^ Il2rg^tm1Wjl^/SzJ also called NSG mice were used. All animal procedures were conducted under the accordance of the University of California Davis Institutional Animal Care and Use Committee (IACUC) and (DoD ACURO) approved protocols and conformed to the relevant regulatory standards.

**Table 3:** Details of commercial kits, antibodies, and other reagents.

Reagent table	Source	Identifier
Antibodies		
Caspase-8	Cell Signaling Technology	Cat #9746
Phospho-LAT (Tyr255) Antibody	Cell Signaling Technology	Cat #45170
Phospho-Zap-70 (Tyr319)/Syk (Tyr352) (65E4) Rabbit mAb	Cell Signaling Technology	2717
Phospho-PLCγ1 (Tyr783) (D6M9S) Rabbit mAb	Cell Signaling Technology	14008
CD3ε (CD3-12) Rat mAb	Cell Signaling Technology	4443
Phospho-Lck (Tyr505) Antibody	Cell Signaling Technology	2751
Phospho-SLP-76 (Ser376) (D9D6E) Rabbit mAb	Cell Signaling Technology	14745
Cleaved PARP antibody	Cell Signaling Technology	Cat #9953
Anti-Rabbit-HRP antibody	Cell Signaling Technology	Cat #7074
Anti-Mouse-HRP antibody	Cell Signaling Technology	Cat #7076
Folate Receptor alpha Polyclonal Antibody (FOLR1)	Invitrogen	Cat #PA5-24186
Cy5 conjugated Anti-Human IgG (H+L) Alexa Fluor® 488 AffiniPure Goat Anti-Rabbit IgG (H+L)	Jackson ImmunoResearch	Cat #709-175-149 Cat #111-545-003
Cy™5 AffiniPure Donkey Anti-Mouse IgG (H+L) Anti-Human IgG1 HRP	Jackson ImmunoResearch	Cat #715-175-150 Ref #A10648
Anti-Fas (Human)	R & D System	Cat #AF326
Anti-Fas (Human)	Cell Signaling Technology	Cat #4233
Anti-Fas (Human)	Cell Signaling Technology	Cat #8033
Anti-Fas (Human)	Abcam	Cat #ab82419
Cleaved Caspase-3 Antibody	Cell Signaling Technology	Cat #9661
Capase-3 Antibody	Cell Signaling Technology	Cat #9668
GFP Antibody (B-2)	Santa Cruz	sc-9996
GAPDH Antibody	Cell Signaling Technology	Cat #5174
Goat anti-Human IgG (Fc) PE	Thermo Fisher	Cat #124998-82
Goat anti-Human IgG (Fc) magnetic	Qiagen	Cat #310344
Bacterial and virus strains		
* E. coli* HST08, Stellar Cells	Takara BioScience	Cat #636766
Biological samples (antibodies)		
KMTR2	Imgt.org	See Supplementary Table S2
E09	Rscb.org	See Supplementary Table S2
EP6B	Rscb.org	See Supplementary Table S2
E09-EP6B bispecific	This paper	See Supplementary Table S2
EP6B-E09 bispecific	This paper	See Supplementary Table S2
E09-FasL^WT^	This paper	See Supplementary Table S2
E09-FasL^E163A^	This paper	See Supplementary Table S2
E09-FasL^D164A^	This paper	See Supplementary Table S2
E09-FasL^E270A^	This paper	See Supplementary Table S2
E09-FasL^E271A^	This paper	See Supplementary Table S2
EP6B-FasL^WT^	This paper	See Supplementary Table S2
Chemicals, peptides, and recombinant proteins		
His-FasL^WT^	This paper	See Supplementary Table S1
His-FasL^R156G^	This paper	See Supplementary Table S1
His-FasL^A247E^	This paper	See Supplementary Table S1
His-FasL^G277S^	This paper	See Supplementary Table S1
His-FasL^E163A^	This paper	See Supplementary Table S1
His-FasL^D164A^	This paper	See Supplementary Table S1
His-FasL^E270A^	This paper	See Supplementary Table S1
His-FasL^E271A^	This paper	See Supplementary Table S1
His-Fas^WT^	This paper	See Supplementary Table S1
His-Fas^R87A^	This paper	See Supplementary Table S1
His-FasL^MONKEY^	This paper	See Supplementary Table S1
His-Fas^MONKEY^	This paper	See Supplementary Table S1
His-Fas^WT^	This paper	See Supplementary Table S1
His_FasL^WT-WT^	This paper	See Supplementary Table S1
His_FasL^WT-R156G^	This paper	See Supplementary Table S1
His_FasL^WT-A247E^	This paper	See Supplementary Table S1
His_FasL^WT-G277S^	This paper	See Supplementary Table S1
His_FasL^WT-E163A^	This paper	See Supplementary Table S1
His_FasL^WT-D164A^	This paper	See Supplementary Table S1
His_FasL^WT-E270A^	This paper	See Supplementary Table S1
His_FasL^WT-E271A^	This paper	See Supplementary Table S1
Critical commercial assay reagents and kits		
HiPure Plasmid Maxiprep kit	Invitrogen	Cat #K21007
Infusion	Takara BioScience	STO344
Cell Proliferation Assay	TACS MTT Trevigen	Cat #4890-25-K
HiTrap MabSelect SuRe	GE Healthcare	Cat #11-0034-93
TMB Substrate Reagent Set	BD OptEIA	Cat #555214
CHO freestyle Media	Thermo Fisher	Cat #12651014
HiTrap MabSelect Sure column	GE	Cat #11003493
Protein A resin	Thermo Fisher	Cat #P153142
CHO CD efficient Feed B	Life Technologies	Cat #A1024001
Matrigel	Corning	Cat #354234
Corning® 500 mL RPMI 1640	Corning	Cat #10-040-CV
Corning® 500 mL DMEM (Dulbecco’s Modified Eagle’s Medium)	Corning	Cat #10-13-CV
Halt protease inhibitor	Thermo Fisher	Cat #78430
Goat anti-Human IgG (H&L) Coated Magnetic Particles, Smooth Surface	Spherotech	Cat #HMS-30-10
Cy5‐NHS Ester	Gold Biotechnology	Cat #B-430-1
Infusion	Takara BioScience	Cat #638920
CHO CD efficient Feed B	Life Technologies	Cat #A1024001
PEI transfection reagent	Thermo Fisher	Cat #BMS1003A
Stellar Cells	Takara BioScience	Cat #636766
BLI Biosensor	ForteBio	Cat #18-5019
Xenolight D-luciferin potassium salt	PerkinElmer	Cat #P/N 122799
PEI transfection reagent	Thermo Fisher	Cat #BMS1003A
HisPur Ni-NTA resin	Thermo Fisher	Cat #88221
EZ-Link Sulfo-NHS-SS-Biotin	Thermo Fisher	Cat #21331
Experimental models: cell lines		
Human: OVCAR3	Ovarian Cancer	ATCC HTB-161
Human: MDA-MB-436	TNBC	ATCC HTB-130
Human: MDA-MB-231	TNBC	ATCC HTB-26
Human: MDA-MB-231-2B	TNBC	ATCC HTB-26
Human: A549	Lung Cancer	ATCC CLL-185
Human: Cavo-3	Ovarian Cancer	ATCC HTB-75
Human: HCC1806	TNBC	ATCC CRL-2335
Mouse: ID8	Kind gift from Sharon stack	ATCC CRL-2539
Human: Hey-A8 Fas-KO	Kind gift from Dr Marcus Peters	Northwestern University
Human: Hey-A8 Fas^WT^	Generated in our laboratory (This paper)	Transient WT Fas-expressing cells
Human: Hey-A8 Fas^S83A^	Generated in our laboratory (This paper)	Transient WT Fas-expressing cells
Human: Hey-A8 Fas^R87A^	Generated in our laboratory (This paper)	Transient WT Fas-expressing cells
Experimental models: mouse		
Mouse: Athymic Nude Foxn1nu	Envigo	
Mouse: C56BL/6	Jackson Lab	
Software and algorithms		
Vector NTI	Thermo Scientific	N/A
GraphPad Prism	GraphPad Software	www.graphpad.com
FlowJo	FlowJo, LLC	www.flowjo.com
FCS Express	De Novo Software	www.denovosoftware.com
Chimera	UCSF Chimera	https://www.cgl.ucsf.edu/chimera/
Other		
ChemiDoc imaging system	Bio-Rad	NA
AKTA pure chromatography system	GE Healthcare	NA
Bio-Rad BioLogic LP chromatography system	Bio-Rad	NA
Minitron Incubator Shaker	INFORS HT	NA
Cytiva HiTrap™ Column	Cytiva/GE Healthcare	NA

### Cell lines

The cell lines used in the study are provided in Table 3. All the cell lines were maintained in Dulbecco’s modified Eagle’s medium (DMEM), MEM, RPMI 1640 or other required optimal medium supplemented with 10% heat-inactivated fetal bovine serum (FBS), 2 mM glutamine, 100 U/ml penicillin, and 100 µg/ml streptomycin (complete medium) unless otherwise specified as described [26]. Hey-A8 Fas knockout (KO) cells (kindly provided by Dr Marcus Ernst Peter, Northwestern University) were cultured in RPMI supplemented with 10% (vol/vol) FCS and 1 mM penicillin/streptomycin. Human PBMCs were purchased commercially and monkey PBMCs were kindly shared by Dr Smita Iyer and Dr Satya Dandekar, University of California Davis. Human and monkey PBMC were maintained in 20% FBS and 100 mM sodium pyruvate in RPMI 1640 media supplemented with glutamax (Gibco) and 1% penicillin/streptomycin (Gibco) before enriching them into CD3-enriched T cells using CD3/CD28 agonists. Various adherent tumor cell lines were trypsinized and expanded as follows: after digestion, the cell suspension was neutralized with complete media and centrifuged 5 min at 1500 rpm. The cell pellets were suspended in relevant DMEM/RPMI media and either expanded or seeded after counting using Countess II (Life technologies). Passaged cell lines were routinely tested for mycoplasma using MycoAlert Detection Kit (Lonza).

### Preclinical Fas antibodies

The sequences of various Fas agonist monospecific and bispecific antibodies and dual-specificity Fas agonist and FasL molecules used in this manuscript are provided in Table 2.

### Recombinant antibody cloning

The sequences of various clinical Fas agonist antibodies generated are provided in Table 1. All antibodies were cloned and expressed as described previously by our lab [3, 26, 35]. Wherever indicated bispecific antibodies were engineered by genetically linking CH3 Fc to either Fas agonist scFv’s or FasL, etc., using flexible linker as described earlier [26]. The DNA sequences for antibodies or various His-tagged or Fc-tagged protein were retrieved from open sources (such as pubmed.gov, or publicly available patents or rscb.org, PDB database) and synthesized as gene string using Invitrogen GeneArt gene synthesis services. After PCR amplification, DNA was gel purified and inserted into pCDNA 3.1 vectors (CMV promoter) by making use of In-Fusion HD Cloning Kits (Takara Bio). EcoR1 and HindIII digested vector was incubated with overlapping PCR fragments (of various recombinant DNAs, see list of clones in Table 3) with infusion enzyme (1:2, vector: insert ratio) at 55 °C for 30 min, followed by an additional 30 min incubation on ice after adding *E. coli* Stellar^TM^ cells (Clontech, see reagent Table 3). Transformation and bacterial screening were carried out using standard cloning methods. Positive clones were sequenced and confirmed in a 3-tier method. Confirmed bacterial colonies were Sanger sequencing upon PCR followed by re-sequencing of mini-prep DNA extracted from the positive colonies. Finally, maxipreps were re-sequenced using both forward, reverse and middle primers prior to each transfection. Recombinant antibodies were also re-confirmed by ELISA and flow cytometry surface binding studies as described earlier [26].

### Recombinant His-tagged and Fc-tagged FasL and fas cloning

Recombinant antibody Cloning, both His-tagged and Fc-tagged FasL and Fas were cloned similarly in expression as described and published earlier by our lab [3, 26, 35]. In case of FasL His-tag and Fc-tag were added to the N-terminal, while for Fas His-tag and Fc-tag were added to the c-terminal as indicated in the manuscript description. Various described mutations of alanine scanning were generated by mutating PCR primers as described earlier [26]. Similar tandem FasL constructs were cloned with N-terminal His-tag.

### Recombinant antibody and recombinant protein expression

Various recombinant antibodies (and recombinant ligands) were used in this study, and recombinant target antigens were engineered, expressed, and purified in Singh Laboratory of Novel Biologics as described earlier [26]. Briefly, FreeStyle CHO-S cells (Invitrogen, Reagents Table 3) were cultured and maintained according to the supplier’s recommendations (Life technologies) biologics using the Free-Style CHO expression system (Life technologies) and as previously described [26, 54]. A ratio of 1:2 (light chain, VL: heavy chain, VH) DNA was transfected using 1 μg/ml polyethyleneimine (PEI, see Table 3). After transfections, cells were kept at 37 °C for 24 h. After 24 h, transfected cells were shifted to 32 °C to slow down the growth for 9–12 additional days. Cells were routinely fed (every 2nd or 3rd day) with 1:1 ratio of Tryptone feed and CHO Feed B (see Reagents Table 3). After 10–12 days, supernatant from cultures was harvested and antibodies were purified using protein A affinity columns as described in the antibody purification section. Recombinant His-tagged FasL, Fas, FOLR1, etc., were purified using Ni-Sepharose HisTrap columns. The activity of His-FasL generated in our laboratory was confirmed (alongside commercial His-FasL) using multiple cancer lines and was found to be exactly similar. Similarly, the activity of commercial OKT3 or TGN1412 antibodies was compared next to recombinant OKT3 and TGN1412 antibodies generated in our laboratory using various cancer cell lines as described earlier [26].

### Antibody and recombinant protein purification

Various recombinant antibodies used in this study and recombinant target antigens were affinity purified using HiTrap MabSelect SuRe (GE, 11003493) columns in Singh Laboratory of Novel Biologics as described and published earlier [3, 26, 35]. After 10–12 days, the transfected cultures were harvested via separating cell pellets from protein-containing soup by spinning at 3000 rpm for 60 min followed by flirtation through 0.2-micron PES membrane filters (Millipore Express Plus). Cleaning-in-place was performed for each column as per manufacturer (Cytiva) recommendations. Following resin cleaning, columns were washed three times with binding buffer as an example: 20 mM sodium phosphate, 0.15 M NaCl, pH 7.2 for protein A columns and as per manufacturer (Cytiva) recommendations for His-Trap columns. Filtered supernatant containing recombinant antibodies or antigens or Fas ligands were passed through the columns at 4 °C. Prior to elution in 0.1 M sodium citrate, pH 3.0–3.6, the columns were washed three times with binding buffer (pH 7.0) for protein A columns and as per manufacturer (Cytiva) recommendations for His-Trap columns. The pH of eluted antibodies was immediately neutralized using sodium acetate (3 M, pH 9.0) for protein A columns and as per manufacturer (Cytiva) recommendations for His-Trap columns. After protein measurements at 280 nm, antibodies were dialyzed in phosphate-buffered saline (PBS) using Slide-A-Lyzer 3.5K (Thermo Scientific, 66330). As indicated above, recombinant His-tagged FasL, Fas, FOLR1, etc., were purified using Ni-Sepharose HisTrap columns. Antibodies and recombinant proteins were run on gel filtration columns (next section) to analyze the percent monomers. Whenever necessary, a second step size exclusion chromatography (SEC) was performed. Recombinants IgG4-Fc tagged ECDs of various Fas mutants, etc., were also similarly harvested and purified using protein A columns as described earlier [3, 26, 35].

### Size exclusion chromatography

The percent monomer of purified antibodies and ligands etc. (as indicated) was determined by SEC as described and published by our lab for various preclinical DR5 monospecific and bispecific antibodies [3, 26, 35]. Briefly, 0.1 mg of the purified antibody was injected into the AKTA protein purification system (GE Healthcare Life Sciences), and protein fractions were separated using a Superdex 200 10/300 column (GE Healthcare Life Sciences) with 50 mM Tris (pH 7.5) and 150 mM NaCl concentration. The elution profile was exported as an Excel file and a chromatogram was developed. The protein sizes were determined by comparing the elution profile with the gel filtration standard (Bio-Rad 151–1901) as described earlier [3]. All protein peaks observed in the void fractions were considered as aggregate. Hence, the area under the curve was calculated for each peak, and a relative percent monomer fraction was determined as described earlier [3, 26, 35].

### Binding studies by ELISA

Binding specificity and affinity of various described IgG1 subclasses were determined by ELISA using the recombinant ECD either His-tagged Fas or IgG4-Fc-tagged Fas as described earlier by our lab for DR5 agonist antibodies [3, 26, 35]. Binding specificity and affinity of various FasL PPCR mutants and ALPS mutants were determined using IgG4-Fc-tagged Fas using ELISA. For coating 96-well ELISA plates (Olympus), the protein solutions (2 μg/ml) were prepared in coating buffer (100 mM Sodium Bicarbonate pH 9.2) and 100 μl was distributed in each well. The plates were then incubated overnight at 4 °C. The next day, the unbound areas were blocked by cell culture media containing 10% FBS, 1% bovine serum albumin (BSA) and 0.5% sodium azide for 2 h at room temperature. The serial dilutions of antibodies or recombinant proteins (2-fold dilution from 50 to 0.048 nM) were prepared in blocking solution and incubated in target protein-coated plates for 1 h at 37 °C. After washing with PBS solution containing 0.1% Tween20, the plates were incubated for 1 h with horseradish peroxidase-conjugated anti-human IgG1 (Thermo Scientific, A10648). Detection was performed using a two-component peroxidase substrate kit (BD biosciences), and the reaction was stopped with the addition of 2N Sulfuric acid. Absorbance at 450 nm was immediately recorded using a Synergy Spectrophotometer (BioTech), and background absorbance from negative control samples was subtracted. The antibody affinities (Kd) were calculated by nonlinear regression analysis using GraphPad Prism software.

### In vitro cell viability assays

Cell viability following FasL^WT^, various FasL mutant single or tandem FasL, lexatumumab, KMTR2, E09, EP6, E09-EP6 bispecific, E09-FasL, etc. treatments (as indicated in various figures) either alone or in combination with an anti-Fc reagent were determined using the AlamarBlue cell viability assays and MTT cell proliferation assays as per manufactured protocols as described earlier by our lab for DR5 agonists [3, 26, 35]. Briefly, cells (indicated cells in the main text or figure legends) were treated with increasing concentrations of various antibodies (as indicated) along with relevant positive and negative control antibodies for 6, 24, or 48 h (as indicated according to the experiment). For each cell-killing assay, the figures show the representative profiles from *n* = 2–4 with different cultured confluency. Whenever used for immunoblotting, following antibody treatment caspase-3 processing in tumor cells was monitored using selective antibodies that recognize cleaved human caspase-3 or total caspase-3 (Cell signaling, 9661 and 9668). Fas oligomerization was determined using immunoblotting assays (cell signaling Rabbit mAb, 4233). Cell viability was additionally examined by flow cytometry-based apoptotic detection methods using 7-ADD exclusion from live cells as described earlier [26].

### IC_50_ determination

Indicated IC_50_ values for various cell-killing assays were calculated as described earlier by our lab [3, 26, 35]. Briefly, cells were cultured in 96-well plates. The following day, when cultures became adherent (tumor cells) or suspension (Jurkat or T cells), they were treated with increasing concentrations of the antibodies or ligands or combinations, etc. (as indicated) for 36–48 h at 37 °C (5% CO_2_). All antibodies used for treatment were dialyzed in PBS (typically having a pH of 7.0–7.5) and concentration was measured prior to treatments. The readings obtained were normalized to IgG control, and IC50 values were calculated using GraphPad Prism software using nonlinear dose-response regression curve fits as described previously [3, 26, 35]. The final data shown in the typical histograms were obtained from at least three independent experiments (*n* = 3).

### Western blotting

All western blot protocols were followed as described earlier by our lab [3, 26, 35]. Briefly, various indicated cell lines (Tumor or Jurkat or T cells) were grown overnight in tissue culture plates. After indicated treatments for indicated times, cells were rinsed with PBS and then lysates were generated using RIPA buffer supplemented with protease inhibitor cocktail. Lysates were cleared and separated by spinning the samples at 14,000 rpm for 30 min. Protein concentrations were determined using Pierce BCA protein assay kit followed by Western blotting using the Bio-Rad SDS-PAGE Gel system. Approximately 30 µg of protein was resolved on 10% SDS gels (reduced or partly reduced or partly denaturing as indicated in figure legends), followed by wet transfer onto PVDF membrane. Membranes were blocked in TBS + 0.1% Tween (TBST) with 5% non-fat dry milk (or BSA for phosphorylation blots) at room temperature. Membranes were incubated with indicated primary antibodies overnight, rocking on the shaker at 4 °C. Next day, membranes were washed in TBST (3×, 10 min each), followed by incubation with anti-rabbit or anti-mouse secondary antibodies, etc., as indicated (1/10,000 dilution) for 1 h at room temperature on a shaking platform. Membranes were washed in TBST (3×, 10 min each), and Immunocomplexes on membrane blots were detected with SuperSignal West Pico Chemiluminescent Substrate (Thermo Fisher Scientific) using a Bio-Rad Gel Doc Imager system. Sources of various primary antibodies are listed in Table 3.

### Pre-neutralization assays

Described pre-incubation and pre-neutralization protocols were followed as described earlier by our lab [3, 26, 35]. Briefly, the corresponding receptors used in neutralization (such as indicated recombinant Fas or rFas, etc.) were incubated with corresponding antibodies or ligands. Whenever indicated throughout the manuscript text or in figure legends, variable domain pre-neutralization of Fas agonist antibodies or FasL or CAR-T, etc., was carried out. For in vitro and in vivo studies, indicated antibodies and indicated recombinant antigens (Fas^WT^ or Fas^R87A^, rFOR1, etc.) were either precoated on plated in the presence of 0.01% gelatin or incubated together at 37 °C for 1 h shaking on a platform. As a control, indicated non-neutralizing non-specific proteins (such as rFOLR1 for Fas antibodies) were also incubated at 37 °C for 1 h shaking on a platform with PBS. Following pre-neutralization, antibodies were either used in vitro for cell-killing assays, or CAR-T cell lysis assays or for cellular/tumor lysates generation (immunoblotting), or for caspase-8 activity assays, etc., as indicated.

### Flow cytometry

The cell surface expression of Fas, CD8, CAR-T scFv, GFP, HuFas in chimeric ID8 cell lines, etc., and other indicated surface proteins were analyzed by flow cytometry as described earlier by our lab [3, 26, 35]. Overnight grown cells were trypsinized and suspended in FACS buffer (PBS containing 2% FBS). The single-cell suspension was then incubated with primary indicated antibodies for 1 h at 4 °C with gentle mixing. Wherever indicated PFA was using for crosslinking the antibodies. Following wash with FACS buffer, the cells were then incubated with the fluorescently labeled secondary antibody for 1 h. Cells were washed and flow cytometry was performed using FACSCalibur. The data were analyzed by FCS Express (De Novo Software) and FlowJo.

### Native fas immunoprecipitation studies with preclinical antibodies

Native Fas immunoprecipitation studies with clinical antibodies were performed similar to as described earlier by our lab [3, 26, 35]. Briefly, cells were cultured in tissue culture dishes for 24 h prior to treatment. Before treatment, the culture medium was washed with PBS and replaced with a serum-free medium. Cells were treated with indicated Fas agonists (IgG1-Fc) antibodies (10, 50 or 500 nM) for an indicated time as described in the text and figures. Cells were harvested and lysed with IP lysis buffer (20 mM Tris pH 7.5, 150 mM NaCl, 1 mM EDTA, 10% Glycerol, 1% Triton-X, 0.5 mM PMSF) supplemented with protease inhibitor cocktail (Thermo Scientific). Samples were spun at 14,000 rpm for 30 min clear protein lysates were collected, and protein was quantified by Pierce BCA protein assay kit. 1–1.5 mg protein (~400–500 µl) was taken into the Eppendorf microcentrifuge tube. Protein lysates were incubated (shaking platform) with anti-human IgG1-Fc specific beads for 2 h at 4 °C, placing them into a rotating wheel. Protein-conjugated beads were washed three times with PBS. Finally, beads were boiled at 100 °C for 5 min with 30 µl of SDS sample buffer. The 15–20 µl sample was loaded into the SDS-gel, and western blotting was performed using the Bio-Rad SDS-PAGE Gel system, followed by immunoblotting using Fas, caspase-8, etc. specific antibodies as indicated in various figure legends.

### huFas chimeric, Fas with various mutations in PPCR region, etc., stable transient line generation

Various stable or transient transfections were carried out by our lab while generating published DR5 stable lines [3]. Briefly, transfection of various Fas constructs into the Hey-A8 Fas KO tumor cell lines was achieved by jetOPTIMUS DNA transfection reagent for recombinant Fas (wildtype or mutated) cloned in pCDN3.1 vector. In brief, 60–70% of confluent cells were grown in a 10 cm culture dish. Mixing 10 µg of plasmid DNA and 10 µl of transfection reagent into 1 ml of jetOPTIMUS buffer made transfection solution. After incubating for 10 min at room temperature, the transfection mix was added to the cells. The cells were further allowed to grow for 24 h and then selected using 2 mg/ml of G418. In detail, 10 µg DNA was diluted into 1000 µL jetOPTIMUS buffer and vortexed. This was followed by the addition of 10 µL jetOPTIMUS into the DNA solution (ratio 1:1 corresponding to µg DNA: µL reagent) and vortexed and spun down briefly. The mixture was incubated for 10 min at room temperature. Next, the transfection mix was added dropwise onto the cells in a serum-containing medium and distributed evenly. Plates were incubated at 37 °C for 24 h. The next day transfection medium was replaced with by cell growth medium and cells were allowed to grow for another day before starting G418 (2 mg/ml) selection. Media was changed every day, and a reliable GFP signal was evident 72 h of transfection. For long-term stable line generation, lentiviral method was used as described in the next section.

### Lentiviral preparation and transduction

Lentiviral packaging and delivery were executed using the technology from system Biosciences, and the method was very similar to that described here [55]. Briefly, after cloning of either Fas constructs or CAR-T constructs into lentiviral vectors, lentivirus was prepared by transfecting 293T cells in a T75 flask with transfer vector (6 μg) and packaging vectors (3 μg each) in the ratio of 2:1:1:1 using 30 µg of PEI. The virus-containing culture medium was collected 48 and 72 h after transfection, cleared by filtration (0.45 μm Millipore, Bedford, MA) and concentrated by 20% PEG 6000. After centrifugation at 3000 g for 30 min, the pellet was resuspended in 1/10th of the initial volume in PBS/0.1% BSA, stored at −70 °C. For transduction, the 60–70% confluent cells were plated in a 10 cm plate, and 5 ml virus along with 5 µg/ml polybrene was added. Transduction medium was replaced with growth medium after 12 h and cells were allowed to grow for another 24 h. The transduced huFas chimeric positive cells were selected using 2.5 μg/ml puromycin. In detail, HEK-293T cells were cultured in high-glucose DMEM (Corning) supplemented with 10% FBS (Corning) at 37 °C in 5% CO_2_ incubator. For transfection, the cells were grown a day prior at 70–80% confluency in 10 cm culture dish. The transfection mix was prepared as follows as described earlier [3]: transfer plasmid with gene of interest such as Fas (6 μg), pVSVG plasmid (3 μg), pREV (3 μg), pRRE (3 μg), OptiMEM media (500 μl) and PEI (30 μg). The transfection mixture was briefly vortexed, followed by a quick centrifugation step followed by incubation at room temperature (10 min). The transfection mixture was then gently added on top of cells through the wall and mix by tilting the plates a couple of times. Transfected cells were incubated at 37 °C for 12–16 h (5% CO_2_), and the fresh medium was added the next day. Virus-containing culture media soup was collected after 48 and 72 h. The floating cellular debris was separated by quick spin at 1000 rpm for 5 min and then virus-containing culture medium soup was filtered through 0.45 μm Millipore filters. The virus-containing soups were concentrated via PEGylation in the following ratio as described earlier [3]: Virus suspension (40 ml), 50% PEG 6000 (10 ml) and 5 M NaCl (1 ml). PEGylated solution was mixed and incubated at 4 °C overnight on a rotator. The precipitated virus particles were centrifuged at 4000 rpm for 30 min and resuspended in approximately 4 ml of PBS. To transduce, the tumor cells or CD3-enriched T cells were seeded a day before at a confluency of 60–70% in a 10 cm culture plate. Transduction solution was prepared by mixing 2 ml of the virus, 1–5 μg/ml of polybrene plus 1× HEPES buffer and was gently added to the top of cells with 8 ml of culture medium. Cells were allowed to grow at 37 °C (12 h minimum) and then the medium was replaced with a growth medium. The puromycin selection (2.5 μg/ml) was performed after 48 h of transduction, and the medium was replaced each day with intermediate PBS washing to avoid the accumulation of dying cell debris. Stable cells appeared within a week. For CAR-T cells following lentiviral transduction, T cells were cultured in recombinant FOLR1 (rFOLR1) precoated plates to expand the cells. To enrich farletuzumab CAR-T, a second set of lentiviral transductions was carried out 4 days after the first transduction, similar to the previous step.

### Tumor xenograft studies

All animal procedures were conducted according to the University of California IACUC and (DoD ACURO) approved protocols and conform to the relevant regulatory standards. Details of mice strains, age and sex used are provided above. Briefly, 6- to 8-week-old (age), 20–25-g (weight), both male and female (sex) mice were used for tumor xenografts generation, in vivo efficacy studies and were carried our as described previously [3, 26, 35]. The mice stain NOD.Cg Prkdc^scid^ Il2rg^tm1Wjl^/SzJ (also called NSG mice) was used for FasL mutant antitumor studies. OVCAR3 ovarian tumor cell lines were used for tumor nude xenograft studies as described in the text. Weight and age (6–8 weeks old) matched mice (more than 6) were injected subcutaneously in their right flank in Matrigel as indicated. Tumor cells were mixed 100 μl volume with Matrigel. For antitumor efficacy studies, at least 3 or more mice bearing ∼100 mm^3^ tumors were weight-matched as indicated in figure legends. The animals were randomly assigned into groups and injected (25–50 μg doses as indicated) intraperitoneally as described in our previous studies [3, 26, 35]. The antibodies or ligand doses were given two or three times per week as described previously. Tumors were measured in two dimensions using a caliper as described previously [20, 26, 35, 56]. Tumor volume was calculated using the formula: *V* = 0.5*a* × *b* [2], where *a* and *b* are the long and the short diameters of the tumor, respectively (*n* = 4–6 animals were used for each therapeutic antibody/ligand injection). The *p* values are determined by a two-tailed paired Wilcoxon Mann–Whitney test [57].

### Binding studies by Bio-Layer interferometry (BLI)

Binding measurements were performed using Bio-Layer Interferometry on FortéBio Red Octet 96 instrument (Pall) as described earlier [26]. IgG1 (AHQ) and His (Ni-NTA) based sensors were employed for the studies to detect recombinant His-tagged Fas and Fc-tagged Fas according to the manufacturer’s instructions. In some cases, biotin-Streptavidin-based sensors were employed for the studies. Recombinant IgG4-Fc linked Fas variants were biotinylated using EZ-Link Sulfo-NHS-SS-Biotin (Thermo Scientific 21331) following the manufacturer’s instructions. Unreacted Sulfo-NHS-SS-Biotin reaction was quenched by 50 mM Tris-Cl pH 7.4 and removed via dialyzing against PBS. For binding analysis, either His-tagged antigens or biotinylated antigens (1 µg/mL) were immobilized on biosensors (Sartorius) for 300 s to ensure saturation. Associate and dissociation reactions were set in 96-well microplates filled with 200 µL of Fas agonists for 900 s. All interactions were conducted at 37 °C in PBS buffer containing 2 mg/ml BSA. Kinetic parameters (K_ON_ and K_OFF_) and affinities (KD) were analyzed using Octet data analysis software, version 9.0 (Sartorius).

### Fas clustering assays

The receptor clustering assays were carried out similar to previously published DR5 clustering assays [3]. Hey-A8, Jurkat, OVCAR3, PBMC-derived CD3-enriched T cells, etc., were cultured at 37 °C, 5% CO_2_ in DMEM medium supplemented with 10% FBS. 0.3 × 10^6^ cells were seeded in each well of 6-well plates and grown at the above-mentioned conditions. When cells reached 80–90% confluency, the experiment was started. First, the old media was removed, followed by washing of cells with pH 8.0 PBS. Then, cells were incubated with Fas agonist antibodies or FasL (WT or mutants) in pH 8.0 PBS for 1 h at 4 °C with slow rotation. Just before use, 50 mM BS3 solution was prepared (freshly) by dissolving 10 mg BS3 in 350 µl of water. 2 mM BS3 crosslinker working solution was added to each well of the 6-well plate. The plate was allowed for crosslinking reaction at room temperature for 30 min. After crosslinking, the sample was quenched and unreacted BS3 with 40 mM Tris-HCl pH 7.5 at room temperature for 10–15 min. Finally, cell lysate was prepared in RIPA buffer and immunoblotting was carried out in non-reducing and partly denaturing conditions.

### FasL-mediated negative TCR signaling assays

Human PBMC-isolated healthy donors were purchased from StemCell Technologies. Naive T cells were separated from the whole PBMC T cell pool by using anti-CD45 (Cl. UCHL1) and anti-CD25 (Cl. M-A251; BD) beads by magnetic separation. For T-cell activation assays, plates were coated with 0.1 µg/ml anti-CD3 (OKT3) and 0.01 µg/ml anti-CD28 (TGN1412). To test the negative regulation of TCR signaling by FasL, T cells were serum starved overnight in a complete medium supplemented with 0.5% FCS. The next day, T cells were cultured on CD3/CD28 antibody-coated plates along with Fc-containing DB-FasL mostly or immobilized FasL on plates as described previously [27]. 2 µg/ml Fc-tagged DB-FasL (generated in or lab) was added when cells were transferred on to CD3/CD28 agonists coated plates. After various indicated times, the T-cell cultures were lysed in 50 mM Hepes, 150 mM NaCl, 1% IGEPAL CA-630, 2 mM PMSF, 2 mM EDTA, 1 mM Na_3_VO_4_, 50 mM NaF, and 10 mM Na_4_O_7_P_2_ × 10 H_2_O for 15 min, and protein lysates were subjected to immunoblotting analysis using phospho-LAT, phospho-PLC-gamma, phospho-ZAP70, pSLP-76, pLck and CD3 zeta etc., antibodies as described previously [27].

### CAR-T caspase-8 assay and Granzyme B ELISA

Human CD8 enriched T cells (~200 K) were co-cultured with target cells (OVCAR3 or GFP-ID8^huFas^, or ID^REGULAR^ or Colo-205-GFP etc. with various effector-target (E/T) ratio of (such as 6:1, 4:1, etc.) either for indicated times (2, 4, 6 or 8 h for caspase-8 assay) of for 24 h (cell lysis/killing assay). For these studies, multiple technical replicates but at least two biological replicates were carried out. For caspase-8 assays, the reagents were added to the cultures at indicated times. For cell lysates, cells are collected in RIPA bugger. For ELISA, supernatants of the co-cultured cells were harvested, and Granzyme B levels were measured by ELISA kits (R&D systems, Cat. #DY2906-05) as described previously [58]. All assays were performed according to the operating manuals. Similar to CAR-T^FOLR1^ plus OVCAR3 and GFP-ID8^huFas^ or ID^REGULAR^, etc., co-cultures as indicated in Figs. 8 and 9, CAR-T^NaPi2b^ plus OVCAR3 and Hey-A8^FasKO^ transiently transfected with WT or mutant Fas were tested as indicated in Figs. 8 and 9.

### Quantitation and statistical analysis

Data, unless indicated in figure legends, are presented as mean ± SEM. In general, when technical replicates were shown for in vitro experiments, a Student *t*-test was used for statistical analysis, and the same experiment was at least repeated once with a similar trend observed. When data from multiple experiments were merged into one figure, statistical significance was determined by a Wilcoxon Mann–Whitney test using GraphPad Prism 5.0 software as described earlier [26]. Tumor growth curves are displayed as mean ± SEM. For all the statistical experiments, the *p* values, *p < 0.05, **p < 0.01, and ***p < 0.001, were considered statistically different or specific *p* values indicated otherwise or “ns” indicates non-significant.

### Supplementary information

Supplementary Material All Raw Western Files

Supplementary Figures and Legends

Supplementary Figure 1, Figure S1

Supplementary Figure 2, Figure S2

Supplementary Figure 3, Figure S3

Supplementary Figure 4, Figure S4

Supplementary Figure 5, Figure S5

Supplementary Figure 6, Figure S6

Supplementary Figure 7, Figure S7

Supplementary Figure 8, Figure S8

Supplementary Figure 9, Figure S9

Reproducibility checklist

## Data Availability

Our manuscript does not contain RNA-Seq or other Large Data sets. PDB structure codes used for analysis in the manuscript are available at Protein Data Bank: https://www.rcsb.org/. Detailed sequences of antibodies and other ligands used in the manuscript are provided in tables. Raw western blots are available in the Supplementary file. Requests for reagents should be directed to JT-S (jtsingh@ucdavis.edu).
