# Supplementary material for: Characterizing the regulatory Fas (CD95) epitope critical for agonist antibody targeting and CAR-T bystander function in ovarian cancer
Source: Cell Death Differ. 2023 Oct 14;30(11):2408–31. doi: 10.1038/s41418-023-01229-7 (PMC10657439; doi:10.1038/s41418-023-01229-7)

**Supplemental Figures and Legends**

**Characterizing the regulatory Fas (CD95) epitope critical for agonist antibody targeting and CAR-T bystander function in ovarian cancer**

Tanmoy Mondal^1, 2^, Himanshu Gaur^1, 2^, Brice E.N. Wamba^1, 2^, Abby Grace Michalak^1, 2, 3^, Camryn Stout^1, 2, 3^, Matthew R Watson^1, 2, 3^, Sophia L. Aleixo^1, 2, 3^, Arjun Singh^2^, Salvatore Condello^4^, Roland Faller^5^, Gary Scott Leiserowitz^6, 7^, Sanchita Bhatnagar^2, 7^ and Jogender Tushir-Singh^1,2,7, 8*^

^1^Laboratory of Novel Biologics,

^2^Deartment of Medical Microbiology and Immunology, UC Davis School of Medicine

^3^Undergraduate Research Program Volunteers, University of California Davis, CA

^4^Department of Obstetrics and Gynecology, Indiana University School of Medicine

^5^Department of Chemical Engineering, University of California Davis, CA

^6^Department of Obstetrics and Gynecology, UC Davis School of Medicine

^7^UC Davis Comprehensive Cancer Center, UC Davis School of Medicine

^8`^DoD Ovarian Cancer Academy Early Career Investigator

*Correspondence: jtsingh@ucdavis.edu

Supplemental Figures:

**Figure S1**

(A) The structure of DR5 ECD in complex with Apo2L. A space-filling model is shown with DR5 (dark grey) and Apo2L (Light green). PPCR residues are shown in Red, and D203 of Apo2L is blue.

(B) The structure of DR5 ECD in complex with Apo2L. A ribbon trace structure is shown near the PPCR interface of DR5 (dark grey) and Apo2L (Light green). PPCR residues are shown as red sticks, and Apo2L D203 is shown as blue sticks (PDB: 1D0G)

(C) Cell survival assay of OVCAR3 with the increased concentration of Apo2L (WT) and Apo2L (D203A).

(D) The structure of DR5 ECD in complex with Apomab antibody. A space-filling model is shown with DR5 (dark grey) and Apomab VH (Light green), and Apomab VL (sky blue). PPCR residues are shown in Red, and D30 and D31 of VH are shown in blue.

(E) The structure of DR5 ECD in complex with Apomab heavy chain. A ribbon trace structure is shown near the PPCR interface of DR5 (dark grey) and Apomab VH (Light green). PPCR residues are shown as red sticks, and Apomab VH D30 and D31 are shown as blue sticks. Hydrophobic residues stabilizing D30 and D31 are shown as orange sticks (PDB: 40D2)

(F) Cell survival assay of OVCAR3 with the increased concentration of Apomab (WT) and Apomab (D30A), Apomab (D31A), and Apomab (DDAA:D30A, D31A).

Error bars in (C) and (F) represent SEM (n=3).

**Figure S2**

(A-C) A ribbon trace structure of FasL: DCR3 (PBD: 4MSV). The top and side view of the FasL trimer shows DCR interacting with DE, AA', EF, and GH loops. The dotted circles show the region of FasL and DCR3 involved in interactions. FasL is shown light blue, and DCR3 is gold.

(D-F) Same as B and C, except a space-filling model is shown. In the FasL trimer, the DE loop is highlighted in light green, AA' and GH loop in dark green, and CD and EF' loop in cyan colors. The corresponding interface in DCR3 is shown except R89, the key positively charged residue, which spacely orients to the corresponding R86 (See Fig 2) of Fas, is shown in pink.

(G-I) A space-filling model of FasL: Fas was generated after the superimposition of FasL from PDB: 4MSV with Fas from 3TJE. Similar to D-F, the DE loop is highlighted in light green, and AA' and GH loop is in dark green; however, CD and EF' loop are in yellow colors for FasL. The blue region highlights the negatively charged pocket in the trimer, with E163 and E271 being at the center of it (see Fig 2). The PPCR and surrounding area are highlighted in red, and part of Fas CRD3, which interacts hydrophobically with the CD and EF loop of FasL, is shown in pink.

**Figure S3**

(A) Comparison of Fas mediated cell killing of Jurkat cells with our lab generated His-tagged FasL and commercial Flag-tagged FasL. IC_50_ values are shown.

(B) CHO cells expressing various FasL mutants (A) were pelleted, soups were purified using HisTrap Ni Sepharose and were run on reducing gel along with an IgG1 for size confirmation

(C) Top: The amino acid residues of A-A' loops are shown for FasL^WT^, FasL^E163A^, and FasL^TL1A^ mutant. Mutant residues in a particular ligand are shown in Red. Bottom: Binding of indicated His-tagged FasL mutants to 96 well immobilized Fc tagged Fas by ELISA.

(D) Jurkat cells were treated with indicated His-tagged FasL, followed by immunoprecipitation using anti-His antibody and immunoblotting using anti-Fas antibody. Total Fas levels were equal in all samples.

(E) Cell killing assay of OV90 and Hey-A8 cells 36 hrs after treatment of indicated FasL mutants with the increasing concentration

(F) Jurkat cells were treated with indicated FasL mutants on top for 1hr, followed by

BS3 crosslinker treatment to capture the membrane clustered Fas. Lysates were run

in a non-reducing and partially denaturing gel

Error bars in (E) represent SEM (n=3+).

**Figure S4**

(A) Top: Schematic showing genetic construction of anti-PRADAXA antibody idarucizumab (also called dabigatran or DB for short form). Bottom: Schematic showing genetic construction of generation of dabigatra Fc-conjugated FasL with with various indicated mutations in ligand.

(B) CHO cells expressing indicated constructs were purified using protein-A and were

run on reducing gel for size confirmation.

(C) Indicated cell lines were treated with either IgG1 control, E09 IgG1, DB-FasL^WT^, or DB-FasL^E163A^ or DB-FasL^D164A^ or DB-FasL^E270A^ or DB-FasL^E271A^, followed by cell survival analysis (n=3+).

(D) Hey-A8^Fas-KO^ cells were transiently transfected with various Fas constructs with indicated mutations. 48hrs later, lysates were analyzed for total Fas expression using an anti-Fas antibody.

(E) Same as I, except after 48hrs of transfection, cells were treated with DB-FasL^WT^ followed

by caspase-8 activity assay for indicated times.

(F) CD3 enriched monkey PBMCs derived T-cells were treated with either CD3/CD28

agonists alone or indicated Fas mutants alone or together for 60 minutes, followed by

immunoblotting of indicated T-cell scaffold proteins and kinases.

Error bars in (C) represent SEM (n=3+).

**Figure S5**

(A) Ribbon diagram showing zoomed FasL trimer interface in sky blue (1), green (2), and pink (3) colors (PDB: 4MSV). Trimer interface is shown with dotted circle.

(B) Ribbon diagram showing zoomed FasL trimer interface in sky blue (1), green (2), and pink (3) colors (PDB: 4MSV). Hydrophobic residue Y192 (colored orange and represented with sticks) on the C beta-strand of 1st FasL monomers (sky blue C1) is labelled and highlighted. G277 which lacks sidechain, is on the H beta-strands of 1st FasL monomers (sky blue H1) is colored dark blue and is labelled and highlighted.

(C) Same as B, except the ALPS mutant S277 (substituted with G277) in H1 strand is colored dark blue and is labelled and highlighted. Dotted circles around C-H strands in all three FasL monomer shows potential clashes of serine sidechain with Y192 (atomic distance between S277-Y192 is only 1.22Å).

(D) Ribbon diagram showing zoomed FasL trimer interface in sky blue (1), green (2), and pink (3) colors (PDB: 4MSV). R156 with side chain (pink and represented with stick) in the A-A' loop of highlighted 2^nd^ FasL monomer (green) along with surrounding flexible residues S155, S157, N158, and S159 (green sticks) is shown.

(E) Same as D, except the ALPS mutant G156 lacking a sidechain (Substituted with R156) is shown in the A-A' loop of highlighted 2^nd^ FasL monomer (green).

(F) Indicated FasL mutants on top were added on to CD3 enriched T-cells in culture wells. After 30 minutes, soup and total lysates were pelleted together, followed by SDS-PAGE on non-reducing and partially denaturing gel and immunoblotting with anti-His antibody to detect recombinant FasL trimer formation on cells.

**Figure S6**

(A) Using immunoblotting total lysates from Jurkat, OVCAR3 and Hey-A8 cells were analyzed for cellular expression of Fas and indicated DISC regulatory components along with CD3 and HER2. GAPDH is loading control.

(B) Schematic sequence alignment of human Fas PPCR region for alanine scanning binding

and functional studies.

(C) Binding of increasing concentrations of E09 IgG1 against His-tagged indicated Fas mutants in a 96 well immobilized ELISA assay.

(D) The zoomed-in structure of Fas ECD in complex with E09 IgG1. The interface of the E09 Fas antibody against Fas PPCR is highlighted (PDB:3TJE). VH (green), VL (gold), and Fas (grey) are as depicted as ribbons. WT E09 contains GTGY (green sticks) in the CDR3 loop. GTGY of the CRD3 loop were mutated to AAAA, GGGG, or SSSS at 111-114 positions for the experiments described in E.

(E) WT E09 IgG1 or E09 with AAAA or GGGG or SSSS at 111-114 positions of E09 VH CRD3 were added onto OVCAR3 alone or in the presence of cycloheximide in cell survival analysis. FasL and KMTR2 were positive cell-killing controls (n=3+).

Error bars in (E) represent SEM (n=3+).

**Figure S7**

(A) Schematic showing genetic construction of generation of E09 IgG1 Fc-conjugated FasL bispecific molecule. Similar to the E09 variants tested in Figure S6d, e, additional variants were generated with mutations in E09 and FasL.

(B) CHO cells expressing indicated E09 IgG1, EP6 IgG1, E09-EP6 (IgG-scFv) and EP6-E09 (IgG-scFv) bispecific antibodies and E09 IgG1 Fc-conjugated FasL constructs were purified using protein-A and were run on reducing gel for size confirmation.

(C) Table showing expression (mg/0.5L) and cell killing activity of various different constructs described in Fig. 6, Fig. 7, Fig. S6 and Fig. S7.

(D) Increasing concentrations of either E09 IgG1 alone or E09-FasL bispecific combinations with GTGY, AAAA, GGGG, or SSSS at 111-114 positions of E09 VH CRD3 were added on to OVCAR3 cells followed by cell survival analysis after 36 hrs.

**Figure S8**

(A) OVCAR3 cells were cultured alone or either with CAR-T^DB^ (mock) or CAR-T^FOLR1^ cells E/T ratio of 6:1 for 12 hrs, followed by cell survival analysis (n=4).

(B) Schematic of CAR-T^FOLR1^ cells cocultured with the 50:50 mix of GFP+ colo-205 cells and ID8^Parental^ cells.

(C) The coculture in B was analyzed for GFP expressing using immunoblotting as an indicator of FOLR1+ colo-205 lysis. The decrease in GFP is an indicator of cell lysis, with GAPDH being loading control.

(D) Same as B and C, except the CAR-T^DB^ (mock) or CAR-T^FOLR1^ coculture plates were precoated with recombinant 5μg /ml FOLR1 (rFOLR1) or 5μg /ml recombinant Fas (rFas) as indicated.

(E) Schematic of CAR-T^FOLR1^ cells cocultured with the 50:50 mix of OVCAR3 and ID8^Parental^ cells.

(F-G) The cocultures in E were analyzed for caspase-8 cleavage and granzyme-B using immunoblotting ± rFas^WT^. Right: The highly saturated overexposed blot showed minimal basal caspase-8 cleavage, indicator of very minimal Fas signaling by CAR-T in antigen positive cells (OVCAR3 cells) as ID8^Parental^ line is negative against both human Fas and human FOLR1.

Error bars in (A) represent SEM (n=3+).

**Figure S9**

(A) Schematic of CAR-T^FOLR1^ cells cocultured with the 50:50 mix of ID8^GFP_huFas^ stable cells and OVCAR3 cells.

(B) Flow cytometry analysis of CD8 and GFP from the experiment described in A and Fig 8J.

(C) OVCAR3 and ID8^huFas^ stable cells either alone or in the presence of CAR-T^FOLR1^ cells were (E/T ratio of 5:1) cultured for 4 hrs followed by granzyme B measurement using ELISA.

(D) Immunoblotting analysis of SLC34A2 (NaPi2b) and FOLR1 in OVCAR3 vs Hey-A8 cells.

(E) CAR-T^NaPi2b^ cells were co-cultured for 0, 6 and 8 hrs either with OVCAR3 and Hey-A8^FasKO^ overexpressing exogenous Fas^WT^ (1:2:3 ratio) or with OVCAR3 and Hey-A8^FasKO^ overexpressing exogenous Fas^R87A^ (1:2:3 ratio). Total lysates of indicated combinations with indicated times were immunoblotted for caspase-8 cleavage, an indicator of bystander Fas signaling. The data of 1:2:2 ratio also resulted similar Fas activation (Fig 9g).

(F) Schematic of E09-FasL bispecific antibody treatment with the 50:50 mix of ID8^GFP_huFas^ stable and ID8^Parental^ cells or 100% ID8^GFP_huFas^ stable alone cells

Error bars in (C) represent SEM (n=3+).

**Figure S1**

**
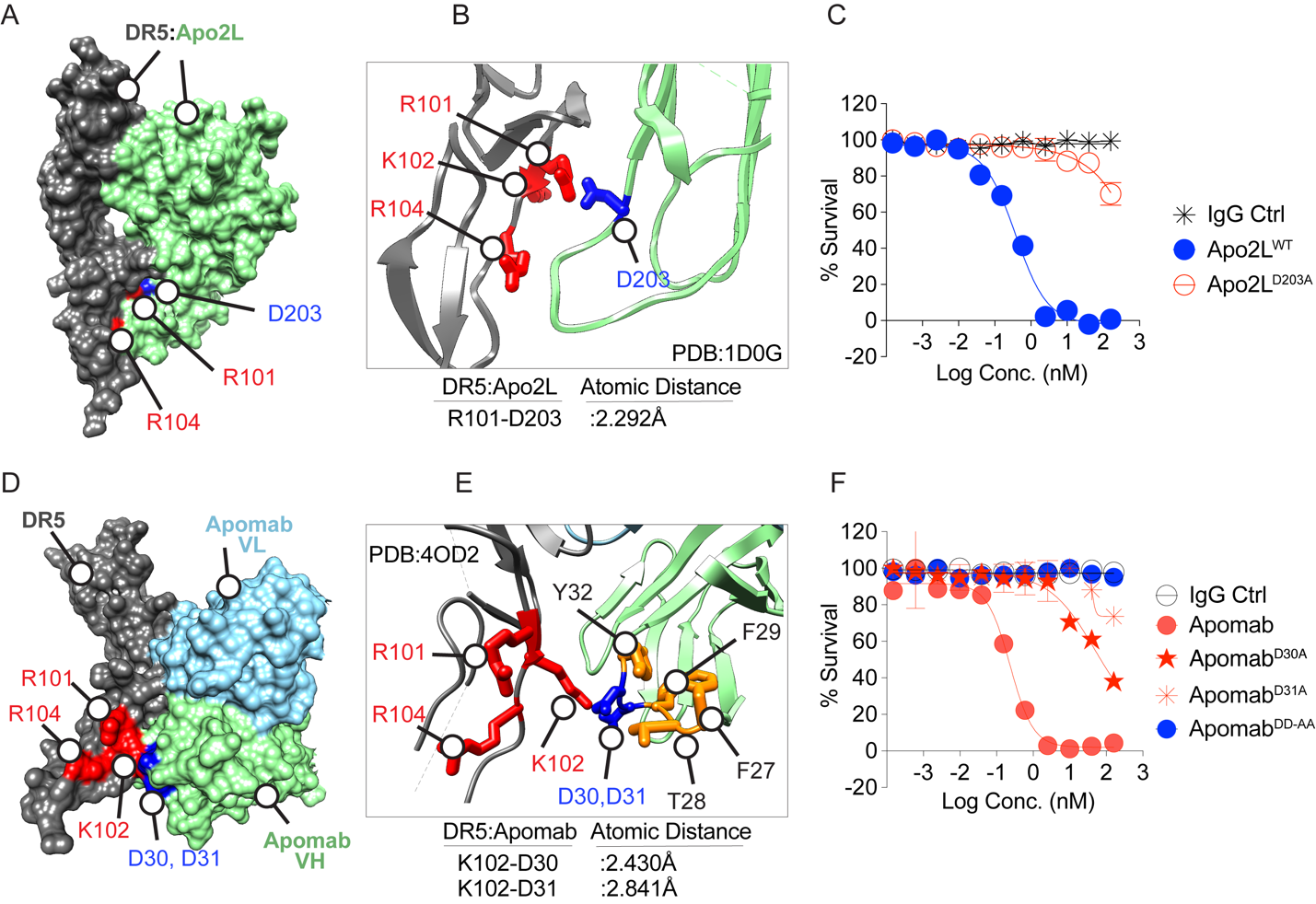
**


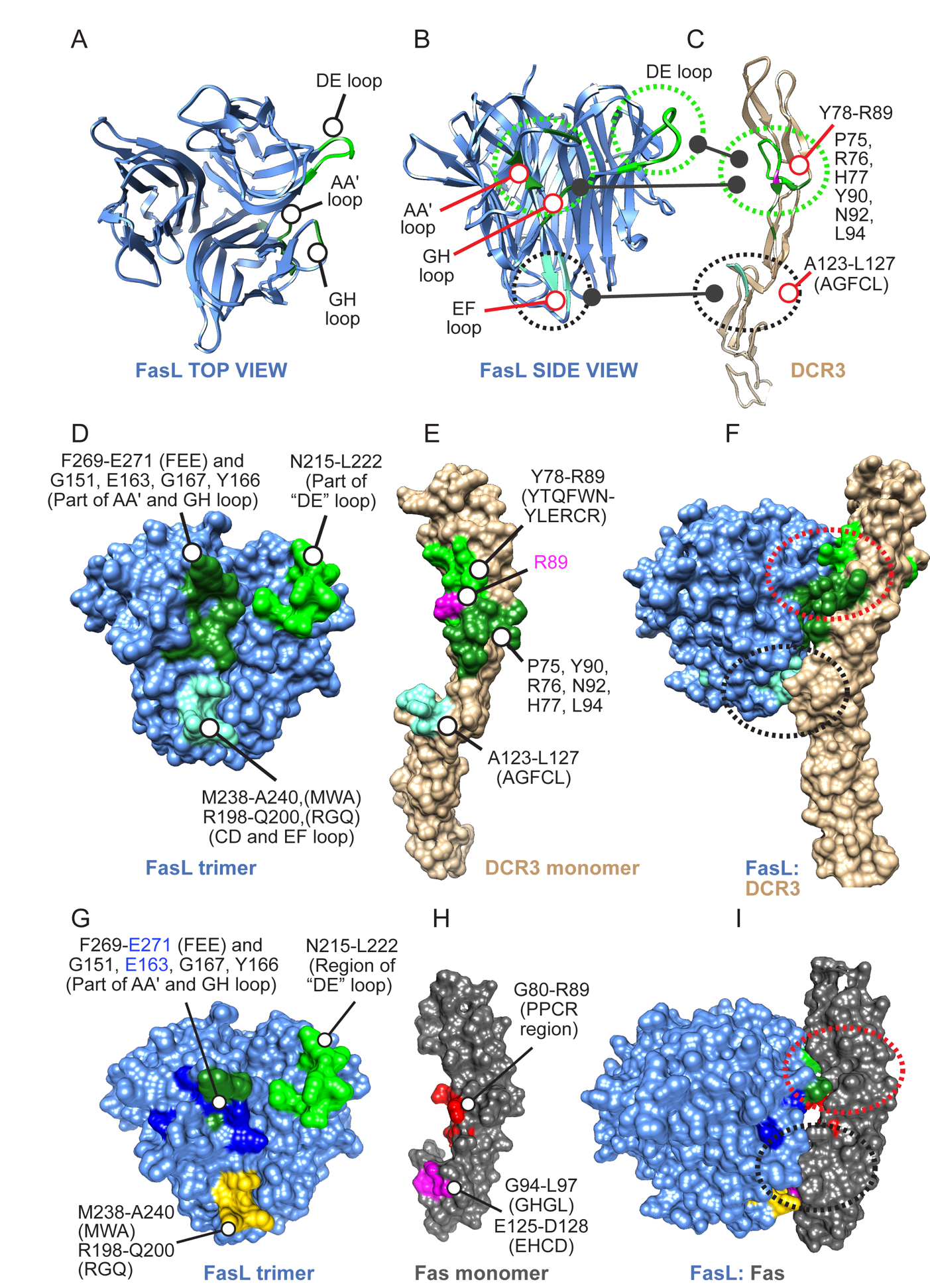
**Figure S2**

**Figure S3**


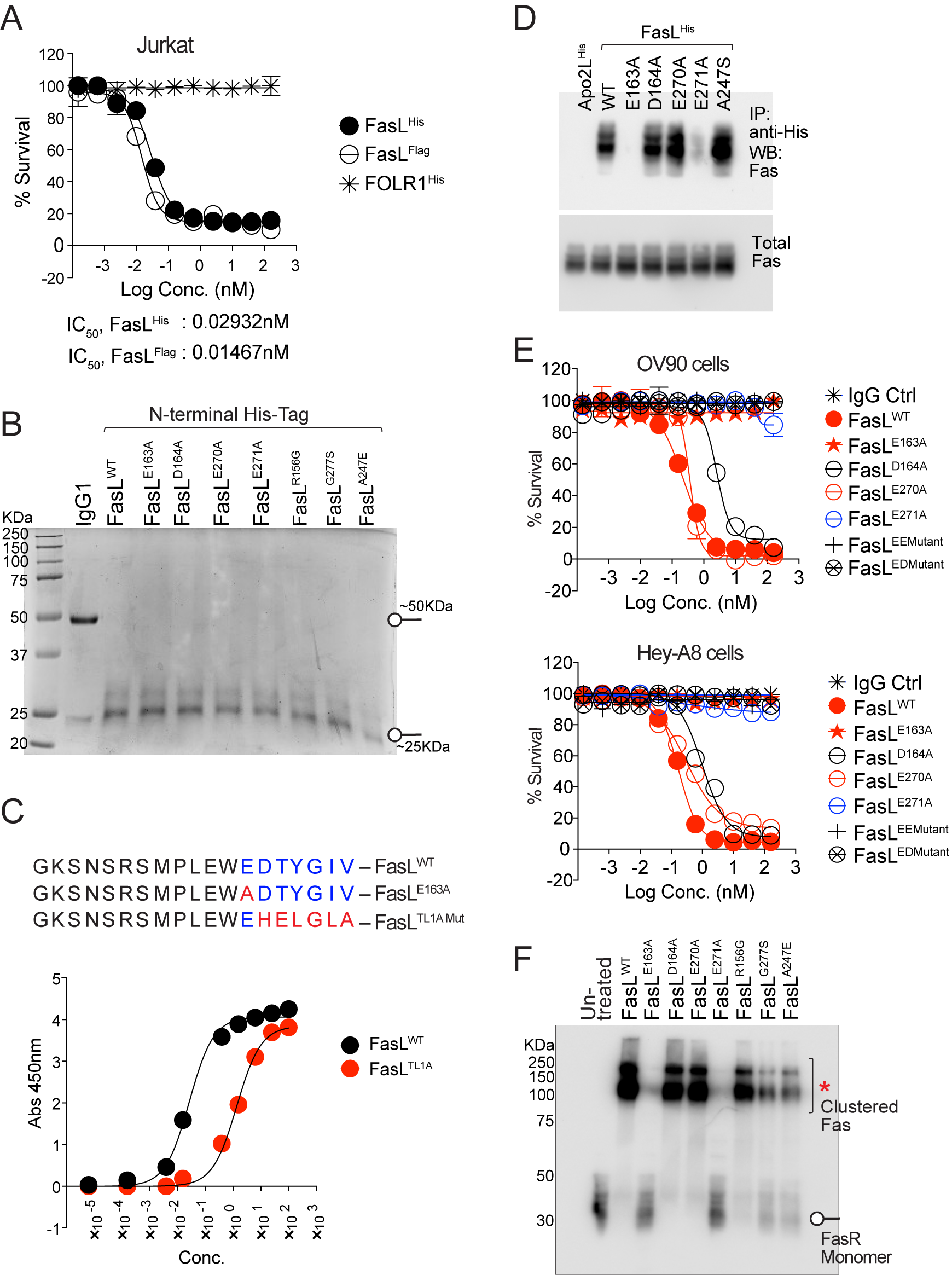


**Figure S4**


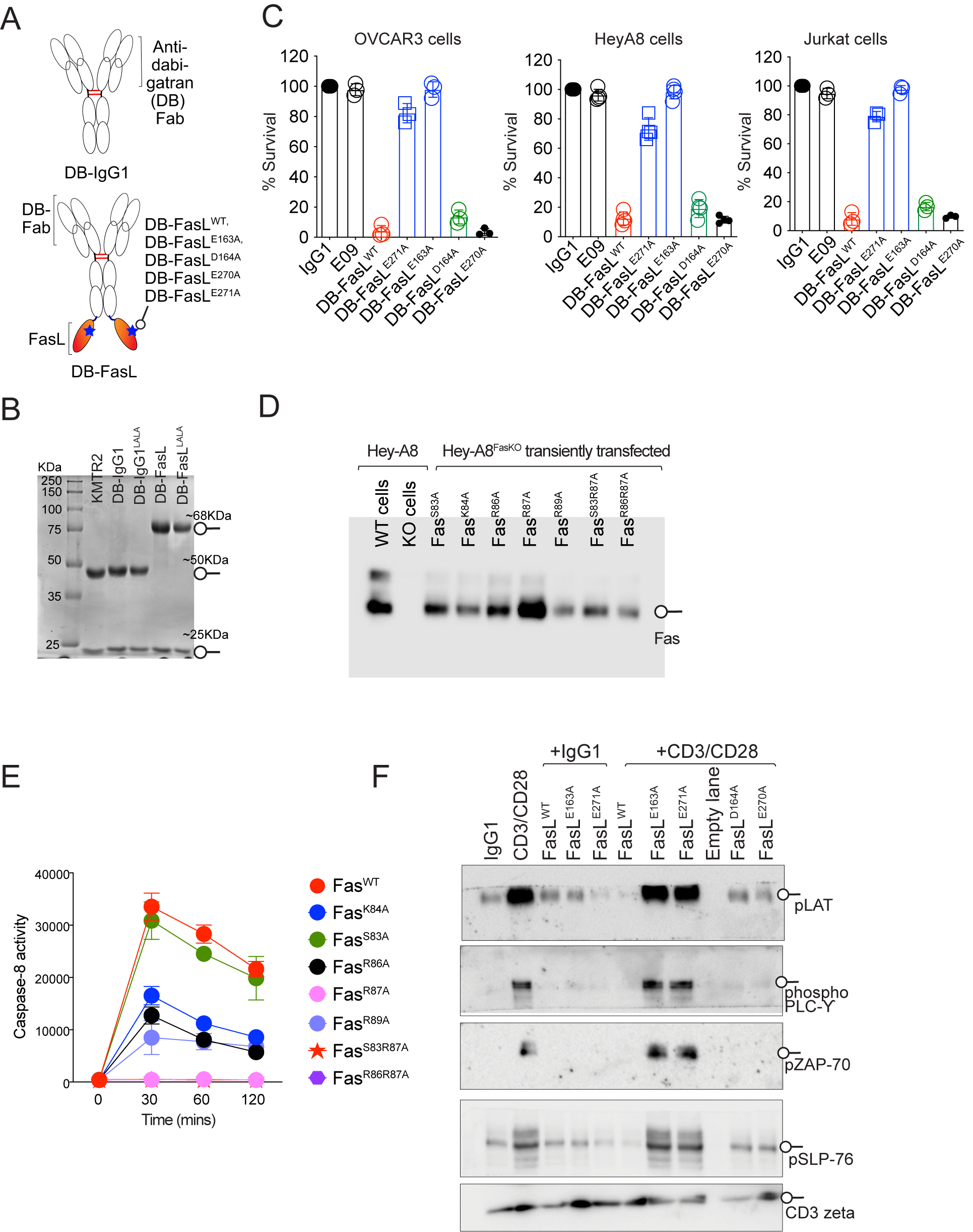


**Figure S5**


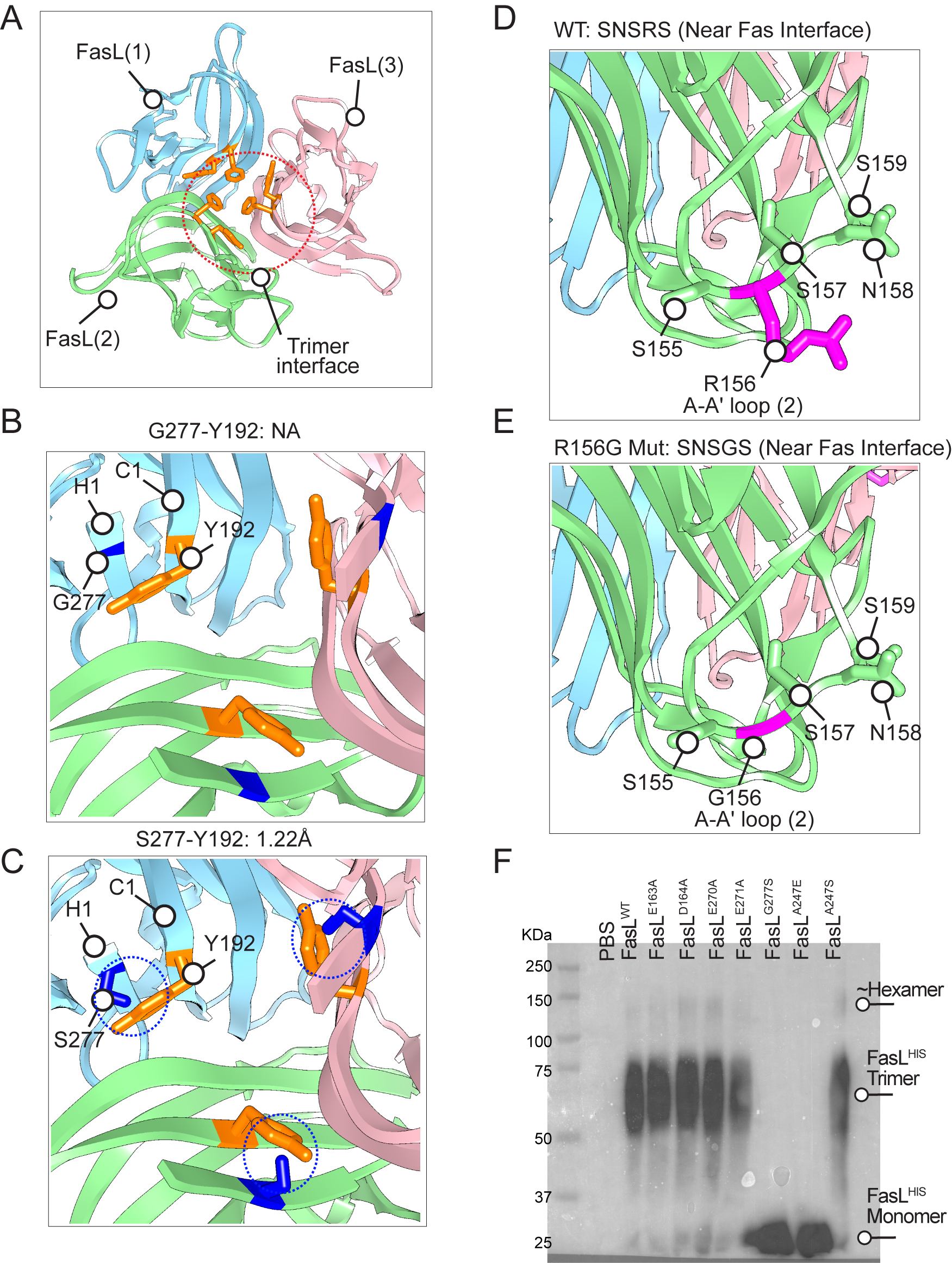


**Figure S6**


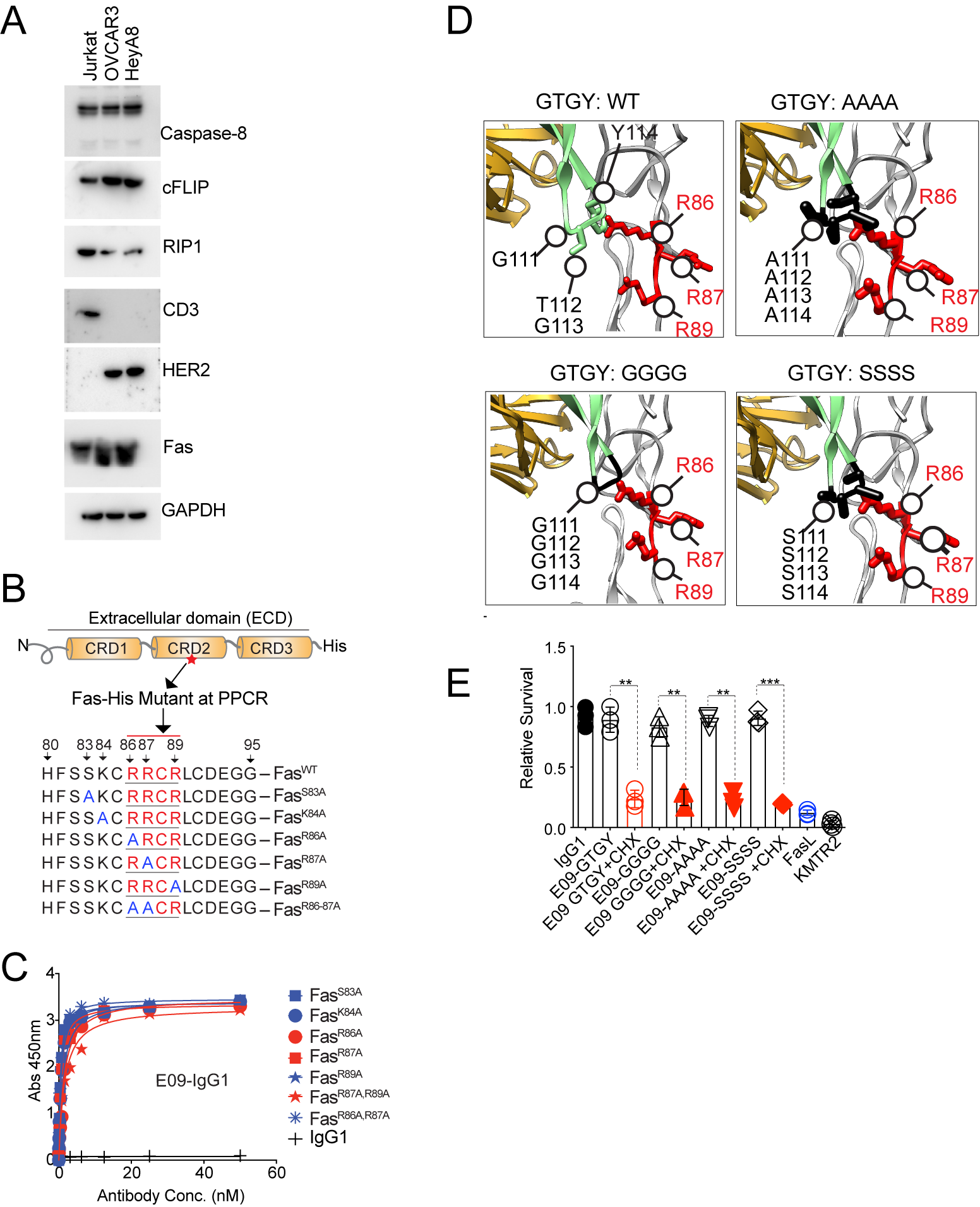


**Figure S7**


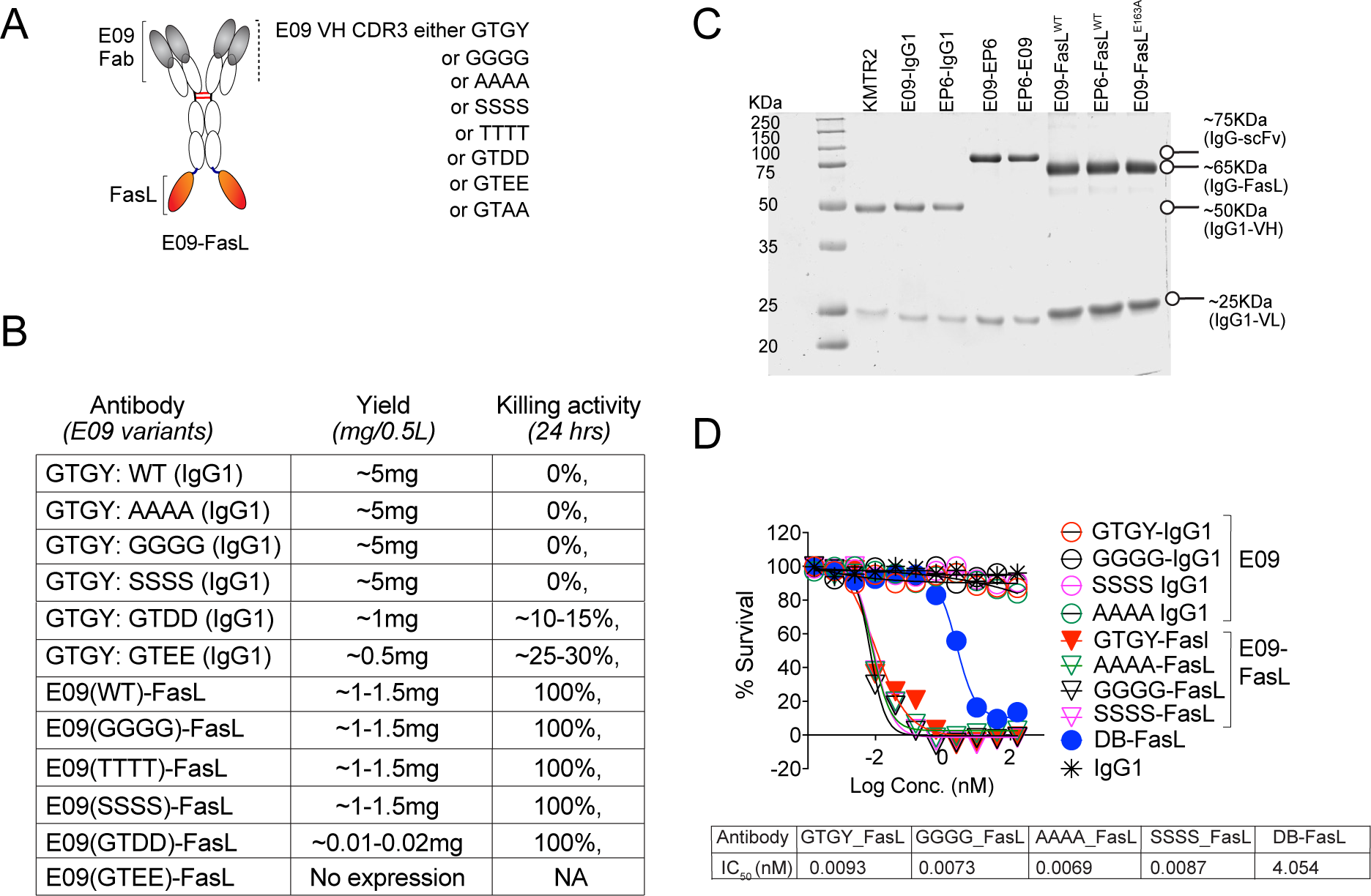


**Figure S8**


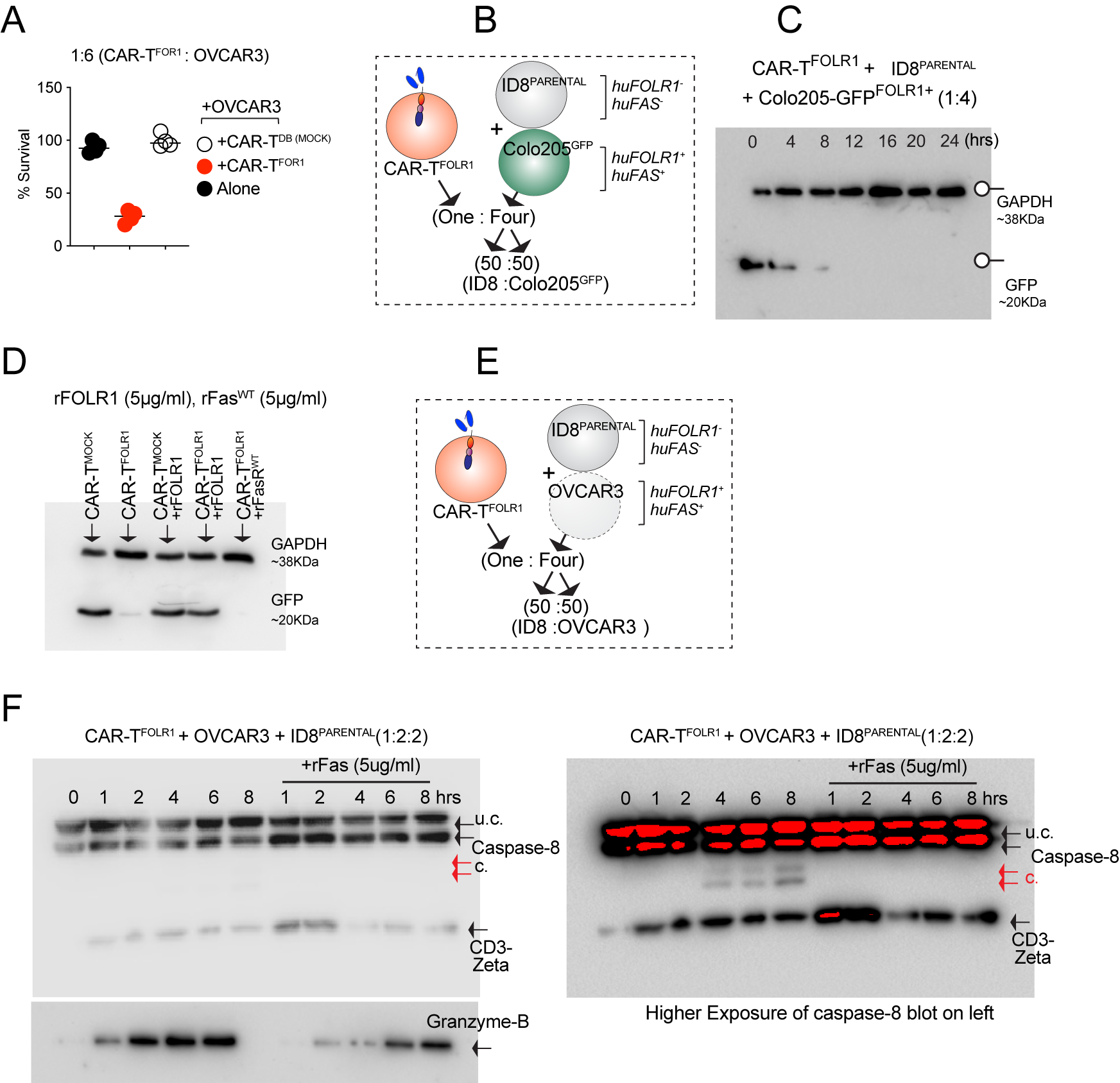


**Figure S9**


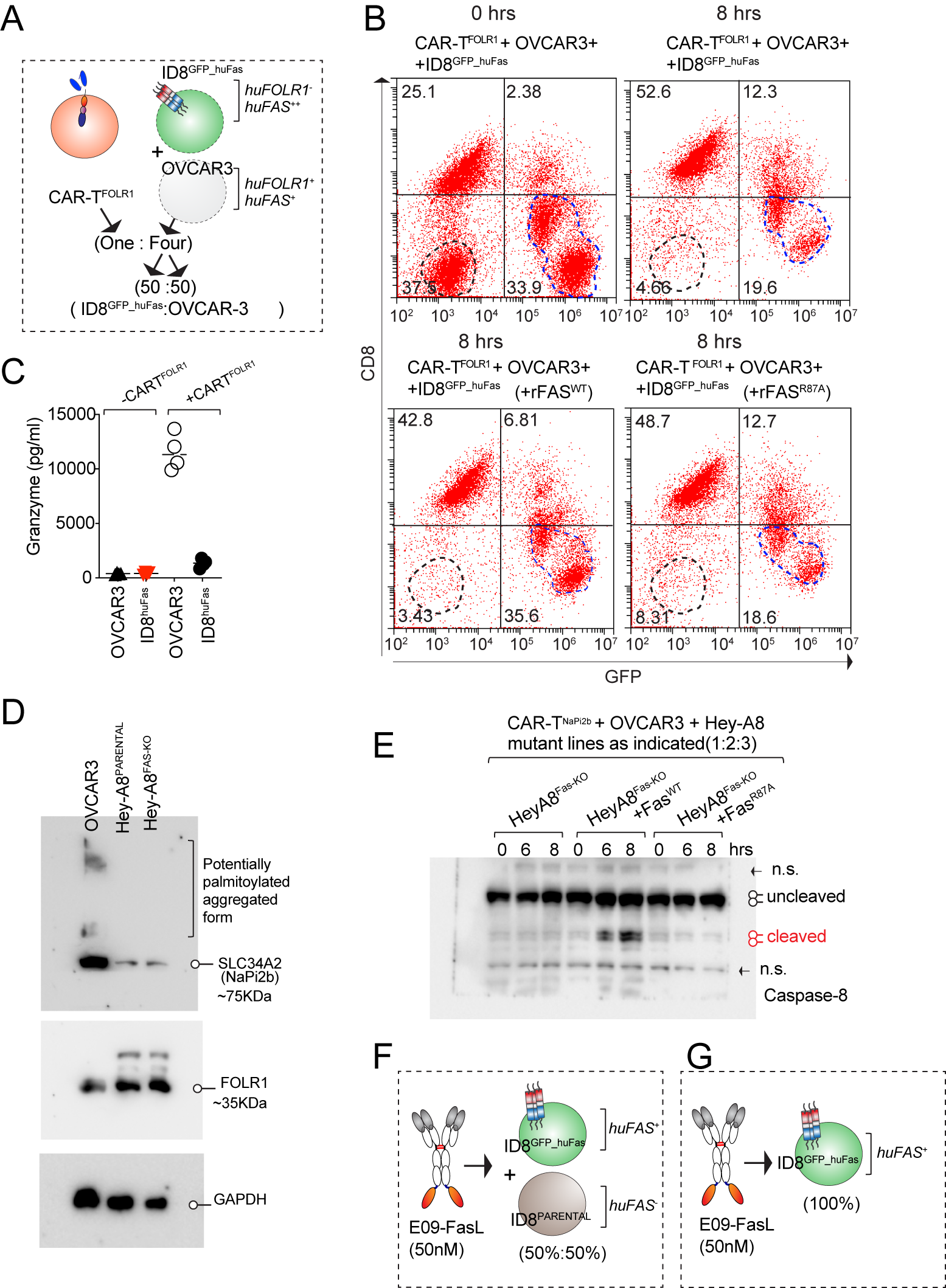

Supplement: Supplementary file 2 — Supplementary Figures and Legends [file 41418_2023_1229_MOESM2_ESM.docx]
