# Supplementary figures and images for: Characterizing the regulatory Fas (CD95) epitope critical for agonist antibody targeting and CAR-T bystander function in ovarian cancer
Source: Cell Death Differ. 2023 Oct 14;30(11):2408–31. doi: 10.1038/s41418-023-01229-7 (PMC10657439; doi:10.1038/s41418-023-01229-7)

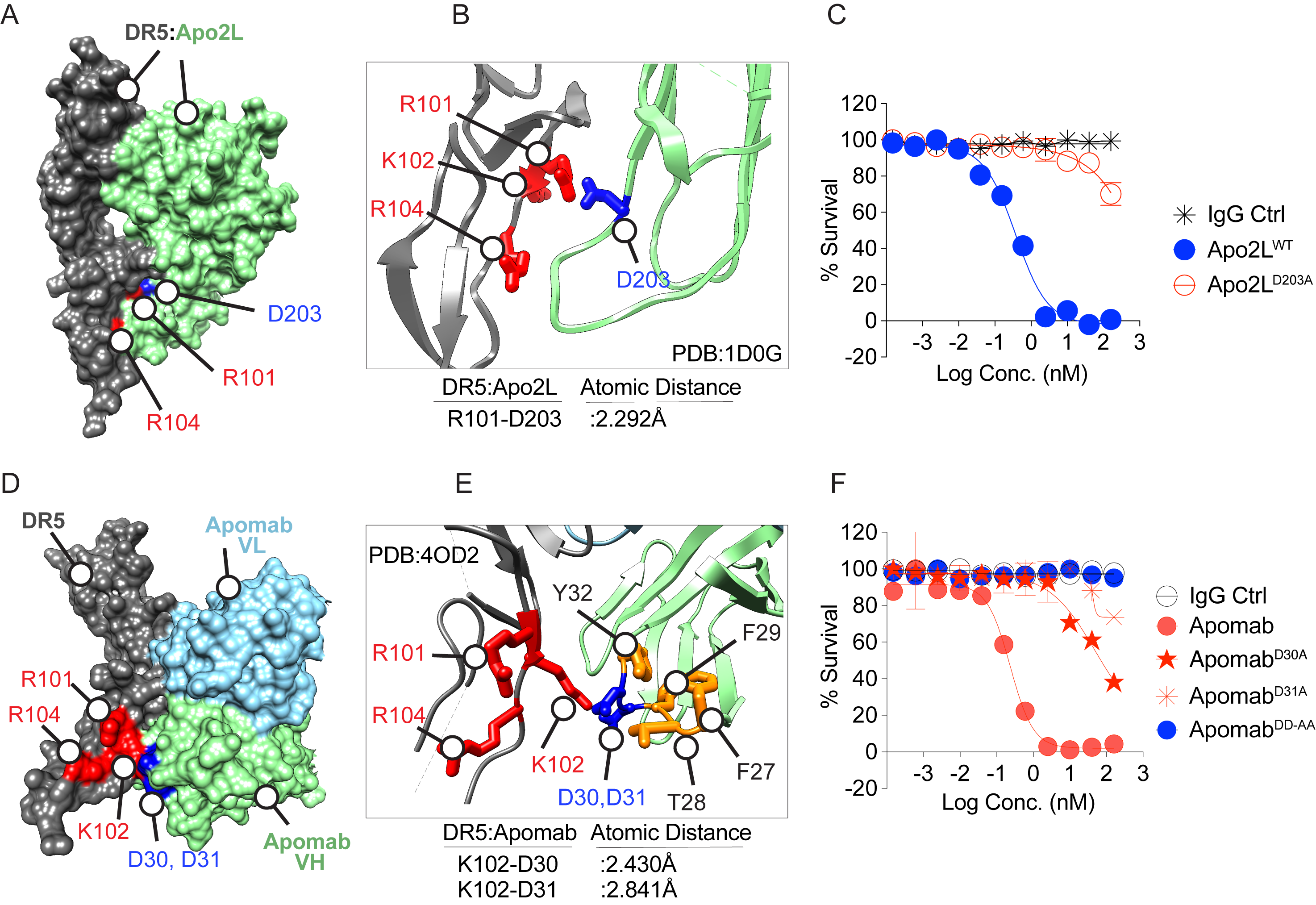

Supplement: Supplementary file 3 — Supplementary Figure 1, Figure S1 [file 41418_2023_1229_MOESM3_ESM.tif]

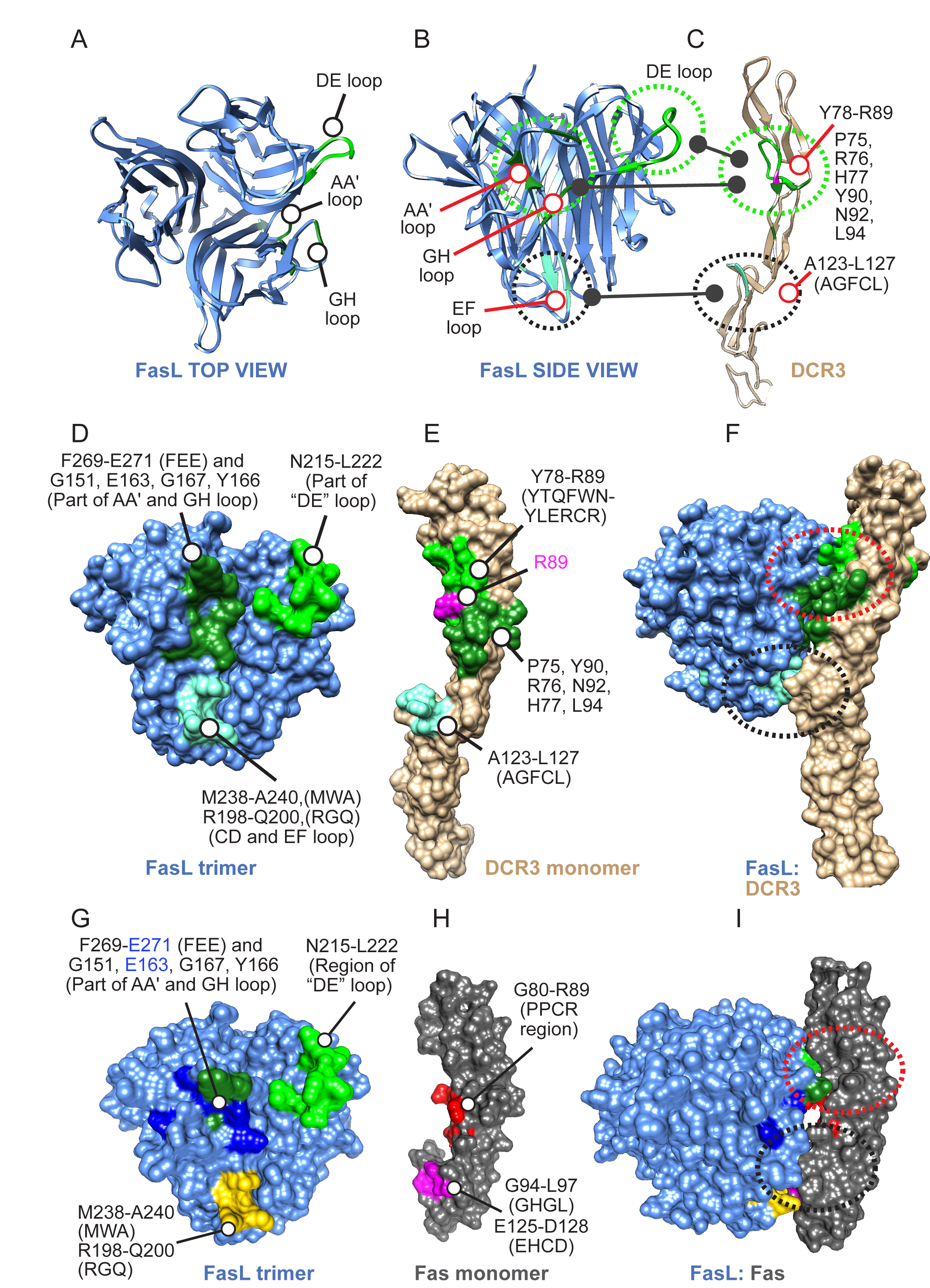

Supplement: Supplementary file 4 — Supplementary Figure 2, Figure S2 [file 41418_2023_1229_MOESM4_ESM.tif]

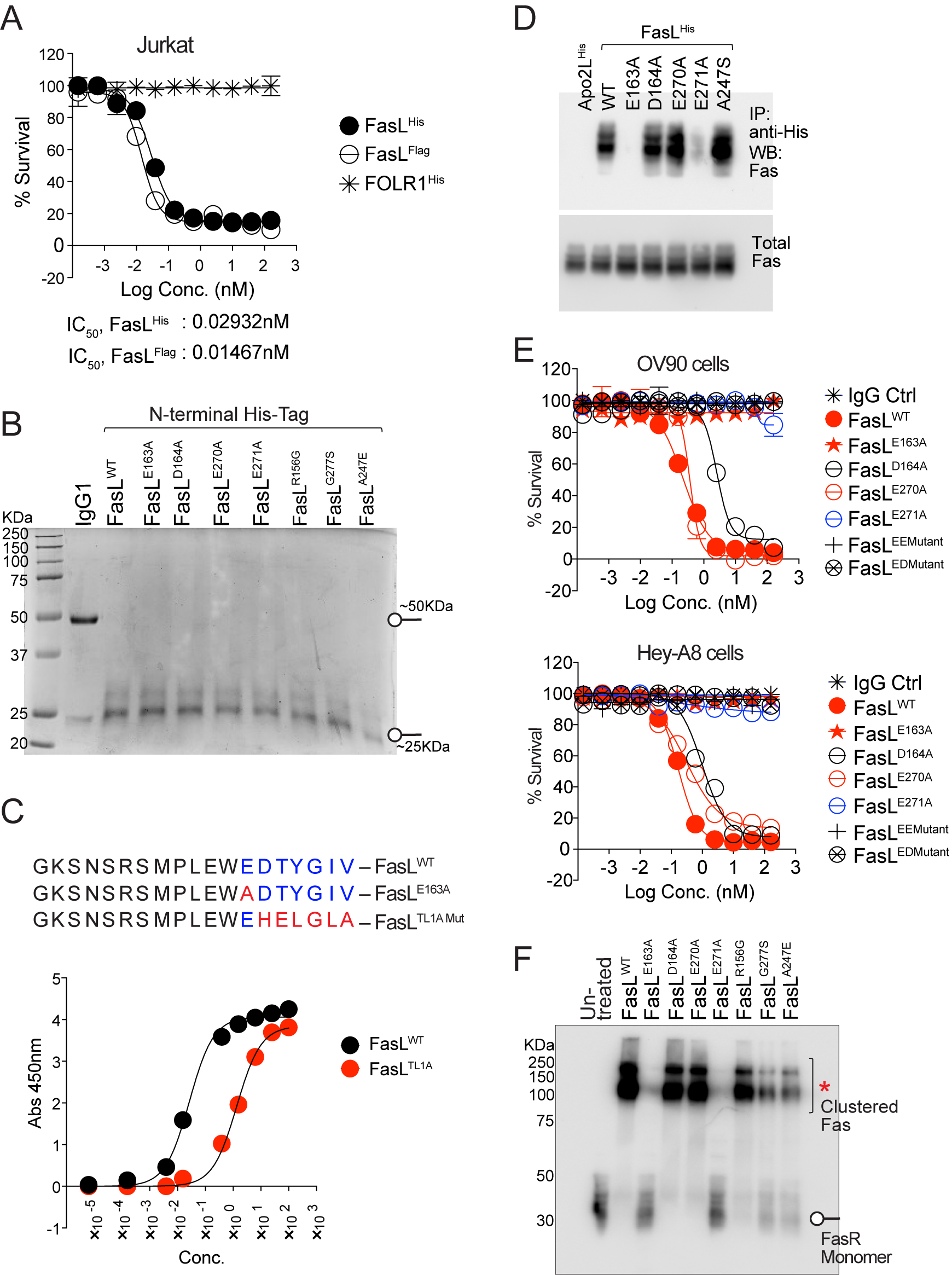

Supplement: Supplementary file 5 — Supplementary Figure 3, Figure S3 [file 41418_2023_1229_MOESM5_ESM.tif]

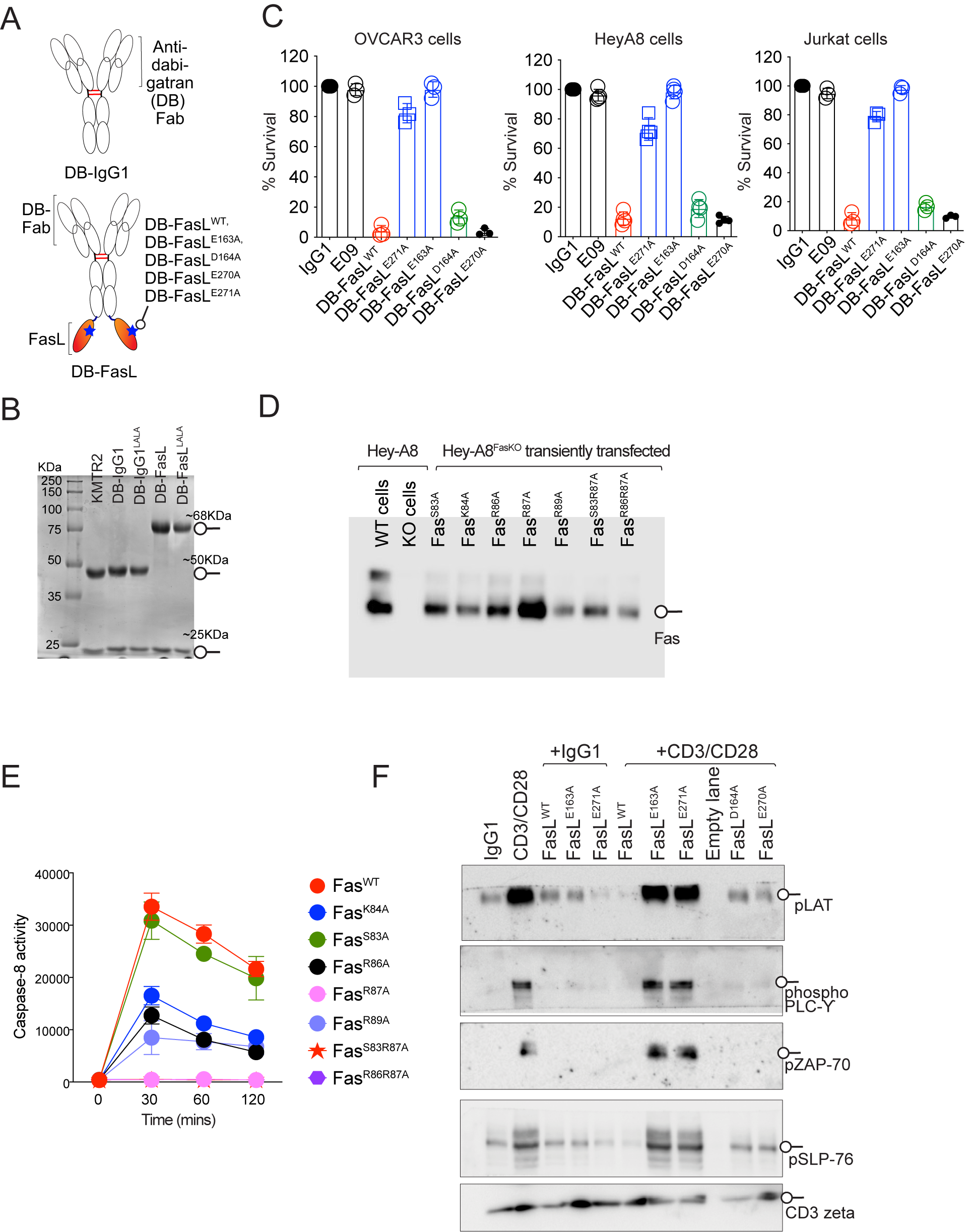

Supplement: Supplementary file 6 — Supplementary Figure 4, Figure S4 [file 41418_2023_1229_MOESM6_ESM.tif]

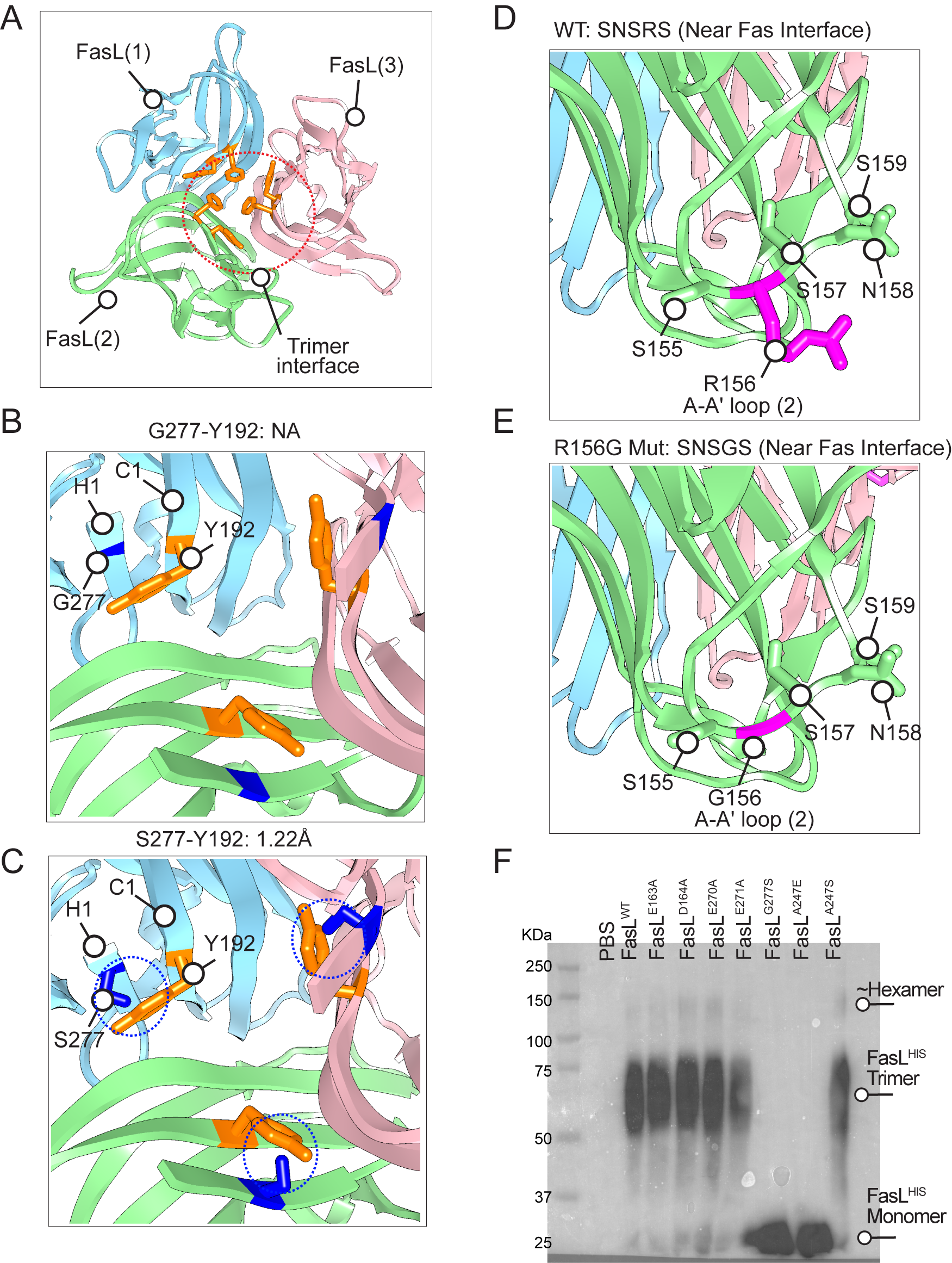

Supplement: Supplementary file 7 — Supplementary Figure 5, Figure S5 [file 41418_2023_1229_MOESM7_ESM.tif]

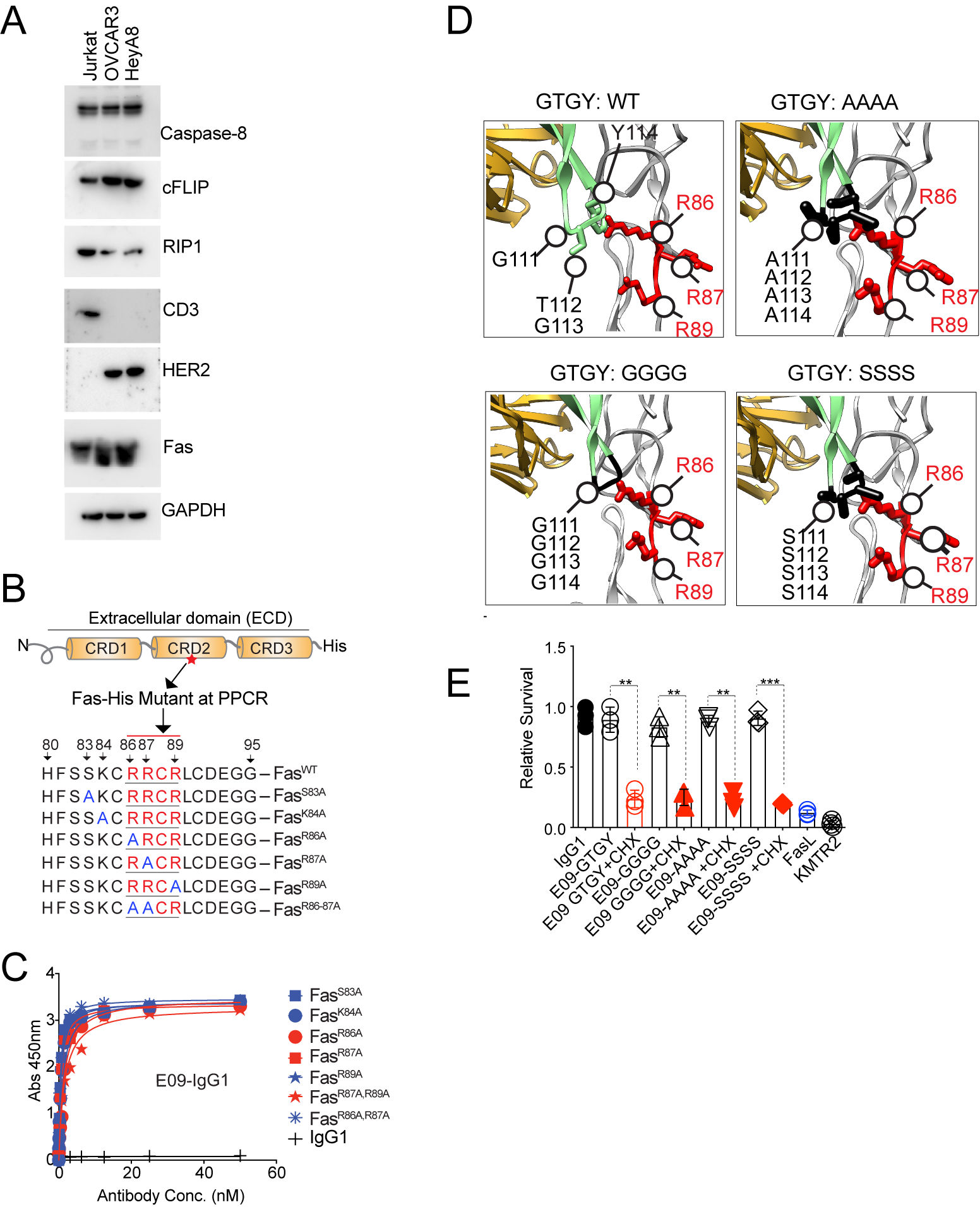

Supplement: Supplementary file 8 — Supplementary Figure 6, Figure S6 [file 41418_2023_1229_MOESM8_ESM.tif]

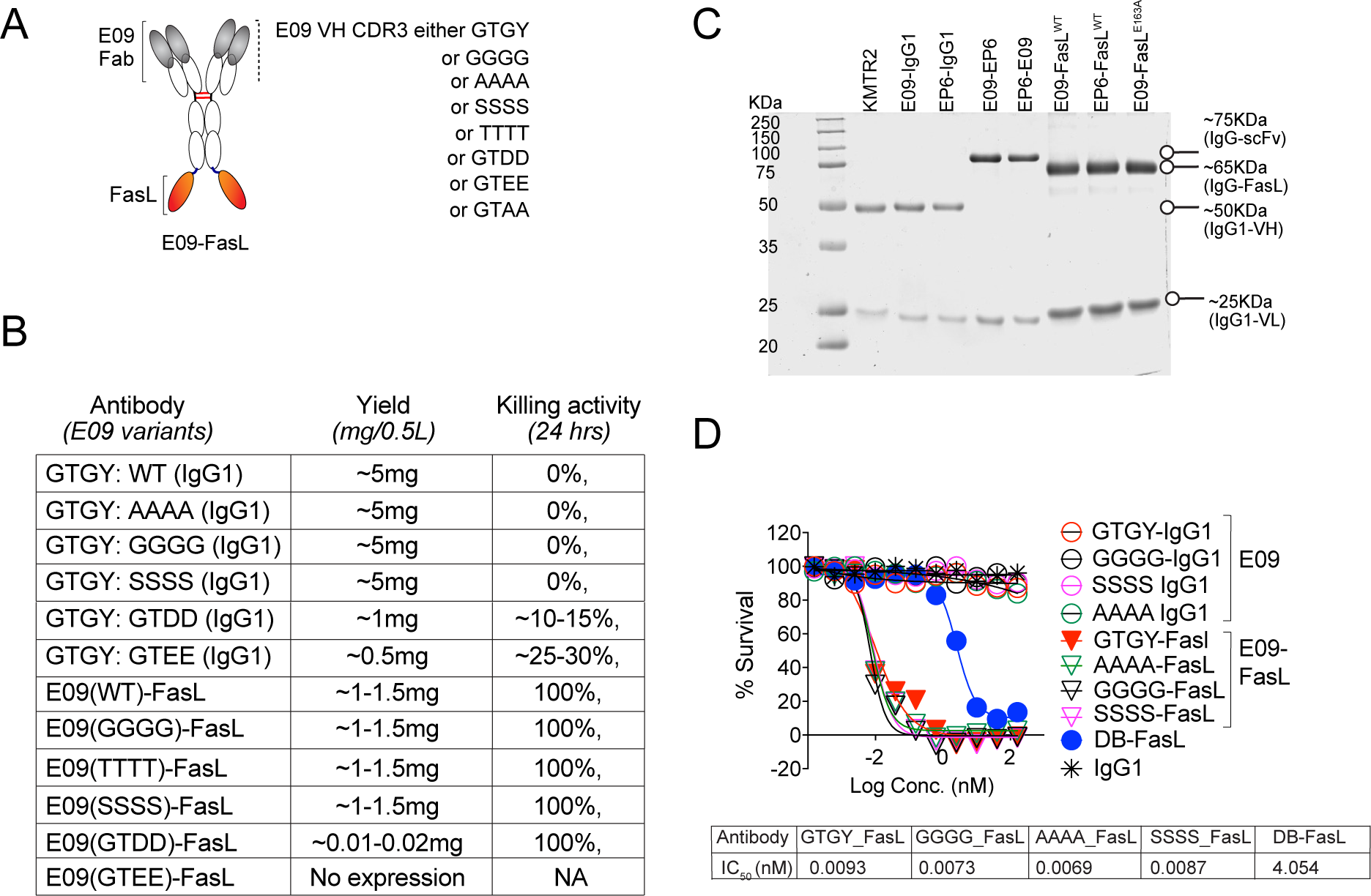

Supplement: Supplementary file 9 — Supplementary Figure 7, Figure S7 [file 41418_2023_1229_MOESM9_ESM.tif]

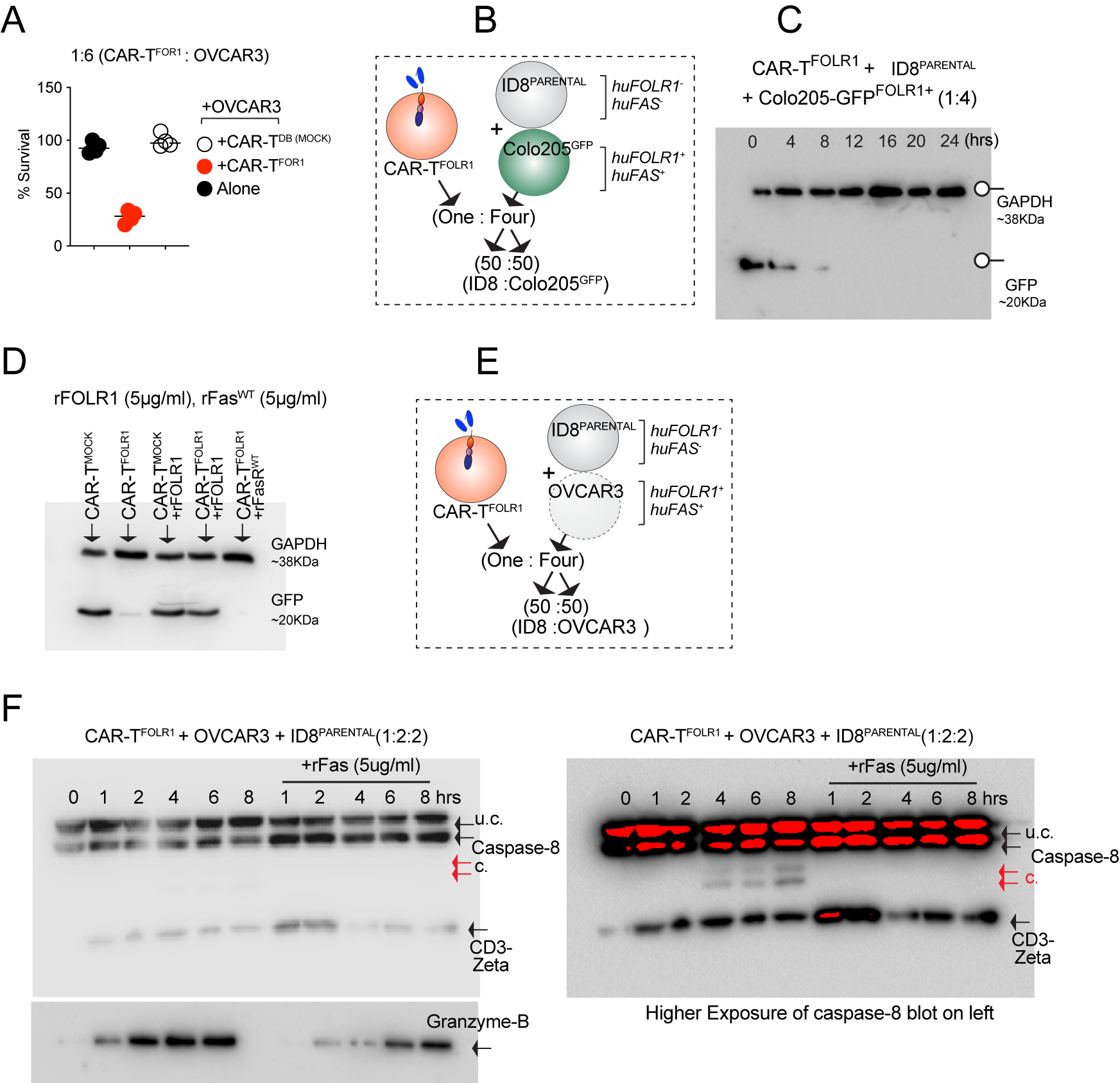

Supplement: Supplementary file 10 — Supplementary Figure 8, Figure S8 [file 41418_2023_1229_MOESM10_ESM.tif]

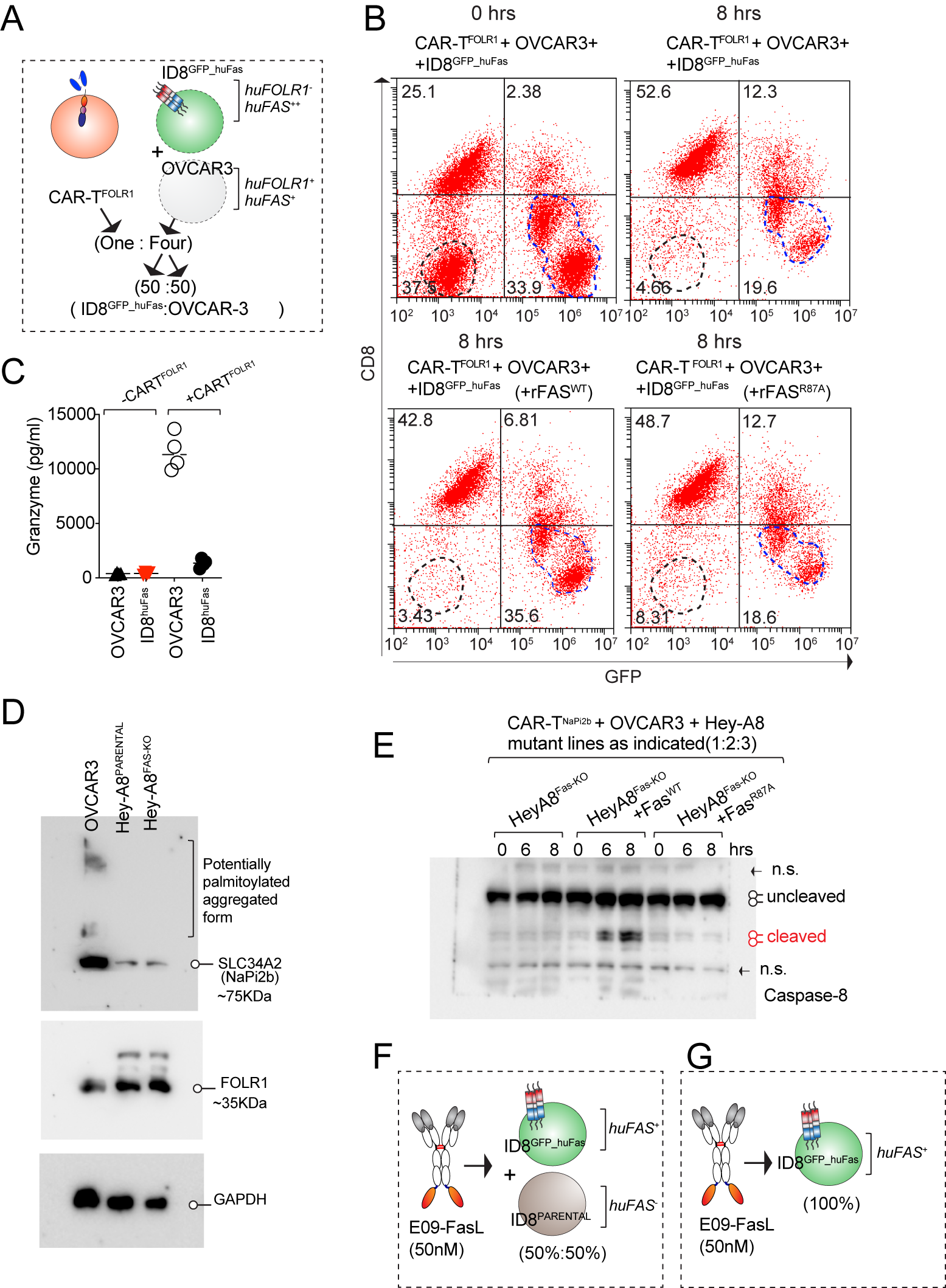

Supplement: Supplementary file 11 — Supplementary Figure 9, Figure S9 [file 41418_2023_1229_MOESM11_ESM.tif]
